# The bZIP Transcription Factor MoAP1 Mediates the Oxidative Stress Response and Is Critical for Pathogenicity of the Rice Blast Fungus *Magnaporthe oryzae*

**DOI:** 10.1371/journal.ppat.1001302

**Published:** 2011-02-24

**Authors:** Min Guo, Yue Chen, Yan Du, Yanhan Dong, Wang Guo, Su Zhai, Haifeng Zhang, Suomeng Dong, Zhengguang Zhang, Yuanchao Wang, Ping Wang, Xiaobo Zheng

**Affiliations:** 1 Department of Plant Pathology, College of Plant Protection, Nanjing Agricultural University, and Key Laboratory of Monitoring and Management of Crop Diseases and Pest Insects, Ministry of Agriculture, Nanjing, China; 2 Department of Pediatrics and the Research Institute for Children, Louisiana State University Health Sciences Center, New Orleans, Louisiana, United States of America; Carnegie Mellon University, United States of America

## Abstract

*Saccharomyces cerevisiae* Yap1 protein is an AP1-like transcription factor involved in the regulation of the oxidative stress response. An ortholog of Yap1, MoAP1, was recently identified from the rice blast fungus *Magnaporthe oryzae* genome. We found that MoAP1 is highly expressed in conidia and during invasive hyphal growth. The *Moap1* mutant was sensitive to H_2_O_2_, similar to *S. cerevisiae yap1* mutants, and MoAP1 complemented Yap1 function in resistance to H_2_O_2_, albeit partially. The *Moap1* mutant also exhibited various defects in aerial hyphal growth, mycelial branching, conidia formation, the production of extracellular peroxidases and laccases, and melanin pigmentation. Consequently, the *Moap1* mutant was unable to infect the host plant. The MoAP1-eGFP fusion protein is localized inside the nucleus upon exposure to H_2_O_2_, suggesting that MoAP1 also functions as a redox sensor. Moreover, through RNA sequence analysis, many MoAP1-regulated genes were identified, including several novel ones that were also involved in pathogenicity. Disruption of respective *MGG_01662* (*MoAAT*) and *MGG_02531* (encoding hypothetical protein) genes did not result in any detectable changes in conidial germination and appressorium formation but reduced pathogenicity, whereas the mutant strains of *MGG_01230* (*MoSSADH*) and *MGG_15157* (*MoACT*) showed marketed reductions in aerial hyphal growth, mycelial branching, and loss of conidiation as well as pathogenicity, similar to the *Moap1* mutant. Taken together, our studies identify MoAP1 as a positive transcription factor that regulates transcriptions of *MGG_01662*, *MGG_02531*, *MGG_01230*, and *MGG_15157* that are important in the growth, development, and pathogenicity of *M. oryzae*.

## Author Summary

*Magnaporthe oryzae* is a causal agent of rice blast disease and an important model for understanding of fungal development and pathogenicity. To examine the molecular mechanisms involved in conidium formation and appressorium development of *M. oryzae*, we identified the transcriptional factor MoAP1 as a regulator of the oxidative stress response. Our results indicated that MoAP1 is a stage-specific regulator for conidium formation, morphology, aerial hyphal growth, and also growth *in planta*. Additionally, we identified four novel genes whose functions were linked to MoAP1 and pathogenicity. Disruption of *MGG_01662* (encoding aminobutyrate aminotransferase, MoAat) and *MGG_02531* (hypothetical protein) caused minor phenotypic changes but attenuated virulence, and disruption of *MGG_01230* (encoding succinic semialdehyde dehydrogenase, MoSsadh) and *MGG_15157* (encoding acetyltransferase, MoAct) resulted in drastic reductions in the growth of aerial hyphae and hyphal branching as well as loss of conidiation and pathogenicity. Our studies extend the current understanding of AP1 functions in fungi and reveal that the MoAP1-mediated regulatory network is associated with the pathogenicity of *M. oryzae*.

## Introduction

Organisms such as plants have evolved to develop many efficient defense systems against pathogenic microbes. Among them, reactive oxygen species (ROS), primarily superoxide and H_2_O_2_, produced by plasma membrane-localized NADPH oxidases [1], are regarded as one of the fastest defense reactions against pathogen attack [2]. During the plant defense response, ROS was used by apoplastic peroxidases on the cell wall to synthesize lignin and other phenolic polymers that prevent pathogen invasion into the host [3]. There are reports that ROS produced at the site of an attempted invasion may also function as a second messenger in the induction of various plant defense-related genes and is essential for the pathogen-associated molecular pattern (PAMP) triggered immunity (PTI) response in plants [4], [5], [6], [7]. Additionally, due to the toxicity, ROS accumulated at the site of pathogen invasion can directly kill the pathogen [8], [9]. Conversely, plant pathogens have also developed many strategies, including enzymatic and non-enzymatic ones, to detoxify ROS and successfully invade their hosts [10], [11], [12], [13]. The relative sensitivity of the fungal pathogen to ROS may also depend on the effectiveness of its own ROS detoxification or tolerance machinery. In fungal pathogens, transcription factor-mediated host-derived ROS detoxification through regulation of gene expression is important in plant-microbe interactions [14], [15], [16]. Detoxifying enzymes, either preformed or inducible, including superoxide dismutase, catalases, and peroxidases, are thought to contribute to the tolerance of ROS in pathogenic fungi [15], [17], [18], [19].

*Saccharomyces cerevisiae* transcription factor Yap1 functions as one of the most important determinants of the yeast's response to the oxidative stress and Yap1 is responsible for transcriptional activation of various genes involved in ROS detoxification [20], [21], [22], [23], [24]. Loss of Yap1 function resulted in increased sensitivity to external stresses. A comparison of AP1 transcription factors from eukaryotic organisms revealed a conserved N-terminal basic leucine zipper (bZIP) DNA-binding domain, consisting of a leucine zipper that mediates dimerization [25] and an adjacent basic region that specifically interacts with DNA sequences [24]. At the C-terminus, the cysteine-rich domains (c-CRD) are highly conserved [26] and play a key role in Yap1-mediated resistance to the oxidative stress and, together with an n-CRD, for the appropriate subcellular localization of the Yap1 protein [27], [28]. Additionally, mutation of cysteine residues in Yap1 resulted in increased sensitivity to a variety of oxidizing compounds and drugs [29]. To date, Yap1 homologs were identified in several fungal pathogens [14], [15], [30], [31], [32], which share the function in stress tolerance but differ in pathogenicity. Yap1-mediated ROS detoxification was an essential virulence determinant in *Ustilago maydis* [15], *Alternaria alternata* [14], and *Candida albicans* [31], but it had no role in virulence of *Cochliobolus heterostrophus* and *Aspergillus fumigatus* [30], [32].

*Magnaporthe oryzae* is a pathogen of both economical and scientific importance [33], [34]. Like most other fungal pathogens, conidia of *M. oryzae* play a central role in the disease cycle. When attached on the host surface, conidia begin to germinate and develop appressoria from the end of the germ tubes [35]. The mature appressorium generates enormous turgor pressure (8 MPa) to help penetrate the plant cuticle and enter the plant cells [36]. After penetration, infection hypha spread through the rice leaf cells and typical necrotic lesions develop on the surface of rice leaves. Eventually, aerial conidiophores differentiate from hyphae on the lesion and newly formed conidia are released to serve as secondary inocula for new infections. In the past two decades, efforts have been made to study the conidiation process, formation of appressoria, and host plant responses to infection. Studies have suggested that *M. oryzae* infectious hyphae is biotrophic and it secretes effectors, such as biotrophy-associated secreted (BAS) proteins that can alter host cellular defense processes [37], [38]. The availability of genome sequences for both *M. oryzae* and rice host provided a new platform to identify pathogenicity-related genes and to understand molecular pathogenesis at the genome level [39], [40].

In this study, we identified MoAP1 as a homolog of the bZIP transcription factor AP1. We also identified four other proteins as the downstream targets of MoAP1, which were also involved in conidiation and pathogenicity.

## Results

### Identification of MoAP1 from *M. oryzae*

Using *S. cerevisiae* Yap1 sequence as trace, we identified the locus MGG_12814 (XP_001408783) from the *M. oryzae* genome (http://www.broad.mit.edu/annotation/genome/magnaporthe_grisea/Home.html) [40]. MGG_12814 was predicted to encode a 576-amino acid (aa) protein that shares substantial similarity (19–50%) to a number of fungal AP1 proteins. We thus named the protein MoAP1. Analysis of MoAP1 showed several conserved domains including a bZIP DNA-binding domain, a nuclear localization domain near the N-terminus (aa 150–214), and a cysteine-rich domain at the C-terminus (c-CRD; aa 492–551). Alignments of MoAP1 with other Yap1-like proteins revealed high similarity in the bZIP and c-CRD domains (see Figure S1A and S1B). The alignments of the bZIP domains also revealed the most conserved residues Q161, N162, A165, Q166, A168, F169, and R170 in the basic region (see Figure S1A). The c-CRD domain is also rich in cysteine (C506, C530, and C539) and serine (S540) residues, and a putative nuclear export sequence (NES) within the c-CRD (aa 526–535) is a possible binding site for the Crm1p-like exporter (see Figure S1B) [41]. The phylogenetic relationship of MoAP1 to other AP1 proteins revealed that AP1-like proteins in filamentous fungi apparently separated from those of unicellular yeasts, with *M. oryzae* MoAP1 most similar to *Gibberella zeae* GzAP1 (XP_388976) and *Fusarium oxysporum* FoAP1 (XP_388976) (see Figure S1C).

### MoAP1 complemented the growth defect of a yeast *yap1* mutant under oxidative conditions

To determine the function of MoAP1, we tested whether MoAP1 complements a *yap1* mutant of *S. cerevisiae*. An expression vector pYES2 containing the full-length *MoAP1* gene was transformed into the *yap1* mutant. As a control, the *yap1* mutant was also transformed with an empty pYES2 vector. When plated on glucose- (suppression) or galactose-containing (induction) medium in the absence of oxidizing agents, no growth defect was observed (see Figure S2). However, when 0.3 mM hydrogen peroxide (H_2_O_2_) was added, only the wild-type strain grew on glucose-containing medium. On medium containing both 0.3 mM H_2_O_2_ and galactose, the *yap1*/pYES::*MoAP1* formed colonies similar to the wild type, while the *yap1* mutant carrying the empty pYES2 vector was significantly inhibited (see Figure S2). This indicated that the ability of the *S. cerevisiae yap1* mutant to cope with H_2_O_2_ stress could be complemented by the introduction of *MoAP1*. However, judging by growth, the complementation appeared to be partial (see Figure S2).

### *MoAP1* is highly expressed in conidia and in invasive hyphae

To explore the function of MoAP1, we examined its expression in *M. oryzae* by quantitative real-time polymerase chain reaction (qRT-PCR, see Figure S3A). The abundance of *MoAP1* transcripts during vegetative growth in liquid CM was relatively lower than in conidia, when compared with the expression of the control actin gene (*MGG_03982.5*) (avg. dCt  = 6.0). In the invasive growth *in planta*, *MoAP1* mRNA accumulation decreased almost two-fold at 8 hrs and 11-fold at 24 hrs post-inoculation (hpi) on rice leaves, compared with that of conidia at 0 hpi. However, the transcript abundance began to increase after 24 hpi, reaching almost the same level as that of conidia at 72 hpi (see Figure S3A). Thus, we concluded that the *MoAP1* was highly activated during conidiation and infection.

### MoAP1 is necessary for aerial hyphal growth but not mycelial radial growth

To understand the role of MoAP1 in growth, we generated a *Moap1* mutant strain using the *Moap1* disruption allele linked to a hygromycin resistance marker gene (see Figure S3B). Linear DNA fragments amplified from the *Moap1* disruption allele were used to transform the protoplasts of the wild type strain Guy11. Putative transformants were screened on hygromycin media and verified by PCR amplification (see Figure S3C). The mutants were also confirmed by RT-PCR (see Figure S3D) and Southern blotting analysis (see Figure S3E and S3F). Two deletion mutants, *Moap1-9* and *Moap1-20*, were selected for further analysis. To complement the mutant strain, the genomic DNA sequence of *MoAP1* containing a 2-kb promoter region was reintroduced into the *Moap1* mutant and verified using PCR amplification (see Figure S3C and S3D).

**Figure 1:**
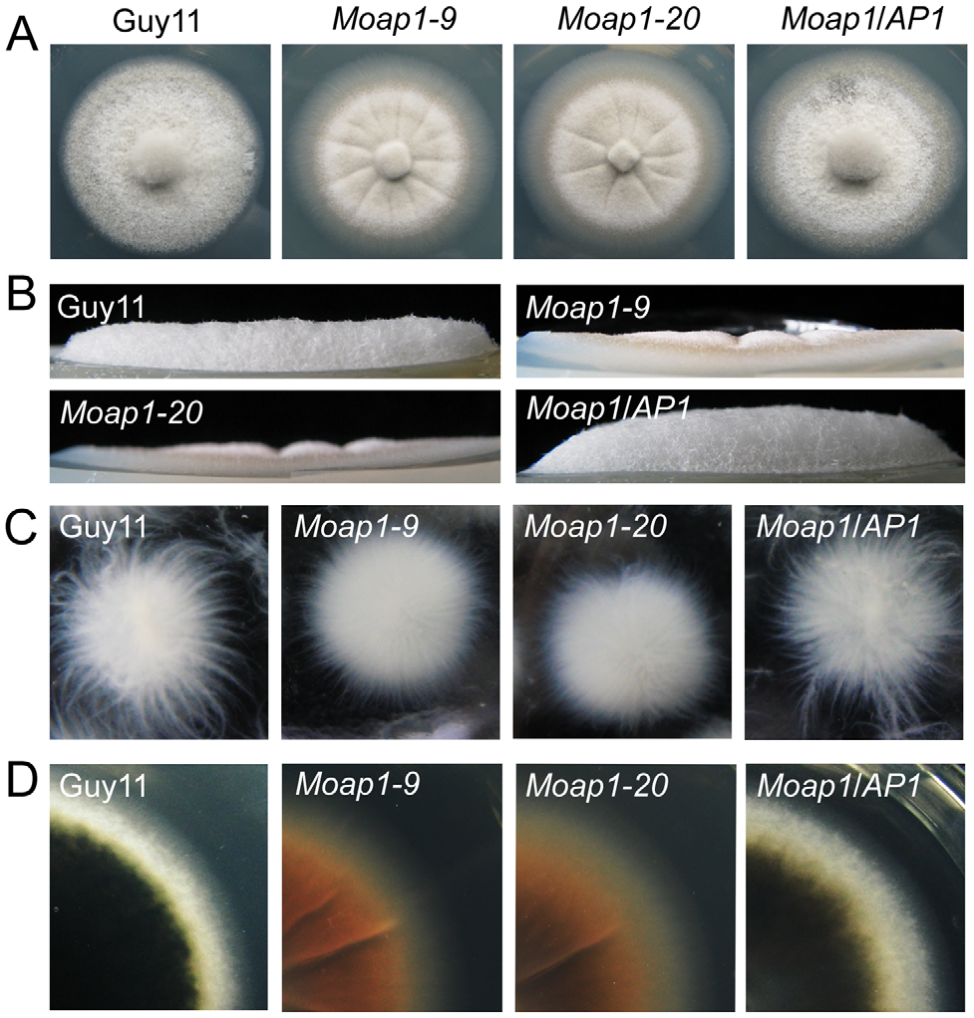
The effect of MoAP1 on mycelia growth. (A) Mycelial growth is not altered in the *Moap1* deletion mutant. The *Moap1* mutants, the wild type strain (Guy11) and complemented strain (*Moap1/AP1*) were inoculated on CM medium and cultured at 28°C in darkness for 5 days. (B) Aerial hyphae growth is reduced in the *Moap1* mutants. Strains were grown under the same conditions as above and colony side views are displayed. (C) Phenotype of mycelia growth in liquid CM medium. The *Moap1* mutants, Guy11, and the complemented strains were inoculated in liquid CM medium for 48 hrs at 28°C in darkness and then photographed. (D). Colony pigmentation is compromised in the *Moap1* mutants. The testing strains were cultured as described in Figure 1A and photographed.

On CM medium for 5 days at 28°C, the *Moap1* mutants showed no apparent defect in radial growth, but presented as a flat colony due to the reduced aerial hyphal growth (Figure 1A and 1B) with altered pigmentation (Figure 1D). We further incubated Guy11, *Moap1* mutants, and the complemented strain in liquid CM for 48 hrs and found that the mutant strains formed compact mycelia masses, in contrast to the sparse ones formed by the wild-type and complemented strains (Figure 1C). We also compared hyphal branching patterns of the *Moap1* mutants with the control strains using Calcofluor white (CFW) staining and found that the hyphal branching was severely reduced in the *Moap1* mutant (see Figure S4A). Together, these results indicated that MoAP1 is essential for proper growth and hyphal branching.

### *MoAP1* disruption resulted in abnormal conidium morphology and reduction in conidia formation

**Figure 2:**
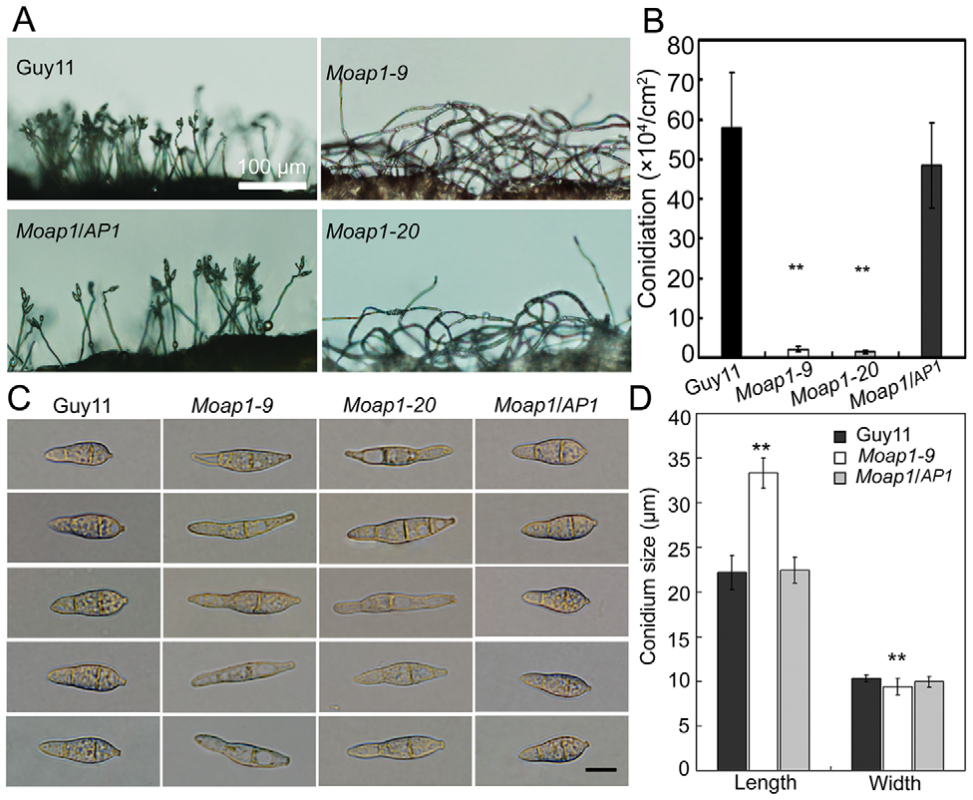
*MoAP1* disruption leads to abnormal conidial morphology and reduced conidiation. (A) Development of conidia on conidiophores is affected by *Moap1* deletion. Strains grown on RDC medium for 7 days were examined by light microscopy. Bars equal 100 µm. (B) Statistical analysis of conidia production. The conidia produced by the wild type strain (Guy11), the mutants and complemented strains grown on RDC medium for 10 days were collected, counted, and analyzed by Duncan analysis (p<0.01). Asterisks indicate significant differences among Guy11, the *Moap1* mutants and complemented strains. Error bar represents standard deviation. (C) Conidium morphology. Conidia were harvested from RDC medium, diluted to 1.0×10^5^ spore/ml, and observed by light microscopy. Bars equal 10 µm. (D) Conidia size comparison. The conidia sizes were determined as width by length from 150 conidia of each strain. Asterisks indicate that the difference is statistically significant. Error bars represent standard deviations.

Conidia, which are borne on specialized stalks called conidiophores, play an important role in the disease cycle of rice blast [42]. Given that the *Moap1* mutant showed reduced hyphal growth, we investigated the role of MoAP1 in conidia formation. Conidiation of the wild-type strain (Guy11), *Moap1* mutants (*Moap1-9* and *Moap1-20*), and the complement strain (*Moap1*/*AP1*) was determined in 10 day old RDC cultures [43]. The most striking finding was that conidiation was dramatically reduced by approximately 30 to 40-fold in *Moap1* deletion mutants (*Moap1-9*, 30-fold; *Moap1-20*, 40-fold), compared with the wild-type and complement strains (Figure 2A and 2B). Of the few conidia that formed in *Moap1-9* and *Moap1-20*, most exhibited abnormal, elongated, and spindly morphology (Figure 2C and 2D). Combined with the expression profiles of *MoAP1* by qRT-PCR that showed a much higher level of *MoAP1* expression in conidia (see Figure S3A), we concluded that MoAP1 plays an important role in conidial formation.

### *MoAP1* disruption leads to hypersensitivity to the oxidative stress

**Figure 3:**
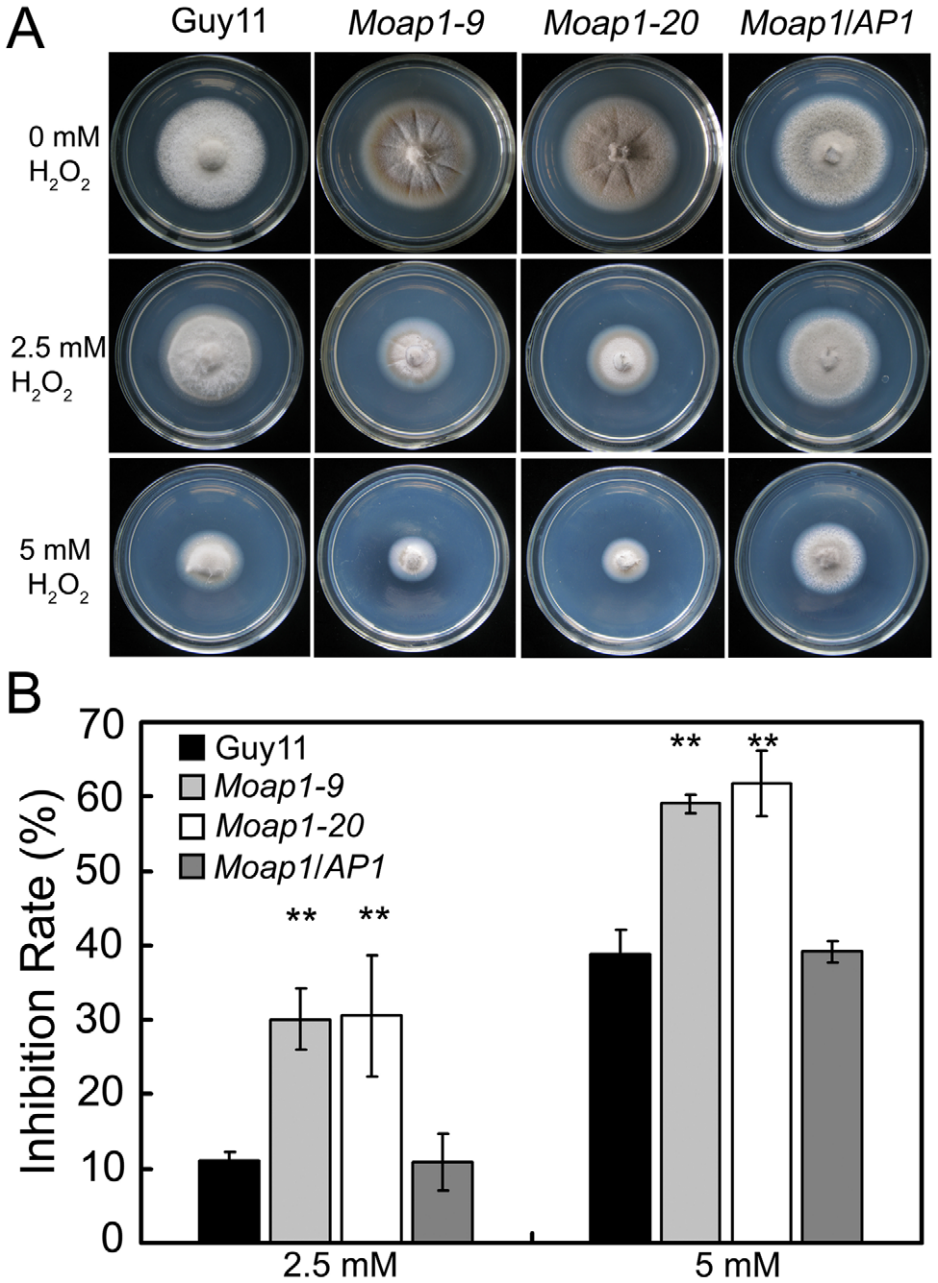
The *Moap1* deletion mutants are hypersensitive to H_2_O_2_. (A) Mycelia growth of the *Moap1* mutants under oxidative stress. The wild type strain Guy11, *Moap1* mutants and the complemented strain were inoculated on CM medium with or without 2.5 or 5 mM H_2_O_2_ and cultured at 28°C for 5 days. (B) The colony diameters of the testing strains were measured and subjected to statistical analysis. The growth inhibition rate is relative to the growth rate of each untreated control [Inhibition rate  =  (the diameter of untreated strain - the diameter of treated strain)/(the diameter of untreated strain ×100%)]. Three repeats were performed and similar results obtained. Error bars represent the standard deviations and asterisks represent significant differences (p<0.01).

*S. cerevisiae* Yap1 was involved in the oxidative stress response [21], [22]. To investigate whether MoAP1 exhibits the same function, the wide-type, *Moap1* mutants, and the complemented (*Moap1*/*AP1*) strains were exposed to H_2_O_2_. The mycelia growth of the *Moap1* mutants was apparently affected (Figure 3A and 3B). Exposure to 2.5 and 5 mM H_2_O_2_, respectively, led to an average 19% (2.5 mM) or 22% (5 mM) greater growth inhibition rate than the wild-type strain (Figure 3B). The involvement of the *MoAP1* gene in H_2_O_2_ resistance was confirmed by genetic complementation in which the complemented strain (*Moap1/AP1*) was as resistant to H_2_O_2_ as the wild-type strain (Figure 3A and 3B).

### *MoAP1* disruption led to excess oxidative bursts during conidiation and germination

**Figure 4:**
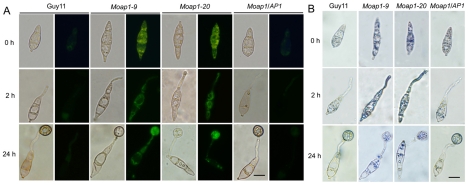
The ROS accumulation is compromised in the *Moap1* mutant during infection. (A) Detection of the superoxide by dihydrorhodamine 123 staining. Conidia were inoculated on coverslips and incubated in a moist chamber at 28°C for 0, 2 and 24 hrs before being stained for 2 hrs, rinsed twice with PBS and viewed by epifluorescence microscopy. Fluorescence images were captured using a 100-ms exposure for absorbed light using a GFP filter. Representative bright-field images at each time point are shown. (Scale bars  = 10 µm). (B) Detection of the superoxide by NBT staining. Conidia were prepared as above, stained with a 0.3 mM NBT aqueous solution for 1 hr and viewed by light microscopy. Multiple observations were made and the representative figures were presented (Scale bars  = 10 µm).

In fission yeast *Schizosaccharomyces pombe*, the transcription factor Pap1, together with another transcription factor SpAtf1, regulates the genes involved in ROS homeostasis and the response to the extrinsic oxidative stress [44], [45]. Intracellular ROS is known to have multiple functions in fungal pathogenicity [14], [15], [30], [32], [46]. In *M. oryzae*, intracellular ROS is the key to its virulence in rice seedlings, and the NADPH oxidase mutants lost virulence on the susceptible rice cultivar CO-39 because of their obstructed ROS production [46]. Thus, visualization of ROS accumulation was performed to investigate ROS metabolism during conidiation and germination in *Moap1* mutants. We first investigated the production of ROS using dihydrorhodamine 123, which exhibits green fluorescence during reduction by superoxide radicals. Using this technique, it appeared that the *Moap1* conidium accumulated higher amounts of superoxide than the wild-type (Figure 4A). Such increased accumulation of superoxide was also detected in the *Moap1* mutants during conidia germination. Green fluorescence was typically more intense in the germ tubes and mature appressoria of the *Moap1* mutants than the wild-type strain or the complemented *Moap1/AP1* strain (Figure 4A). To further confirm enhanced accumulation of ROS in the *Moap1* mutants, another kind of reactive oxygen species detection probe, nitroblue tetrazolium (NBT), which forms a dark-blue water-insoluble formazan precipitate on reduction by superoxide radicals, was used. Using this procedure, we found that the *Moap1* mutants accumulated higher amounts of superoxide, with more intense formazan precipitates in the germ tubes and mature appressoria (Figure 4B). In contrast, the appressoria and infection hyphae of the wild type strain had less formazan precipitates than the *Moap1* mutants (Figure 4B). Thus, both staining results indicated that *MoAP1* disruption leads to excess oxidative bursts in conidiation and germination.

### Subcellular localization of MoAP1 is modulated by H_2_O_2_

AP1 proteins are translocated from the cytoplasm to the nucleus following the oxidative stress [14], [15], [27], [30]. To analyze if this is also true for MoAP1, we generated a C-terminal *MoAP1::eGFP* fusion gene under the drive of the *TrpC* promoter and introduced the fusion gene into the wild-type strain. Conidia of the *MoAP1::eGFP* containing transformants were harvested and observed under a fluorescence microscope. In the absence of the oxidative stress, the MoAP1::eGFP fusion protein was distributed throughout the cell and was apparently excluded from nuclei (see Figure S5). In contrast, fluorescence was concentrated in a single spot after exposure to 2 mM H_2_O_2_ for 2 hrs, as shown by the colocalization of the eGFP and DAPI fluorescence signals (see Figure S5), indicating the nuclear localization of MoAP1::eGFP in response to the oxidative stress.

### MoAP1 is required for invasive hyphae growth and pathogenicity

**Figure 5:**
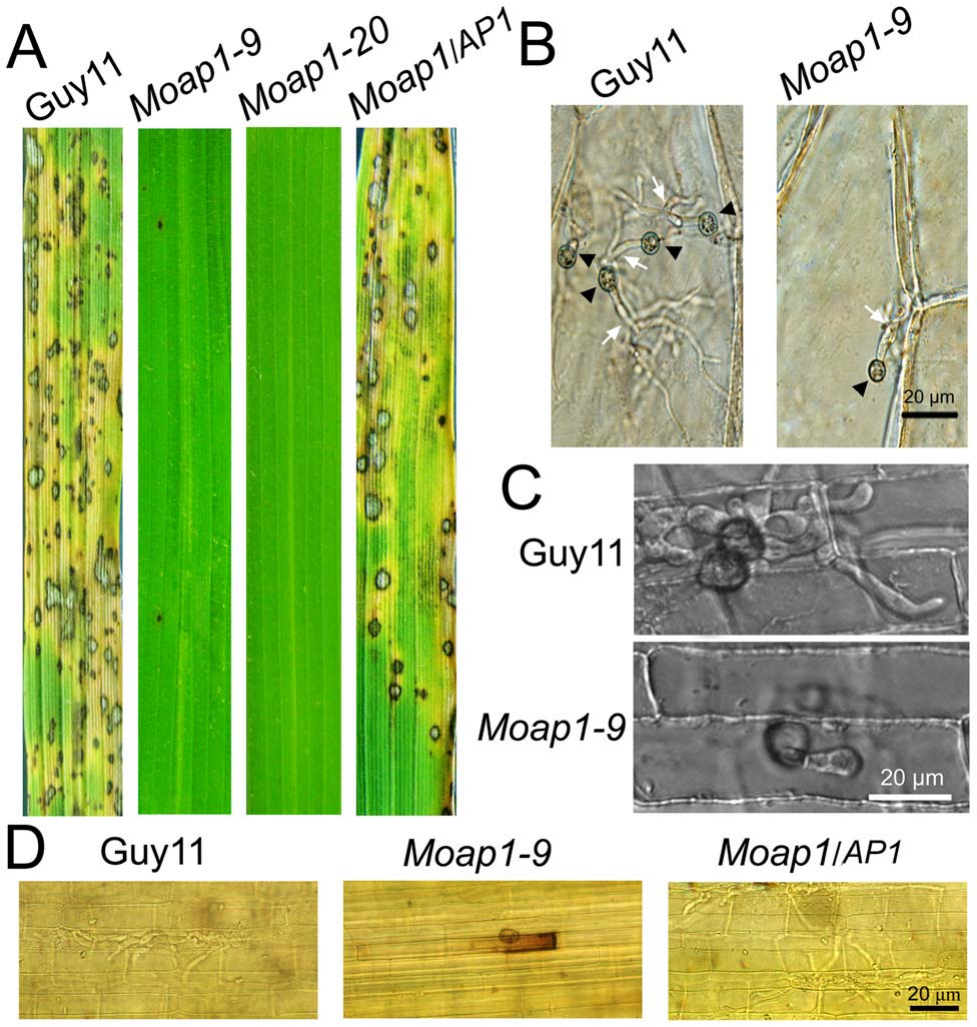
Pathogenicity of the *Moap1* mutant strain. (A) The *Moap1* deletion mutants lost pathogenicity on rice leaves. 4 ml of conidial suspension (1×10^5^ conidia/ml) for each strain was sprayed on 4-week-old rice seedlings (*O. sativa* cv CO-39) and 60 healthy rice plants were used in each independent experiment. Diseased leaves were harvested 7 days after inoculation. (B) Onion epidermis cell penetration assay of the *Moap1* mutant. The assay was performed by inoculating 30 µl conidia suspension obtained from Guy11, *Moap1-9* and the complemented strain. Light microscopic image examination was performed and recorded. Arrows indicate appressoria or invasive hyphae inside cells. (Scale bars  = 20 µm). (C) Rice leaf sheath penetration assay indicating severely confined growth of the *Moap1* mutant hyphae at 48 hpi. (Scale bars  = 20 µm). (D) DAB staining indicated the ROS accumulation at the infection site on the rice leaf sheath by the *Moap1* mutant at 48 hpi but not by the wild type and complemented strains. (Scale bars  = 20 µm).

To determine whether MoAP1 was involved in pathogenicity, conidial suspensions (1×10^5^ conidia/ml) of both the *Moap1* mutant and wild-type strains were sprayed onto 4-week-old rice seedlings (*Oryza sativa* cv CO-39). At 5 days after inoculation, symptoms had fully emerged on rice leaves inoculated with the wide-type strain, but no lesion on the *Moap1* mutant inoculated rice leaves (see Figure 8). When observation was made at 7 days post infection, there were still no typical lesions developed on *Moap1* mutant infected leaves. Only small necrotic-like dark brown spots were observed occasionally (Figure 5A). The loss of pathogenicity was complemented by reintroducing the *MoAP1* gene into the *Moap1* mutant (Figure 5A). Similarly, pathogenicity test with conidia suspension or mycelial plug on barley leaves displayed very similar result (see Figure S6A and S6A).

To investigate possible reasons for the lost pathogenicity, an onion epidermis penetration assay was performed. At 48 hours post-inoculation, both the wild-type and the *Moap1* mutant strains could penetrate the onion epidermis cell, but the invasive hyphae of the wild type strain freely expanded into the onion epidermis cells, in contrast to the restricted growth of the *Moap1* strain (Figure 5B). The deficiency of hyphae expansion could be complemented by reintroduction of the *MoAP1* gene (Figure 5B). Further assay using the rice leaf sheath generated the similar results (Figure 5C). Moreover, DAB staining showed the accumulation of ROS at the infection site of the *Moap1* mutant, but not the wild type and complemented strains (Figure 5D). These results indicated that MoAP1 is required for invasive hyphae growth and the defect in invasive hyphae might be responsible, at least in part, for the loss of pathogenicity.

### *MoAP1* disruption attenuates the activity of extracellular peroxidases and laccases

**Figure 6:**
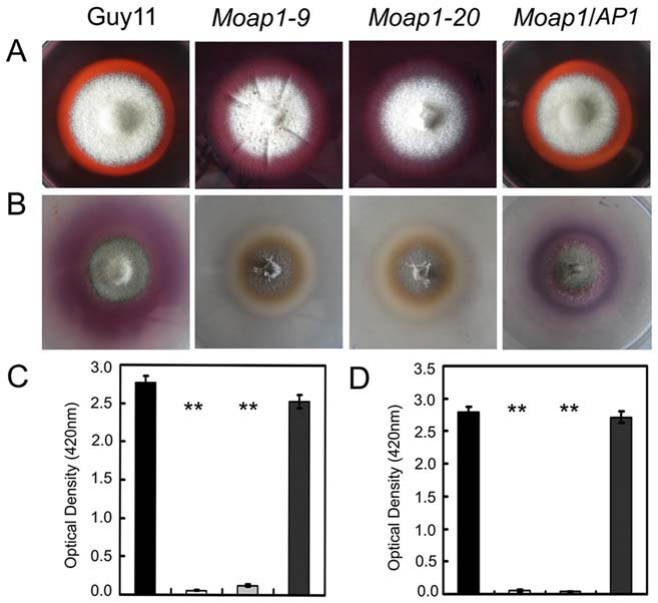
Decreased extracellular laccase and peroxidase activities in the *Moap1* mutants. (A) Strains of Guy11, *Moap1* mutants and complemented strain were inoculated on CM medium containing Congo Red dye at a final concentration of 200 µg/ml. The discoloration of Congo Red was observed after incubation for 5 days. (B) The laccase activity was monitored in complete media supplemented with 0.2 mM ABTS after 3 days of incubation. (C and D) Guy11, *Moap1* mutants and complemented strains were inoculated in CM liquid medium and the laccase activity (C) and the peroxidase activity (D) were measured in the filtrate cultures through ABTS oxidization test with or without H_2_O_2_. Dark column indicates Guy11, both white and light grey column equal indicates the *Moap1* mutant, and dark gray column indicates the *Moap1/AP1* complement strain. Error bars represent the standard deviations and asterisks represent significant differences among the strains tested (p<0.01).

In *M. oryzae*, defects in cell wall composition can influence appressorium formation and impair successful infection of rice host [47], [48], [49], [50]. Congo Red (CR), which binds to cell wall component β-1,4-glucan [51], is commonly used to detect cell wall integrity. To determine whether MoAP1 has a role in cell wall integrity, CR was added to CM medium (200 µg/ml). No difference was found in the growth of mycelium between the *Moap1* mutant (15% inhibition) and the wild-type strain (16% inhibition). However, the degradation halo of CR by the *Moap1* mutant was not as apparent as the wild type (Figure 6A). This indicated a deficiency of the CR-degrading activity in the *Moap1* mutant. An enzyme activity assay using culture filtrates further indicated that the *Moap1* mutant nearly lost all of the peroxidase activity (Figure 6D). Moreover, we determined the activity of additional extracellular enzyme laccases in either solid or liquid CM medium, and found that, in each case, the decreased laccase activity was observed in the *Moap1* mutant, with less oxidized dark purple stain around its colony and lower levels of laccase activity in the culture filtrate, compared with the wild-type strain (Figure 6B and 6C). These data suggest that *MoAP1* disruption resulted in a decreased peroxidase and laccase activities.

### Addition of copper sulfate to the *Moap1* mutant restores laccase activity and complements pigmentation

We observed altered pigmentation of the *Moap1* mutants on CM medium, with a yellowish-brown color to the mutant strains compared with a dark pigmentation of the wild-type strain (Figure 1D). It is well known that copper sulfate can stimulate the biosynthesis of melanin by inducing the laccase activity [52], [53]. When copper sulfate was added to CM medium at 1 mM, which did not affect mycelial growth of either the *Moap1* mutants or the wild-type strain, the laccase activity was restored to the *Moap1* mutants, as indicated by dark pigmentation (see Figure S7). Combined with the accumulation of reactive oxygen species in conidia and mycelia, we hypothesized that the *Moap1* mutant was likely in a hyperoxidative state, which may, in turn, lead to reduced oxidative cross-linking of melanin, and which could be compensated for by an increase in the laccase activity.

### Differential expression of pathogenicity-associated genes revealed genes linked to MoAP1

**Figure 7:**
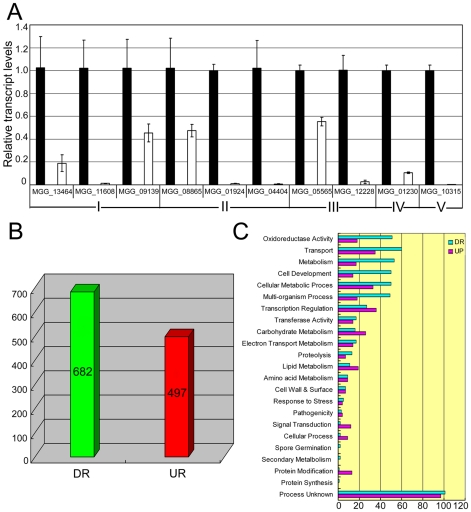
Differential gene expression analysis on transcriptomes of the *Moap1* mutant and Guy11 strains. (A) Real time RT-PCR validated the RNA-SEQ results. Real time RT-PCR was carried out to confirm the RNA-SEQ results through random selection of genes that were down-regulated in the *Moap1* mutant. Laccase genes (I), extracellular peroxidase gene (II), and genes involved in the redox homeostasis (III) were down-regulated in the *Moap1* mutant. IV and V indicated *MGG_01230* (*Mossadh*) and *MGG_10315* (*MoMpg1*) that were down-regulated. (B) Numbers of altered genes expression in *Moap1* mutants. Gene expression profiles were analyzed and 682 genes were down-regulated while 497 genes were upregulated in *Moap1* mutants in comparison to the wild type strain (Guy11). Genes whose expression were up or down as indicated by expression profiling were chosen based on the log2 ratio (*Moap1*/Guy11) values that were either 1.5- fold more or - less. DR and UR denote down- and up-regulation. (C) Functional grouping of genes up- or down-regulated in *Moap1* mutants. The up-regulated (in purple) and the down-regulated (in cyan) genes were divided into 23 groups according to their putative functions as described in Materials and Methods.

To understand reasons for phenotypic changes and the loss of pathogenicity in the *Moap1* mutants, we generated serial analysis of gene expression (SAGE) libraries for the wild-type strain (Guy11, 4,924,107 tags) and the *Moap1* mutant (4,690,301 tags) using mycelia grown in liquid CM medium. To confirm gene expression patterns derived from the SAGE libraries, 10 down-regulated genes in the *Moap1* mutant were randomly selected and validated by qRT-PCR. The results showed that each gene expression pattern was consistent with that in the SAGE data (Figure 7A and Table S1), despite the discrepancy of the fold-change being higher in qRT-PCR than in SAGE data.

To identify genes that were subjected to regulation by MoAP1, we compared the gene expression profiles between the wild-type strain and the *Moap1* mutant. In total, 497 genes were up-regulated and 682 genes were down-regulated (Figure 7B). These genes were functionally grouped into GO categories based on manual curation, as described in Materials and Methods. We found that 69 genes related to redox-homeostasis were altered in expression and 5/6 of those genes, either with the signal peptide or not, were down-regulated in the *Moap1* mutant (Figure 7C). We also noted a significant decrease in the expression of genes involved in transcriptional regulation, protein degradation, lipid metabolism, secondary metabolism, cellular transportation, and cell development (Figure 7C).

Since the *Moap1* mutants were non-pathogenic due to the confinement of its invasive hyphae within the originally penetrated onion skin cells and rice leaf sheath cells, we examined the putative target genes of MoAP1 based on their previously defined roles and identified seven genes that were previously characterized to regulate virulence (Table 1). Because of the severely decreased conidiation of the *Moap1* mutants, we screened the SAGE database to identify genes that were involved in conidiation. A previously defined gene *MoCOS1* (*MGG_03977*) involved in conidiophore stalk formation [54] and a novel vitamin B6 synthesis amidotransferase-encoding gene (*MGG_05981*) [55] were found to be also significantly down-regulated in the *Moap1* mutant (see Table 1 and Figure S8A). Additionally, three laccase-encoding genes (*MGG_13464*, *MGG_11608*, and *MGG_09139*) were also found to be severely down-regulated (see Table 1 and Figure S8B).

**Table 1:** SAGE data for genes encoding known pathogenicity factors or pathogenicity-associated functions.

Gene_ID	Description	ELSD(a) (*Moap1*/WT)	p-Value	Reference	Putative MoAP1 binding sites (b)
MGG_10315	Hydrophobin (Mpg1)	−9.72	0	[89]	Yes
MGG_07536	Metalloprotease/Zn amino-peptidase	−2	5.36E-05	[55]	No
MGG_09312	Transcription factor Zn2Cys6	−1.781	4.46E-45	[55]	No
MGG_00111	P-type ATPase (Pde1)	−1.58	0.00043	[102]	Yes
MGG_05344	Snodprot1 homolog (Msp1)	−1.39	0	[103]	No
MGG_06424	PEX3-peroxisome	−1	0.01	[55]	No
MGG_09898	Adenylate cyclase (Mac)	−1	0.027	[104]	No
MGG_03977	Putative Zinc-Finger Protein	−4.52	3.29E-48	[54]	No
MGG_05981	vitamin B6 synthesis amidotransferase	−2.70	2.74E-243	[55]	Yes
MGG_13464	laccase	−7.61	0	Unpublished	Yes
MGG_11608	laccase-2	−3.40	4.20E-159	Unpublished	No
MGG_09139	laccase-1	−2.32	1.44E-13	Unpublished	Yes

(a) ELSD indicated expression level in SAGE database.

(b) ‘Yes’ indicating at least one putative MoAP1 binding site was identified in the promoter region of the gene while ‘No’ stand for non putative MoAP1 binding site was identified in the promoter region.

### MoAP1 regulates the expression of four genes involved in pathogenicity

**Figure 8:**
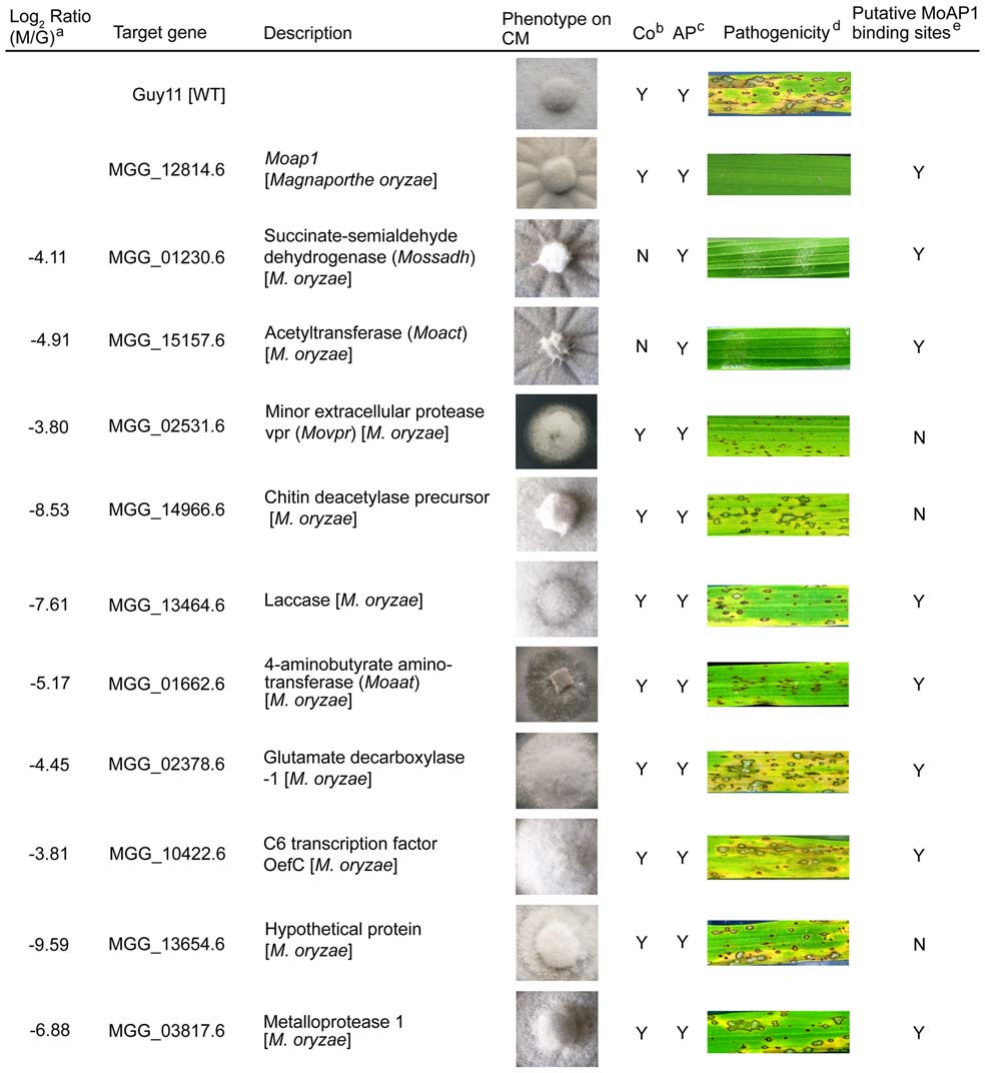
Phenotype observation and pathogenicity assay of targeted gene deletion mutants. Ten down-regulated genes in the *Moap1* mutant were selected for gene deletion and the phenotype of each mutant was compared and displayed. Gene ID numbers were sourced from www.broad.mit.edu/annotation/genome/magnaporthe_grisea/. ^a^ Log_2_ ratio (*Moap1*/Guy11) value stands for gene expression fold differences in the *Moap1* mutants. ^b^ Co represents conidiation and ^c^ AP represents appressorium. ^d^ The diseased leaves were harvested at 5 dpi, and the results at 7 dpi displayed in Figure S12. ^e^ The letter Y stands for yes and N stands for no.

Among MoAP1 regulated genes, we selected 10 whose expression was severely down regulated, and of which 7 contained the putative AP1 binding sites (Figure 8, Table 2 and Table S1), and characterized their functions by generating individual gene disruption mutants. Among the deletion mutants, we compared the phenotypes in mycelial growth, conidiation, appressorium formation, and pathogenicity on rice, and found that disruption of the transcription factor MoOefC (*MGG_10422*), the hypothetical protein-encoding gene (*MGG_13654*), the laccase protein-encoding gene (*MGG_13464*), the glutamate decarboxylase 1-encoding gene (*MGG_02378*), the metalloproteinase 1-encoding gene (*MGG_03817*), and the chitin deacetylase precursor-encoding gene (*MGG_14966*) did not alter the above-mentioned morphological phenotypes or pathogenicity (Figure 8). However, the disruption of the minor extracellular protease-encoding gene (*MGG_02531*, *MoVPR*) or the 4-aminobutyrate aminotransferase-encoding gene (*MGG_01662*, *MoAAT*) led to attenuated virulence on the rice cultivar CO-39. The *Movpr* mutants also had an abnormal morphology on CM medium and retarded mycelial growth (Figure 8). Furthermore, two mutants containing deletion of alleles encoding succinate-semialdehyde dehydrogenase (*MGG_01230*, *MoSSADH*) and acetyltransferase (*MGG_15157*, *MoACT*), were identified as losing the ability to generate aerial hyphae and conidia, and inability to cause infection. All these findings were summarized in Figure 8. Finally, we also found that both *Mossadh* and *Moact* mutant strains displayed no lesions on barley leaves infected with mycelial plugs 7 days post inoculation (see Figure S6C).

**Table 2:** AP1 binding motif in the promoter region of the down-regulated genes.

Gene_ID	Description	ELSD(a) (*Moap1*/WT)	p-Value	Putative MoAP1 binding sequence (b)
MGG_12814.6	Conserved hypothetical protein (MoAP1)		1e-75	GCTTACTTC
MGG_01230.6	Succinate-semialdehyde dehydrogenase (MoSsadh)	−4.11	0.0	GTACTAA, TGAGTAT, AGACTAA
MGG_15157.6	Acetyltransferase (MoAct)	−4.91	3e-20	ATACTAA, TTACTCA
MGG_13464.6	Laccase	−7.61	0.0	CAAGTCAGC
MGG_01662.6	4-aminobutyrate aminotransferase	−5.17	0.0	ACTGACTAG, TTAGTAAAG, TTACTCC
MGG_02378.6	Glutamate decarboxylase 1	−4.45	0.0	TGAGTAA, TGACTAT
MGG_10422.6	C6 transcription factor OefC	−3.81	8e-54	TTAGTAAGT
MGG_03817.6	Metalloprotease	−6.88	4e-26	AGAGTAA

(a) ELSD indicated expression level in SAGE database.

(b) Sequences in the table indicating the putative MoAP1 binding site that identified in the promoter region of the gene.

### MoSsadh and MoAct are required for appressorium-like structure-mediated penetration

**Figure 9:**
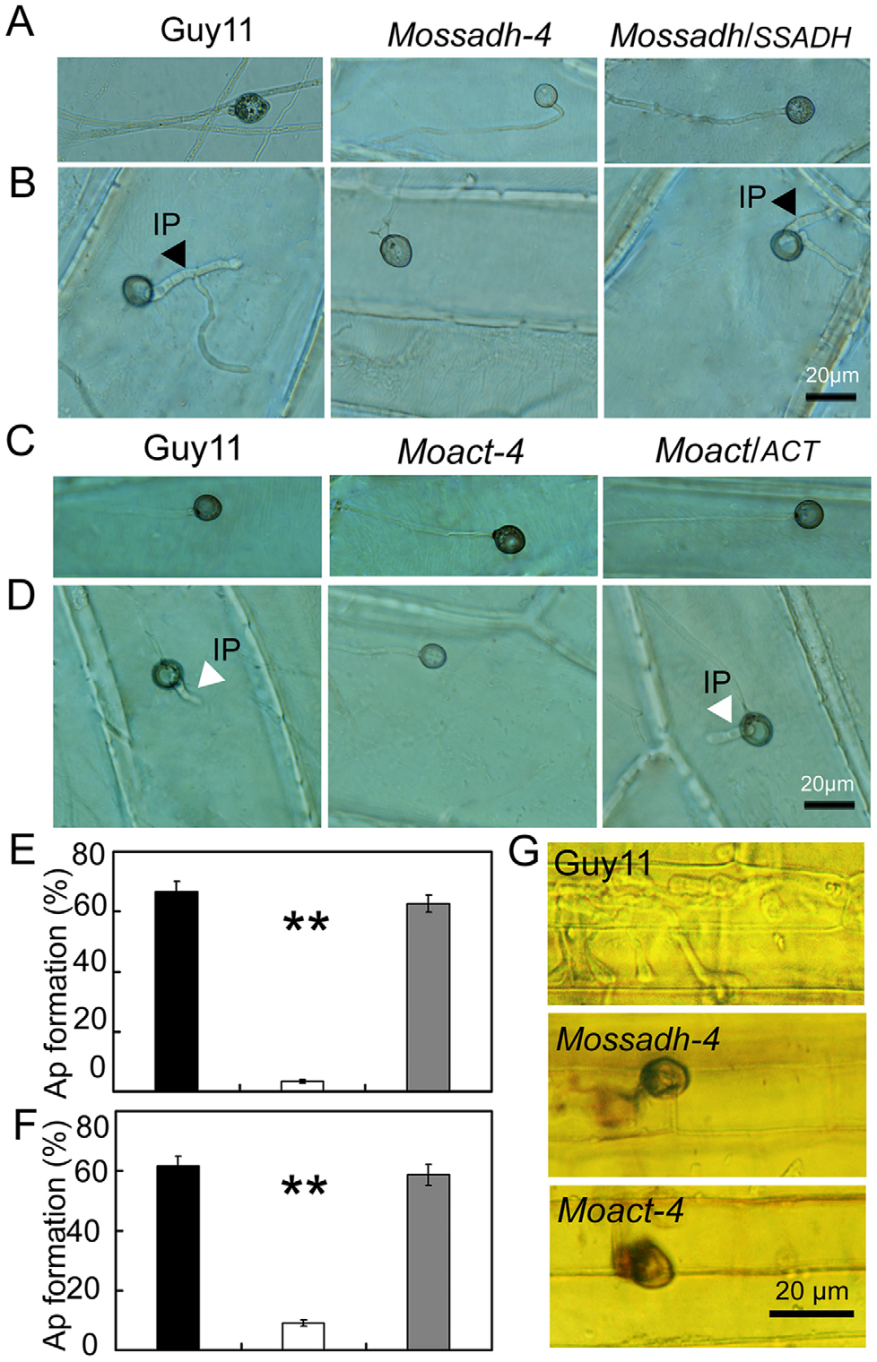
The appressorium-like structure is unable to penetrate the onion epidermis or rice leaf sheath cells. (A and C) The appressorium-like structure develops at the hyphal tips. The appressorium-like structure was induced by placing the mycelia blocks on the onion epidermis cell for 24 hrs. (Scale bars equal 20 µm). (B and D) Onion epidermis cell penetration assay. The hyphae blocks were inoculated on the onion skin cells for 48 hrs. Penetration was observed using DIC microscopy. (Scale bars equal 20 µm). (E and F) Statistical analysis of appressorium-like structure development. A total of 300 hyphal tips were examined and counted. Dark column indicates Guy11, white column indicates the *Mossadh* or *Moact* mutant, and gray column indicates the *Mossadh/SSADH* or *Moact*/*ACT* complement strain. Error bar represents standard deviations and asterisks indicate that the differences were significant. (G) Rice leaf sheath penetration assay. Appressorium like structure formed by the *Mossadh* mutant and *Moact* mutant are incapable of penetration into the rice leaf sheath cells 48 hrs post injection (Scale bars  = 20 µm).

Generally, *M. oryzae* infects rice aerial organs, such as leaves and stems, through appressoria, which develops from conidia. However, hyphae can also invade rice roots [56] and wounded leaf tissue [57]. Previous studies suggested that the hyphae tips can also form an appressorium-like structure to break the rice leaf cuticle and cause disease [54], [58]. As both the *Mossadh* and *Moact* mutants lost pathogenicity on rice leaves, we hypothesized that the lost pathogenicity might be due to the lack of the ability to breach the rice leaf cuticle. Given that successful lesion development requires the development of appressoria, we examined appressorium formation on the onion skin using microscopy and found that both the wild-type and the mutants developed appressorium-like structures at the hyphal tips (Figure 9A and 9C). However, the appressorium-like structures formed by the *Mossadh* (Figure 9E) and *Moact* mutants (Figure 9F) were significantly fewer than that formed by the wild-type strain (34%). Furthermore, those appressorium-like structures could not penetrate the onion skin cells, compared with the easy penetration of the cells by the wild-type strain (Figure 9B and 9D). The penetration assay using the rice leaf sheath generated similar results (Figure 9G). These results indicate that both MoSsadh and MoAct are required for appressorium-like structure formation, as well as penetration.

### MoSsadh and MoAct are equally required for invasive hyphae growth

**Figure 10:**
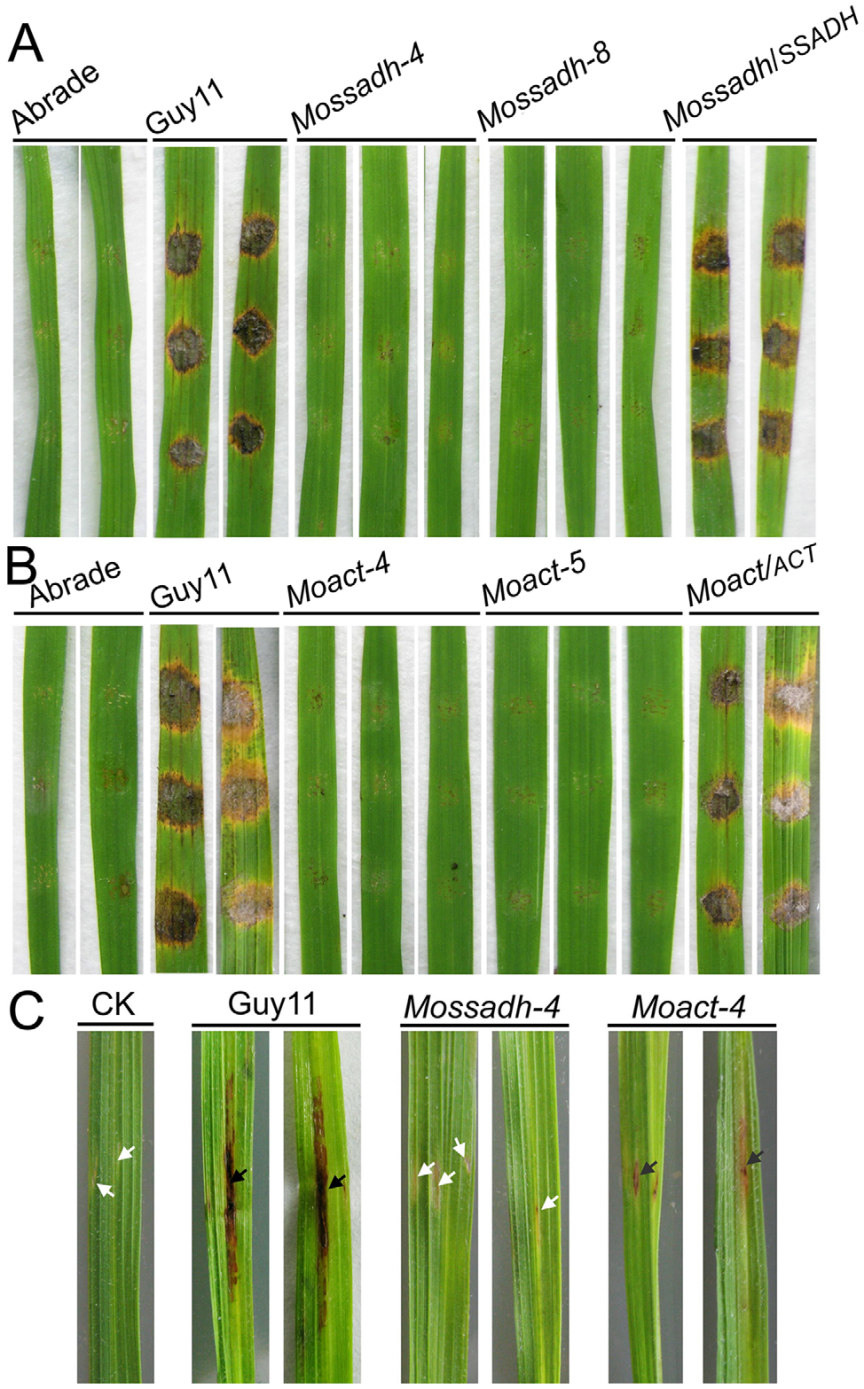
Pathogenicity test of *Mossadh* and *Moact* mutant strains on the wounded rice leaves. (A) Pathogenicity test of *Mossadh* mutants on the wounded rice leaves. The mycelia blocks of the wild type strain Guy11, *Mossadh* mutants, and the complemented strain were inoculated on the wounded rice leaves and then cultured under moist conditions with 28°C for 5 days. The wounded rice leaves with the CM agar plugs on was used as negative control. This experiment was performed three times with 10 pieces of rice leaves for each strain. Similar results were obtained in each test and this picture showed the representative result. (B) Pathogenicity test of *Moact* mutants on the wounded rice leaves. The mycelia blocks of Guy11, *Moact* mutants and the complemented strain were inoculated on the wounded rice leaves and observations made as above. (C) Pathogenicity test of the mutant strain by injection of hyphae fragments. The hyphae fragments of Guy11, the mutant strains and respective complemented strains were prepared as described in Materials and Methods. From left to right, leaves injected with water, wild type strain, *Mossadh* mutant, and *Moact* mutant. The arrowheads in black indicate injection sites with necrosis, while the arrowheads in white indicate injection sites without necrosis.

The results above indicated that *Mossadh* and *Moact* mutants lost the ability to penetrate the plant cell. If these results are the main reason for the lost pathogenicity, given the conditions, abraded rice leaves should restore pathogenicity to the mutants. To examine this, we inoculated wounded rice leaves with agar plugs containing mycelial tips to evaluate pathogenicity. Both the *Mossadh* and *Moact* mutants were unable to cause symptoms, while the wild-type strain produced visible diffuse lesions on rice leaves 5 days after inoculation (Figure 10A and 10B). The similar result was found when the observation was made at 7 days post infection (dpi) (see Figure S9B).

To further investigate the loss of pathogenicity, we injected the mycelial fragments into rice leaves using a syringe. The wild-type strain caused typical necrotic symptoms along the injection site and formed developmental lesions at 5 dpi (Figure 10C). However, the *Mossadh* mutant caused no necrotic symptoms, while the *Moact* mutant caused severely restricted necrosis (Figure 10C). These results suggested that the loss of pathogenicity in *Mossadh* and *Moact* mutants is not only due to failure in penetration, but also to the lost ability of forming invasive hyphae. Similar results were observed at 7 dpi (see Figure S9A).

### *Mossadh* and *Moact* mutants are both hypersensitive to H_2_O_2_

**Figure 11:**
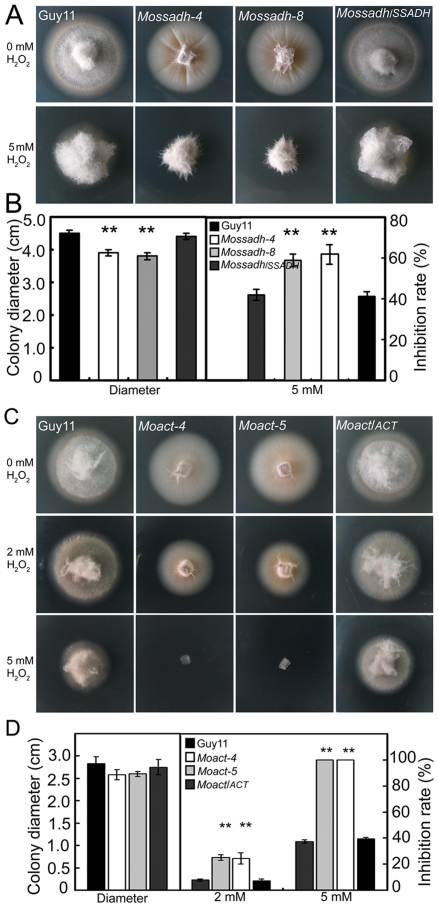
*Mossadh* and *Moact* are hypersensitive to H_2_O_2_. (A) Phenotype of the *Mossadh* mutants under the oxidative stress. The wild type strain, *Mossadh* mutants and the complemented strain were inoculated on CM agar medium with or without 5 mM H_2_O_2_ and cultured at 28°C for 5 days. (B) Statistical analysis of mycelia growth rate with or without H_2_O_2_. The analysis was similar to Figure 3B. Error bars represent the standard deviations, and asterisks represent significant differences among Guy11, *Mossadh* mutants and the complemented strain (p<0.01). (C) Phenotype of the *Moact* mutant strains under the oxidative stress. Guy11, *Moact* mutants, and the complemented strain were inoculated on CM agar medium with or without 2 to 5 mM H_2_O_2_ and cultured at 28°C for 3 days. (D) Statistical analysis of mycelia growth. Error bars represent the standard deviations, and asterisks indicate that the differences among Guy11, *Moact* mutants and the complemented strain were statistically significant (p<0.01).

The stress-tolerance mechanisms of plant pathogens play an important role in virulence [14], [15], [59], [60]. To assess whether the putative MoAP1 targets MoSsadh and MoAct play an active role in the tolerance to exogenous H_2_O_2_, the mutant strains were inoculated on H_2_O_2_-containing CM medium. The assay results showed that both type of mutants were more sensitive to H_2_O_2_ than the wild-type strain (Figure 11A and 11C). The mycelial growth of the *Mossadh* mutant was severely inhibited on CM medium containing 5 mM H_2_O_2_, with 20% (*Mossadh-4*) and 17% (*Mossadh-8*) greater inhibition rates than the wild-type strain (Figure 11B). Meanwhile, the *Moact* mutants displayed higher sensitivity to H_2_O_2_ than either the *Moap1* mutants or the *Mossadh* mutants, with an average 18% greater mycelial growth inhibition rate than the wild-type strain at 2 mM H_2_O_2_ and 61% at 5 mM H_2_O_2_. The sensitivity of the mutants to H_2_O_2_ was complemented by reintrodution of the respective wild type genes (Figure 11D).

### *Mossadh* and *Moact* mutants displayed similar morphological phenotypes to *Moap1* mutants

**Figure 12:**
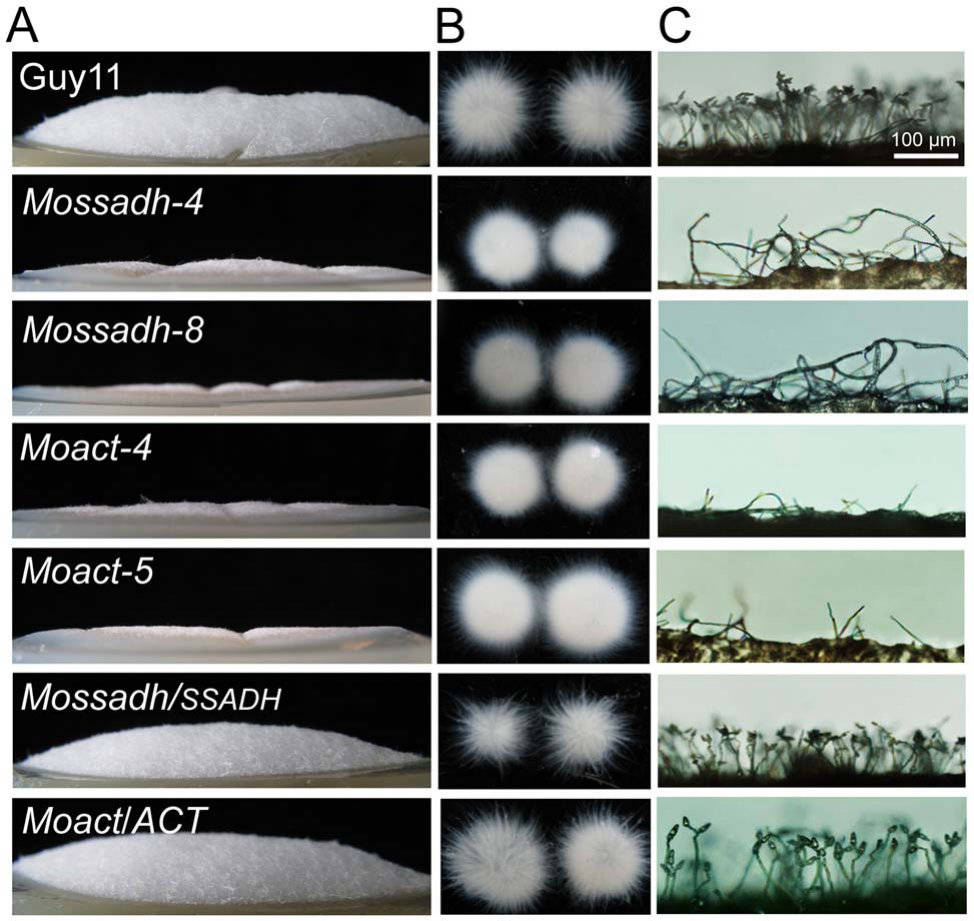
*Mossadh* and *Moact* mutants displayed phenotypes similar to the *Moap1* mutants. (A) Aerial hyphae growth reduction in *Mossadh* and *Moact* mutants. The wild type, *Mossadh* mutants, *Moact* mutants, and respective complemented strains were inoculated on CM medium and cultured at 28°C for 5 days. The aerial hypha was photographed. (B) The phenotype of mycelia grown in liquid CM medium. Strains tested were grown and observed as described in Figure 1C. (C) Development of conidia on conidiophores. Light microscopic observation was performed on strains grown on RDC medium for 7 days. Bars  = 100 µm.

To further investigate the roles of MoSsadh and MoAct on hyphal growth, we compared their growth on CM and RDC medium. The *Mossadh* and *Moact* mutants exhibited reduced growth (see Figure S10), and the aerial hyphae was also sparser and thinner, compared with Guy11 (Figure 12A). When observed by light microscopy, no conidia were found (Figure 12C). The colonies of the two mutants were also smooth appearing and displayed 5-10 radial folds in each colony, similar to the *Moap1* mutants (Figure 8 and 12A). To further understand possible reasons for this, we grew the *Moap1* mutants in liquid CM medium and found that it could form compact mycelia mat, in contrast to the sparse one formed by the wild-type and the complemented strain (Figure 12B). Meanwhile, the hyphal branching patterns of Guy11, the *Mossadh* mutants, the *Moact* mutants, and the complemented strains were examined using CFW staining, which revealed that hyphal branching was severely reduced in the *Mossadh* and *Moact* mutants (see Figure S4B and S4C). Moreover, the colonies of the *Mossadh* and *Moact* mutants were less pigmented than the wild-type and complemented strains (see Figure S10A and S10B). Combined with the results of the *Moap1* mutants, these results further indicated that MoAP1 controls the morphological phenotypes of *M. oryzae* by regulating genes including *MoSSADH* and *MoACT*.

### Disruption of *MoSSADH* and *MoACT* attenuated peroxidase and laccase activities

To functionally analyze possible reasons for attenuated virulence of both the *Mossadh* and *Moact* mutants in rice, we examined the cell wall integrity, which is regarded as the most important factor affecting appressorium formation in *M. oryzae* [47], [48], [49], [50]. By the addition of CR to CM medium at 200 µg/ml, the mycelial growth of both the *Mossadh* mutants (average 17% inhibition rate) and *Moact* mutants (average 16% inhibition rate) was similar to that of the wild-type strain (average 16% inhibition rate). However, no degradation halo of CR on either the *Mossadh* or *Moact* mutant plates was present in comparison to the wild type and complemented strains, which displayed a visible degradation halo of CR (see Figure S11A). To test for secreted peroxidases, culture filtrates of the *Mossadh* mutant, *Moact* mutant, wild-type Guy11, and complemented strains were collected and assayed for secretion of extracellular peroxidases. The results showed that both the *Mossadh* and *Moact* mutants were deficient in peroxidase activities (see Figure S11C and S11E). For the altered pigmentation in the *Mossadh* and *Moact* mutants, we again compared the laccase activity, hypothesizing that the polyphenol oxidase function may be impaired in both these mutants. The oxidation of the laccase substrate 2,2′-azino-di-3-ethylbenzthiazoline- 6-sulfonate (ABTS) showed that the laccase activity was reduced in both the *Mossadh* and *Moact* mutants. The oxidized dark-purple stain around the colonies of the *Mossadh* and *Moact* mutants was less than that of Guy11 (see Figure S11B). Similarly, the laccase activity in the filtrates of the *Mossadh* and *Moact* mutants was also reduced (see Figure S11D and S11F). These data suggested that the deletion of either *Mossadh* or *Moact* equally results in decreased activity of peroxidases and laccases in *M. oryzae*, similar to the *Moap1* mutants.

## Discussion

In this study, we have characterized *M. oryzae* MoAP1 as a homolog of fungal AP1 protein, such as *S. cerevisiae* Yap1 and *Schiz. pombe* Pap1. Similar to other members of the AP1 family [12], [29], MoAP1 contains a bZIP domain at the N-terminal, a nuclear localization motif, and c-CRD and/or n-CRD domains that are vital for cellular localization of Yap1 and resistance to oxidative damages [20]. In both filamentous and unicellular fungi, the nuclear localization of AP1 under the oxidative stress is a crucial step for the function of transcription regulation [27], and intramolecular disulfide bridge formation by two cysteine residues from the c-CRD and n-CRD regions is thought to be necessary for its nuclear translocation [41], [61]. It has also been established that the AP1 proteins are translocated in the nucleus in response to the oxidative stress [20], [62]. We have found that MoAP1 could partially complement the H_2_O_2_-sensitive phenotype of a yeast *yap1* mutant, *Moap1* deletion mutants were sensitive to H_2_O_2_, and MoAP1 localized in the nucleus when exposed to H_2_O_2_, all suggesting that MoAP1 is vital for resistance to the oxidative damage in *M. oryzae*.

To survive, fungi have evolved sophisticated mechanisms for adapting to stresses from intracellular or extracellular sources. During developmental processes, a fungus encounters various stresses, including the toxic by-products of its metabolism and the oxidative stress generated through aerobic respiration [63], [64]. It has also been documented that the cellular environment within host plants also serves as one of the major sources of stress to invading fungal pathogens [65], [66]. To evade the stress, fungi need specialized adaptation mechanisms. It is known that the AP1 transcription factors are one of the major regulators to activate genes in responding to the exogenous oxidative stress. In *Schiz. pombe*, two parallel H_2_O_2_-responsive pathways exist. The transcription factor Pap1, together with the MAP kinase Sty1 pathway, regulate gene expression in response to the oxidase stress [67]. In *S. cerevisiae* and *Candida albicans*, deletion of *YAP1* leads to reduced tolerance to the oxidative stress and/or attenuated virulence [21], [22], [62]. Studies of *U. maydis* also showed that Yap1 plays a critical role in the H_2_O_2_ detoxification system and in the infection of maize plants [15]. A similar role was also found for *A. alternata* AaAP1 [14]. In *C. heterostrophus* and *A. fumigatus*, the AP1 proteins ChAP1 and AfAP1 mediate H_2_O_2_ response but are not required for pathogenicity [14]. This indicates that the AP1 proteins are conserved regulators of the oxidative stress, but their roles in virulence are diverged. The *M. oryzae* AP1 protein appears to be close to *U. maydis* AP1 and AaAP1 in that it is required for both H_2_O_2_ resistance and pathogenicity. As a step to further advance our knowledge of AP1 proteins, we utilized the transcription analysis by the SAGE approach to identify genes involved in redox homeostasis whose expression is also down-regulated due to *Moap1* mutation. Based on our results, we hypothesize that MoAP1 is a key factor in regulating genes involved in the detoxification of H_2_O_2_, and that the severely restricted infectious hyphal growth of the *Moap1* mutants may be due to the reduced tolerance of the oxidative stress, which is responsible for the loss of pathogenicity.

ROS production serves many functions in eukaryotic cells, including those in cellular defense [13], [68], [69]. Its role in host resistance and pathogen invasion is highly dependent on the types of plant pathogen and host interaction [9], [70], [71], [72]. In plants, the generation of ROS is regarded as one of the first responses to fungal invasion [9]. For pathogens and entophytes, the production of ROS also has an important role and the disruption of intracellular ROS homeostasis can result in functional defects. In the endophytic fungus *Epichloë festucae*, ROS acts as a negative regulator to prohibit excessive fungal proliferation and, thus, allow the fungus to maintain a mutualistic relationship with its host plant [73]. Furthermore, the deletion of *NOXA* encoding NADPH oxidase or its regulatory subunit *RacA* leads to defects in ROS production, but the mutant remains highly pathogenic [73], [74]. However, the deletion of the *NOX*-like gene in the ergot fungus *Claviceps purpurea* yields mutants with reduced conidial formation and pathogenicity [75]. Moreover, the NADPH oxidases of both *M. oryzae* and the gray mold fungus *Botrytis cinerea* are key for the generation of intracellular ROS and their deletions make them lose pathogenicity on host plants [46], [76]. Conversely, deletion of the *AbTMPL* gene encoding a transmembrane protein in *A. brassicicola* led to hypersensitivity to the oxidative stress and excess oxidative bursts during conidiation and plant invasion, and ultimately impaired the virulence on green cabbage [77]. Thus, ROS generation seems to have different effects during fungi and plant interactions, and the regulation of ROS levels is essential for fungal development as well as virulence [46], [69], [72], [73], [76], [77], [78]. In this study, we highlighted the significance of intracellular ROS homeostasis in relation to fungal development. Because *MoAP1* was highly expressed in conidiation and the *Moap1* mutant exhibited abnormal conidiogenesis, excess ROS accumulation in conidia, and loss of pathogenicity, we concluded that MoAP1 is involved in important mechanisms for balancing ROS levels during conidiation, and the disruption of intracellular ROS homeostasis is responsible for the loss of virulence.

In *M. oryzae*, deletion of the catalase-encoding gene (*MoCATB*) caused reduced pigmentation, which is similar to the *Moap1* mutants because of its hyperoxidant state [49]. In this study, we also observed that the activity of the secreted laccase was reduced in *Moap1* mutants, and that the addition of copper sulfate stimulated the laccase activity and restored melanin biosynthesis to the *Moap1* mutants. This indicated that the decreased laccase activity and the excess ROS levels resulted in less pigmentation in the *Moap1* mutants.

In fungi, secreted peroxidases are regarded as an important component in helping pathogens to detoxify host-derived ROS during plant-microbe interactions [15], [16], [60]. Recently, it was shown that the decreased expression of peroxidase genes likely resulted in a reduced ability to scavenge host-derived ROS and, thus, an attenuation of virulence [15], [16], [60]. We detected a deficiency of secreted peroxidase activity in the *Moap1* mutant by comparing CR discoloration and assaying the peroxidase activity in culture filtrates. In the comparison of the SAGE data, we identified several extracellular peroxidase-encoding genes displaying significant reductions in transcription (see Table S1), which suggested that these may result in the decreased peroxidase activity in the *Moap1* mutants. Furthermore, the activity of the laccase, which is involved in pathogenicity of certain fungi [79], was severely reduced in the *Moap1* mutants. Moreover, we also identified that the laccase activity was similarly decreased in the *Mossadh* and *Moact* mutants. Such results prompted us to question whether the decreased laccase activity in the *Mossadh* and *Moact* mutants was associated with that displayed in the *Moap1* mutant. Expression of two laccase genes (*MGG_13464* and *MGG_11608*) was indeed down-regulated in the *Moap1* mutants (see Figure S8B). Based on these results, we postulated that the decreased expression of *MoSSADH* and *MoACT* is responsible for the reduced laccase activity in the *Moap1* mutants.

The identification of MoAP1-regulated genes by SAGE analysis revealed similar functional categories of genes that are down-regulated in the *Moap1* mutant. Among these genes were some well-studied ones directly involved in pathogenicity and novel ones such as *MGG_01230* and *MGG_15157*, which are likely to be involved in H_2_O_2_ tolerance. Disruption of either gene showed the complete loss of pathogenicity. The individual mutants also displayed similar morphogenesis defects, including less aerial hyphae but more compact mycelial growth, hypersensitivity to H_2_O_2_, decreased or loss of extracellular laccases and peroxidases activity, and reduced hyphal branching. These phenotypes are consistent with those observed in the *Moap1* mutants, indicating that the MoAP1 has a direct role in regulating genes such as *MGG_01230* and *MGG_15157* to control growth and morphologies, as well as tolerance to H_2_O_2_ and ultimately pathogenicity. Likewise, disruption of MoAP1 function may also affect other aspects such as protein translation and degradation, and secondary metabolism as evidenced by the SAGE data (see Table S1) that lead to the defect in pathogenicity.

Like most fungal pathogens, conidiogenesis and appressorium development are key steps in the colonization of host plants by *M. oryzae*. After attaching to the host surface, the conidia began to produce germ tubes and then developed a specialized infection structure, an appressorium with 8 MPa turgor pressure at the tip of the tube, to help the fungus penetrate host cell barriers [36]. The above processes are controlled by a precise developmental program in response to stimuli from the host and environment. In this program, conidiogenesis is a complex process that involves a cascade of morphological events. In *M. oryzae*, *MoAP1* disruption did not affect the developmental stages, such as hyphal growth, appressorium formation and penetration, but severely affected the ability to produce conidia and infectious hyphae growth. This observation was consistent with the results of qRT-PCR, which suggests that MoAP1 is a stage-specific regulator during conidiation and infection. In an effort to fully understand the developmental defects of the *Moap1* mutants, we screened the SAGE data and found that the zinc finger transcription factor MoCOS1 [54], a determinant of conidiophore formation, was also severely down-regulated in the *Moap1* mutants (see Figure S8A), indicating the possibility that it is responsible for decreased conidiation. A detailed analysis of the *MoAP1* downstream genes revealed that the mutation of either *Mossadh* or *Moact* gene caused complete loss of the ability to generate conidia. Taken together, these findings suggest that the transcriptional factor MoAP1 controls conidiation via a complex mechanism, involving the regulation of *MoCOS1*, *MoSSADH*, and *MoACT* expression. Certainly, we still could not rule out the possibility that some unknown gene(s) regulated by MoAP1 are also responsible for the conidiation in *M. oryzae*.

Because both the *Mossadh* and *Moact* mutants lost the ability to produce conidia, the pathogenicity assay was performed through inoculation of the hyphal tip plug that showed the complete loss of pathogenicity for the two mutants. It is known that appressorium-mediated penetration is required for full virulence of *M. oryzae* [80], [81], [82], [83], [84]. Recent studies of MoCos1 and other transcription factors revealed that the hyphae tips of the *MoCos1* mutant could develop appressorium-like structures on the host surface and cause infection [54], [58]. It was presumed that these transcription factors might play a role in an unknown mechanism in mycelia-mediated infection. To fully understand the loss of pathogenicity, we compared the formation of the appressorium at the hyphae tips between the wild-type strain and the two mutants, and found that all of them could form such appressorium-like structures. This finding is similar to the results of Kim et al. [58]. However, such hyphae-driven appressoria by the *Mossadh* and *Moact* deletion mutants were severely limited in their ability to penetrate into onion skin cells and rice leaf sheath cells and cause disease symptoms on leaves. This indicates that the hyphae-driven appressoria-mediated penetration may be functionally similar to penetration by normal appressoria. In the *Mossadh* and *Moact* mutants, the loss of pathogenicity might be due to the loss in the penetration ability.

The γ-aminobutyrate (GABA) shunt is a metabolic pathway that bypasses two successive steps of the tricarboxylic acid (TCA) cycle and is present in many organisms [85], [86], [87]. In plants, the activity of this pathway is predominantly associated with the response to biotic and abiotic stresses [88]. Previous reports have considered that a mutation of *AtSSADH* in *Arabidopsis thaliana* leads to growth abnormalities, hypersensitivity to the environmental stress, and ROS accumulation on the trichomes [87]. However, the cellular function of succinic semialdehyde dehydrogenase is still uncharacterized in fungal pathogens. In *M. oryzae*, our findings revealed that the *Mossadh* mutant displayed retarded mycelial growth, hypersensitivity to oxidative stress, and dramatically reduced aerial hyphae. Further, the mutant completely lost the ability to cause infections. We initially suspected that the lost of pathogenicity in the *Mossadh* mutant might be due to the inability to penetrate the host cell, but after injection of mycelial fragments in the rice leaf cell, the *Mossadh* mutant still could cause necrosis around the injection site. Together with the fact that stress-tolerance mechanisms of plant pathogens play an important role in virulence [14], [15], [16], [60] and the sensitivity of the *Mossadh* mutants to H_2_O_2_, we postulated that *M. oryzae* MoSsadh might be responsible for both the penetration and invasive growth *in planta*, and the defects in stress-tolerance may be a result of the restriction of invasive hyphae growth *in planta*, causing the complete loss of pathogenicity.

## Materials and Methods

### Fungal strains and growth conditions

*M. oryzae* strain Guy11 was used as wild type throughout this work. Both Guy11 and its derivative mutants were cultured on complete medium (CM) [89] for 3–15 days at 28°C to assess the growth and colony characteristics. Fungal mycelia were harvested from liquid CM and used for genomic DNA and RNA extractions. To observe the vegetative growth under the oxidative stress condition, H_2_O_2_ (Aldrich, 323381, 3 wt. %) was mixed in solid CM, and diameters of fungal colonies were measured after 3 to 5 days as indicated. For the activation of the laccase activity in the *Moap1* mutants, copper sulphate was amended in the CM medium for 1 mM at final concentration. Cell wall integrity assay was performed by growing strains in Congo Red (CR, Aldrich, 860956) amended CM medium (200 µg/ml) for 5 days. For conidia collection, strains were normally maintained on corn meal (RDC) medium [43] at 28°C for 10 days and then transferred to constant fluorescent light condition to promote conidiation for another 3–5 days. Conidia were obtained by rubbing mycelia with water followed by filtration through Miracloth (Calbiochem, San Diego, USA). For mycelial growth assay, strains were inoculated in the liquid complete medium for 48 hrs and then transferred to the Petri dish for photograph. To observe conidiophore development and conidiation, strains were inoculated on RDC for 5 days and mycelia were rubbed with a glass rod before transferring to the constant fluorescent light condition to promote conidiation for another 2 days. *S. cerevisiae* strains were grown in SD medium supplemented with appropriate amino acids and with glucose (3% (w/v)) or galactose (2% (w/v)). All growth assays were repeated for three times, with three replicates each time.

### Characterization of gene disruption mutants

Vegetative growth of *Moap1* mutants was measured on complete agar medium for 5 days, while the vegetative growth of *Mossadh* and *Moact* mutants were measured on the same medium for 4 days. All the experiments were performed with triple replicates in three independent experiments. The ability to produce conidia was measured by counting the numbers of conidia from 10-day old RDC plates as described previously [43]. Conidia were collected by rubbing the plate with 5 ml of sterilized distilled water. Conidia were counted using a hemacytometer under a microscope and conidial morphology was visualized under an Olympus inversion microscope at 40× magnification. Conidial germination and appressorium formation were measured on a hydrophobic coverslip. Conidial suspensions of 30 µl (1×10^5^ spores/ml) were dropped onto a coverslip and placed in a moistened box at 28°C. After 8 hrs of incubation, the percentage of conidia germinating and germinated conidia-forming appressoria was determined by microscopic examination of at least 100 conidia. This test was done at least three times, each with three replications.

### Yeast *yap1* mutant complementation

*S. cerevisiae* BY4741ΔYML007w (*yap1*) and the strain from which it was derived, BY4741 (MATa *his3Δ1 leu2Δ0 met2Δ0 ura3Δ0*) were obtained from Euroscarf. The full-length of *M. oryzae Moap1* cDNA (1.7 kb) was amplified using primers pairs FL2700(F)/FL2701(R). The PCR products, digested with *EcoRI* and *SphI*, were cloned into pYES2 (Invitrogen) and transformed into BY4741ΔYML007w. Colonies were selected on SD medium lacking uracil, and the wild type yeast strains BY4741 as well as the *yap1* deletion mutant BY4741ΔYML007w transformed with empty pYES2 vector were used as a control. Transformed yeast cells were grown on SD medium without uracil containing either glucose 3% (w/v) or 2% galactose respectively. Five-microliter drops from serial dilutions from cultures with an OD600 of 0.5 were spotted on plates with and without 0.3 mM H_2_O_2_ and grown for 3 days at 30°C.

### Targeted gene disruption and complementation of *MoAP1* and MoAP1 target genes

For constructing the *Moap1* gene replacement construct, a 1.0-kb upstream flanking sequence fragment and 0.9-kb downstream flanking sequence was amplified from *M. oryzae* genomic DNA by PCR using primer pairs FL1992(F)/FL1993(R) and FL1994(F)/FL1995(R), respectively (Table S2). The two flanking sequences were joined together by overlap PCR with primer pair FL1992(F) and FL1995(R), and the amplified ∼2 kb fragments were purified and cloned into a pMD19-T vector (Takara Co, China) to generate plasmid pMDT–*Moap1*. The Hygromycin resistance gene cassette [90] was prepared by PCR amplification with primer pair FL1111(F)/FL1112(R) (Table S2) and inserted in the plasmid pMDT–*Moap1* at the *PmeI* enzyme site to generate the final disruption construct pMD–*Moap1-HPH*. The 3.4-kb fragment was amplified with FL1992(F) and FL1995(R) primers and transformed into Guy11 protoplasts. The protoplast-mediated transformation of *M. oryzae* Guy11 was carried out as described [88]. Transformants were selected on solid CM agar medium supplemented with 300 µg/ml hygromycin B. To identify the gene-deleted mutants, Hygromycin B resistant transformants were screened using primers FL2382(F)/FL2383(R) (Table S2). The mutants were further verified by Southern hybridization. To generate the complementation of the *Moap1* mutant, a 4.4 kb DNA fragment including the putative promoter and the coding sequence was amplified and inserted into the plasmid pCB1532, according to Zhang *et al* [88]. Disruption of *MoAP1* target genes was performed similarly with primer pairs listed in the Table S2.

### Nucleic acid manipulation and Southern blotting

DNA extraction was performed as described by Talbot and associates [88], while gel electrophoresis, restriction enzyme digestion, ligation, and Southern blot hybridization were performed using standard procedures [91]. DNA hybridization probes were random primer labeled with digoxigenin-11-dUTP using DIG-High prime according to the manufacturer's instructions for digoxigenin high-prime DNA labeling and the detection starter kit (Roche Applied Science, Penzberg, Germany). Total RNA was isolated from frozen fungal mycelia using the RNA extraction kit (Macherey-Nagel, Bethlehem, PA, USA) following the manufacturer's instructions. To measure the relative abundance of gene transcripts, RNAs were extracted from mycelia grown in CM liquid medium for 2 days at 28°C in a 150-rpm orbital shaker. To measure the relative abundance of *MoAP1* transcripts during diverse fungal developmental stages, the total RNA samples were extracted from mycelia grow in CM liquid medium, conidia and plants inoculated with the conidia of Guy11 (1×10^8^ spores ml^−1^) for 8, 24, 48 and 72 hrs, respectively, by the method described above. The primer sets used to detect transcripts of *MoAP1* and its related genes from *M. oryzae* are listed in Table S2.

### Quantitative RT–PCR, RT–PCR, and gene expression analysis

For RT-PCR and quantitative real time RT-PCR (qRT-PCR), 5 µg of total RNA were reverse transcribed into first-strand cDNA using the oligo(dT) primer and M-MLV Reverse Transcriptase (Invitrogen). Confirmation of deletions and reintroduction of *MoAP1*, *MoSSADH* and *MoACT* genes were made with primer pairs FL2382(F)/FL2383(R), FL6745(F)/FL6746(R), and FL6749 (F)/FL6750(R) (Table S2). 32 cycles of RT-PCR were run on a Bio-Rad PTC0200 Peltier Thermal Cycler. The stable expression actin gene (*MGG_03982.5*) amplified by primer pairs FL474(F)/FL475(R) (Table S2) was used as internal control.

qRT-PCR reactions were performed following previously established procedures [16]. To compare the relative abundance of target gene transcripts, the average threshold cycle (Ct) was normalized to that of actin gene for each of the treated samples as 2^−ΔCt^, where -ΔCt  =  (C_t, target gene_-C_t, actin gene_). Fold changes during fungal development and infectious growth in liquid CM were calculated as 2^−ΔCt^, where -ΔCt  = (C_t, target gene_- C_t, actin gene_) _test condition_-(C_t, WT_- C_t, actin gene_) CM [16]. qRT-PCR was performed with three independent pools of tissues in three sets of experimental replicates and primers pairs used in this section were listed in Table S2.

### Plant infection assays

Onion epidermis penetration assays were performed using the method as previously described [43]. Plant infection assays were performed on four-week old susceptible rice seedlings (*O. sativa*) CO-39 or seven-day old barley seedlings (Four arris) by spraying 4 ml of the conidial suspensions with a sprayer. Inoculated plants were placed in a moist chamber at 28°C for first 24 hrs in darkness, and then transferred back to another moist chamber with a photoperiod of 12 hrs under fluorescent lights. The disease severity was assessed at 5 or 7 days after inoculation. Approximately six centimeter long diseased rice blades were photographed to evaluate the virulence of the mutants. For determining the pathogenicity of the mutants without conidia, mycelia tip plugs of the wild type strain Guy11, *Mossadh* and *Moact* mutants were inoculated on the healthy or wounded rice leaves or barley leaves for 5 or 7 days and kept in the same condition as described above. For the infiltration infection assay, 0.1 gram mycelia of the tested strains was broken into pieces using a glass rod and 50 µl of each suspension were injected into the leaves of 4-week-old rice plants and cultured for 5 or 7 days under the condition as described above. These experiments were all replicated three times.

### ROS and superoxide detection

Intracellular ROS levels of *M. oryzae* were monitored during the infection related structure formation using the oxidant-sensitive probe dihydrohodamine-123 (Molecular Probes, Carlsbad, CA) and nitroblue tetrazolium (NBT) as previously described [16]. For dihydrorhodamine-123 staining, drops of conidial suspension (30 µl) were placed on the coverslips and cultured for up to 24 hrs. At each interval, the water surrounding the conidia was removed carefully and replaced with final concentration of 50 µM dihydrorhodamine-123 (Merck, Whitehouse Station, NJ) at 28°C for 2 hrs, then rinsed twice with phosphate-buffered saline and viewed under a fluorescence microscope (Olympus IX71) equipped with a digital camera by short exposure to UV light. NBT staining was performed as described [92]. Superoxide production during conidia germination and infection related structure formation was viewed by microscopy.

### Measurement of the extracellular enzyme activities

Laccase activity on solid medium was measured as described [92] with little modification. A 5×5 mm hyphal tip plug was inoculated on CM medium supplemented with 0.2 mM 2, 2′-azino-di-3-ethylbenzathiazoline- 6-sulfonate (ABTS, Sigma) for 3 days. The assay for the activation of the laccase activity was performed by addition of 1 mM copper sulphate to the CM medium containing 0.2 mM ABTS and cultured under 28°C for 4 days. For detection of peroxidase secretion, a 5×5 mm hyphal tip plug was placed on CM medium containing 200 µg/ml Congo Red for 5 days. The measurement of peroxidase and laccase activities in culture filtrates was performed as described [88].

### CFW and DAPI staining

Calcofluor staining using Fluorescent Brightener 28 (10 µg/ml, Sigma-Aldrich) for the microscopy of mycelial branches was performed as described [93]. Both the mutants and the wild type were inoculated on the coverslips that contain a thin layer of agar medium and cultured for 48 hrs. Mycelial tip plugs were removed and stained with 10 µg/ml CFW for 10 min in darkness, rinsed twice with PBS and viewed under a fluorescence microscope (Olympus IX71). For the localization of MoAP1, conidia of Guy11 transformed with plasmid pCB1532::*TrpC*::*Moap1*::*eGFP* was treated with 2 mM H_2_O_2_, stained with DAPI (50 µg/ml, Sigma) for 10 min, and visualized under a microscope (Olympus IX71). GFP fluorescence was detected using a 450 to 490-nm excitation filter and a 520-nm barrier filter and the DAPI fluorescence was detected under UV light using a 360 to 400 nm excitation filter.

### Bioinformatics analysis

The full sequence of *MoAP1* was downloaded from the *M. oryzae* database (www.broadinstitute.org/annotation/genome/magnaporthe_grisea). Yap1 homology sequences from different organisms were obtained from GenBank (www.ncbi.nlm.nih.gov/BLAST) using the BLAST algorithm [94]. Sequence alignments were performed using the Clustal_W program [95] and the phylogenetic tree was viewed using Mega3.0Beta program [96]. Orthologs were identified between *M. oryzae* predicted proteins and proteins in the GO database [97] via searching reciprocal best hits with the following cut-offs; e-value, 1.0e-3, and identity, 20%. Results from local alignment using BLAST and prediction of signal peptides from SignalP 3.0 software [98] and a manual literature review were used to make final assignments to GO functional categories. Primers used in this study were designed by using Primer3 Input (version 0.4.0) and commercially synthesized (Invitrogen Co., Shanghai, China). To predict AP1 binding sites, yeast AP1 binding motif sequences (MTTACGTAAK, TTAGTMAGC and TTASTMA) [99], [100], [101] were used to search in the 1000 bp- upstream sequences set of the up- and down-regulated genes from SAGE, and no more than one mismatch was allowed.

## Supporting Information

Figure S1 Comparison of AP1 protein conserved domains and a dendrogram of fungal AP1 proteins. (A) Comparison of the conserved bZIP domains of fungal AP1 proteins arranged by Clustal W program. Identical amino acids residues are shaded. (B) Alignment of the conserved c-CRD domain of fungal AP1 proteins. Asterisks indicate the conserved cysteine residues. (C) A dendrogram of fungal AP1 proteins. The phylogenetic tree was created with Mega3.0 beta by the established parameter in the program. GenBank accession numbers are as follows: *M. oryzae* MoAP1 (EDK00544), *F. oxysporum* FoAP1 (XP_388976), *C. heterostrophus* ChAP1 (AAS64313), *P. tritici-repentis* PtAP1 (XP_001931984), *A. oryzae* AorAP1 (BAE92562), *S. sclerotiorum* SSAP1 (EDN93694), *B. fuckeliana* BC1G (EDN20443), *G. zeae* GzAP1 (XP_388976), *N. crassa* NCAP1 (CAB91681), *K. lactis* KLULA (AAC39320), *S. pombe* SpPAP1 (CAB66170), *S. cerevisiae* ScYAP1 (CAA41536), *A. alternata* AaAP1(ACM50933), *U. maydis* YAP1 (XP_758338) and *C. albicans* CAP1P (EAK94712).
(1.75 MB TIF)

Figure S2 MoAP1 complements the H_2_ O_2_ sensitivity of a *S. cerevisiae* Δ *yap1* mutant. The growth of *S. cerevisiae* BY4741+pYES2, BY4741DYML007w+pYES2, BY4741DYML007w+pYES2::*Moap1* was tested on SD plates with glucose (top left panel), galactose (top right panel), SD plates with glucose supplemented with 0.3 mM H_2_O_2_ (bottom left panel), and with glucose supplemented with 0.3 mM H_2_O_2_ (bottom right panel).
(1.36 MB TIF)

Figure S3 The *MoAP1* phase specific expression, targeted gene replacement and complementation. (A) The phase specific expression of *MoAP1*. The expression of *MoAP1* was measured by quantitative real-time RT-PCR with cDNA from samplings for infectious growth, vegetative growth, and conidia. The relative abundance of *Moap1* transcripts during infectious growth (from ungerminated conidia to in planta fungal cells 72 hpi) was normalized by comparing with vegetative growth in liquid CM (Relative transcript level  =  1). Each sample was harvested from 10 plants and three independent experiments, each with three replicates, were performed. Significant differences are presented in the figure (P < 0.01), and the error bar represents the standard deviation. (B) *MoAP1* targeted gene replacement. A 1.89-kb fragment of the *Moap1* coding region was replaced with a 1.4-kb fragment containing the hygromycin B resistance cassette to create the *Moap1* mutant. The DNA fragment at the inner space of *MoAP1* deletion region was used as the probe to validate the *Moap1* deletion transformants by PCR amplification and Southern hybridization analysis (scale bar  =  1 kb). (C) Genomic PCR was used to validate the deletion of *Moap1* gene and reintroduction of *Moap1* coding region to complement the mutant strain. (D) Semiquantitative RT-PCR was carried out to confirm the deletion and reintroduction of *MoAP1* gene. Complete inactivation of *Moap1* transcription in the deletion mutants was verified by reverse transcription (RT)-PCR using cDNA of the wild type strain, the *Moap1* mutants, and the complemented strain. (E and F) Southern hybridization analysis was used to validate the deletion of the *MoAP1* gene and the addition of a single copy integration of the *HPH* gene. The arrowhead in E (left) showed a single band hybridized by the *HPH* gene probe in the mutant. No band was present in the wild type strain.
(1.24 MB TIF)

Figure S4 Hyphal branching reduction in the *Moap1*, *Mossadh*, and *Moact* mutants. (A) Branching patterns of mycelia on agar media containing coverslips 48 hrs after incubation. Frequent branching occurs at the mycelia of wild type while no or a few hyphal branches were observed in the *Moap1* mutants. Calcofluor white staining is used to indicate the position of the mycelia. Bar  =  50 μm. (B) Branching patterns of mycelia on agar media containing coverslips 48 hours after incubation. Frequent branching occurs at the mycelia of wild type while no or a few hyphal branches were observed in the *Mossadh* mutants. Calcofluor white staining indicates the position of the mycelia. Bar  =  50 μm. (C) Branching patterns of mycelia on agar media containing coverslips 48 hrs after incubation. Frequent branching occurs at the mycelia of wild type while no or a few hyphal branches were observed in the *Moact* mutants. Calcofluor white staining is used as the indicator for the position of the mycelia. Bar  =  50 μm.
(2.19 MB TIF)

Figure S5 Subcellular localization of MoAP1 in the presence of H_2_O_2_. For green fluorescence observation, both conidia of wild type strain Guy11 (WT) and WT transformed with pCB1531::*TrpC*::*Moap1*::*eGFP* were treated with DAPI and then observed under an Olympus microscope with a specific filter set as described in the materials and methods. For subcellular localization, conidia of WT transformed with pCB1531::*TrpC*::*Moap1*::*eGFP* was treated with or without 2 mM H_2_O_2_ and then observed as described above.
(0.70 MB TIF)

Figure S6 Pathogenicity test of *Moap1*, *Mossadh* and *Moact* mutant strains on the barley leaves. (A) Pathogenicity test of *Moap1* mutant on barley leaves. 4 ml conidial suspension (1×10^5^ conidia/ml) of each strain was sprayed on seven-day-old barley seedlings (*Four arris*) and cultured as described in Figure 5A and the results were observed at 7 dpi. The barley leaves spraying of gelatin was used as negative control. (B and C). Pathogenicity test of *Moap1*, *Mossadh* and *Moact* mutant on barley leaves. Mycelia blocks of the wild type strain Guy11, *Moap1*, *Mossadh*, *Moact*, and the complemented strains were inoculated on seven-day-old barley leaves and then cultured as described in Materials and Methods. The barley leaves with the CM agar plugs on was used as negative control.
(3.33 MB TIF)

Figure S7 Laccase activity is restored by addition of copper sulphate. The assay for the activation of the laccase activity in the mutants was performed by addition of 1 mM copper sulphate to CM media that contains 0.2 mM laccase substrate ABTS and cultured under 28°C for 4 days to observe the phenotype. The photo on top showed *Moap1* mutants inoculated on CM media containing 0.2 mM ABTS, and the photo at middle showed *Moap1* mutants inoculated on CM media amending 1 mM copper sulphate, while the picture at bottom indicated *Moap1* mutants inoculated on CM media supplemented with both 1 mM copper sulphate and 0.2 mM ABTS.
(1.11 MB TIF)

Figure S8 Expression profiles of *MoCOS1* and putative laccase-encoding genes in *Moap1*, *Mossadh*, and *Moact* mutants. (A) The transcript levels of MoCOS1-encoding genes in both the *Moap1* mutant and the wild type strain were indicated from three independent experiments. Error bars represent the standard deviations. (B) The transcript levels of the two putative laccase-encoding genes in the *Moap1*, *Mossadh*, *Moact*, and Guy11 were indicated from three independent experiments. Error bars represent the standard deviations.
(0.37 MB TIF)

Figure S9 Pathogenicity test of *Mossadh* and *Moact* mutants on wounded rice plants. (A) Pathogenicity test of the mutant strain by injection of hyphal fragments. The hyphal fragments of the strains tested were treated as described in Materials and Methods and the results were scored at 7 dpi. (B) Pathogenicity test of *Mossadh* and *Moact* mutants. The mycelia blocks of the strains were inoculated on the wounded rice leaves as described above and then cultured under moist condition at 28°C for 7 days.
(1.56 MB TIF)

Figure S10 Mycelia growth of *Mossadh* and *Moact* mutant strains on two synthetic medium. (A and B) Phenotypes of Guy11, *Mossadh*, *Moact*, and complemented strains. Strains were inoculated on CM medium and cultured as described in the Materials and Methods. (C and D) Phenotypes of Guy11, *Mossadh*, *Moact* mutants, and the complemented strain on RDC media. The strains were cultured under darkness for 7 days at 28°C. (E) Statistical analysis of mycelia growth rate of Guy11, *Mossadh*, and the complemented strains on both CM and RDC agar media. Three independent experiments were performed and similar results were obtained. Error bars represent the standard deviations and asterisks represent significant differences in Guy11, *Mossadh* mutants and the complemented strain (p < 0.01). (F) Statistical analysis of mycelia growth rate of Guy11, *Moact* mutants and the complemented strains on both CM and RDC agar media. Error bars represent the standard deviation, and asterisks represent significant differences in Guy11, *Moact* mutants and the complemented strain (p < 0.01).
(3.30 MB TIF)

Figure S11 Compromised extracellular laccase and peroxidase activity displayed by *Mossadh* and *Moact* mutants. (A) Strains of Guy11, *Mossadh*, *Moact*, and the complemented strains were inoculated on CM agar medium containing 200 μg/ml Congo Red. The discoloration of Congo Red was observed after inoculation for 5 days. (B) The laccase activities of Guy11, *Mossadh*, *Moact*, and the complemented strains were monitored in complete media supplemented with 0.2 mM ABTS. The oxidized dark purple staining around the colony was observed after 3 days of incubation. (C and D) Strains of Guy11, *Mossadh*, and complemented strain were inoculated in CM liquid medium and peroxidase (C) and laccase activities (D) were measured in culture filtrates by ABTS oxidization test with or without H_2_O_2_. Error bars represent the standard deviations and asterisks indicated significant differences. (E and F) Guy11, *Moact* mutant, and the complemented strain were inoculated in CM liquid medium and peroxidase (E) and laccase (F) activities were measured as described above. The differences among Guy11, *Moact* mutants, and the complemented strain were statistically significant (p < 0.01).
(4.18 MB TIF)

Figure S12 Pathogenicity test of MoAP1 target gene disruption mutants. (A) Pathogenicity test of gene deletion mutants on the rice cultivar CO-39. The SAGE down-regulated gene deletion mutants were inoculated by spraying conidia suspensions on the four-week old rice cultivar CO-39 for 7 days and then photographed. (B) Pathogenicity test of *Mossadh* and *Moact* mutants on the rice cultivar CO-39 at 7 dpi with mycelial plugs.
(3.10 MB TIF)

Table S1 Categorization of MoAP1 regulated genes with known function.
(2.18 MB DOC)

Table S2 Primer pairs used in this paper.
(0.14 MB DOC)
