# Correction: The bZIP Transcription Factor MoAP1 Mediates the Oxidative Stress Response and is Critical for Pathogenicity of the Rice Blast Fungus *Magnaporthe oryzae*

**DOI:** 10.1371/journal.ppat.1008196

**Published:** 2019-11-20

**Authors:** Min Guo, Yue Chen, Yan Du, Yanhan Dong, Wang Guo, Su Zhai, Haifeng Zhang, Suomeng Dong, Zhengguang Zhang, Yuanchao Wang, Ping Wang, Xiaobo Zheng

The authors would like to correct images in S12 Fig. In S12A and S12B Fig, duplicate images were mistakenly illustrated during the preparation of figures for publication. The authors have now repeated the experiments and provided the new images. The authors confirm that these changes do not alter any findings.

## Supporting information

S12 Fig Pathogenicity test of the down-regulated gene deletion mutants. (A) Pathogenicity test of gene deletion mutants on the rice cultivar CO-39. The SAGE down-regulated gene deletion mutants were inoculated by spraying conidia suspensions on the four-week old rice cultivar CO-39 for 7 days and then photographed. (B) Pathogenicity test of *Mossadh* and *Moact* mutants on the rice cultivar CO-39 at 7 dpi with mycelial plugs.
(TIF)
