# Supplementary figures and images for: The bZIP Transcription Factor MoAP1 Mediates the Oxidative Stress Response and Is Critical for Pathogenicity of the Rice Blast Fungus Magnaporthe oryzae
Source: PLoS Pathog. 2011 Feb 24;7(2):e1001302. doi: 10.1371/journal.ppat.1001302 (PMC3044703; doi:10.1371/journal.ppat.1001302)

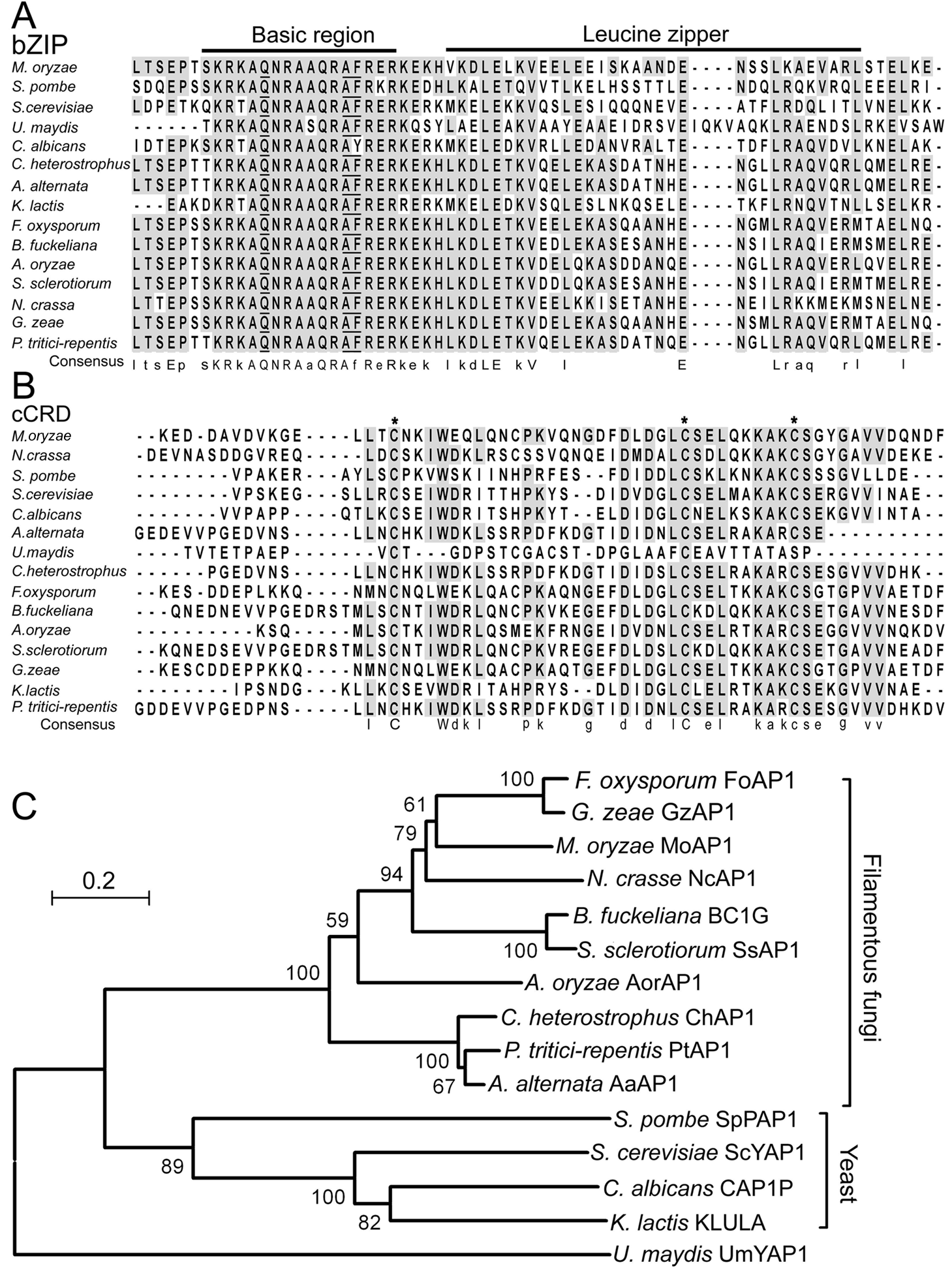

Supplement: Figure S1 — Comparison of AP1 protein conserved domains and a dendrogram of fungal AP1 proteins. (A) Comparison of the conserved bZIP domains of fungal AP1 proteins arranged by Clustal W program. Identical amino acids residues are shaded. (B) Alignment of the conserved c-CRD domain of fungal AP1 proteins. Asterisks indicate the conserved cysteine residues. (C) A dendrogram of fungal AP1 proteins. The phylogenetic tree was created with Mega3.0 beta by the established parameter in the program. GenBank accession numbers are as follows: M. oryzae MoAP1 (EDK00544), F. oxysporum FoAP1 (XP_388976), C. heterostrophus ChAP1 (AAS64313), P. tritici-repentis PtAP1 (XP_001931984), A. oryzae AorAP1 (BAE92562), S. sclerotiorum SSAP1 (EDN93694), B. fuckeliana BC1G (EDN20443), G. zeae GzAP1 (XP_388976), N. crassa NCAP1 (CAB91681), K. lactis KLULA (AAC39320), S. pombe SpPAP1 (CAB66170), S. cerevisiae ScYAP1 (CAA41536), A. alternata AaAP1(ACM50933), U. maydis YAP1 (XP_758338) and C. albicans CAP1P (EAK94712). (1.75 MB TIF) [file ppat.1001302.s001.tif]

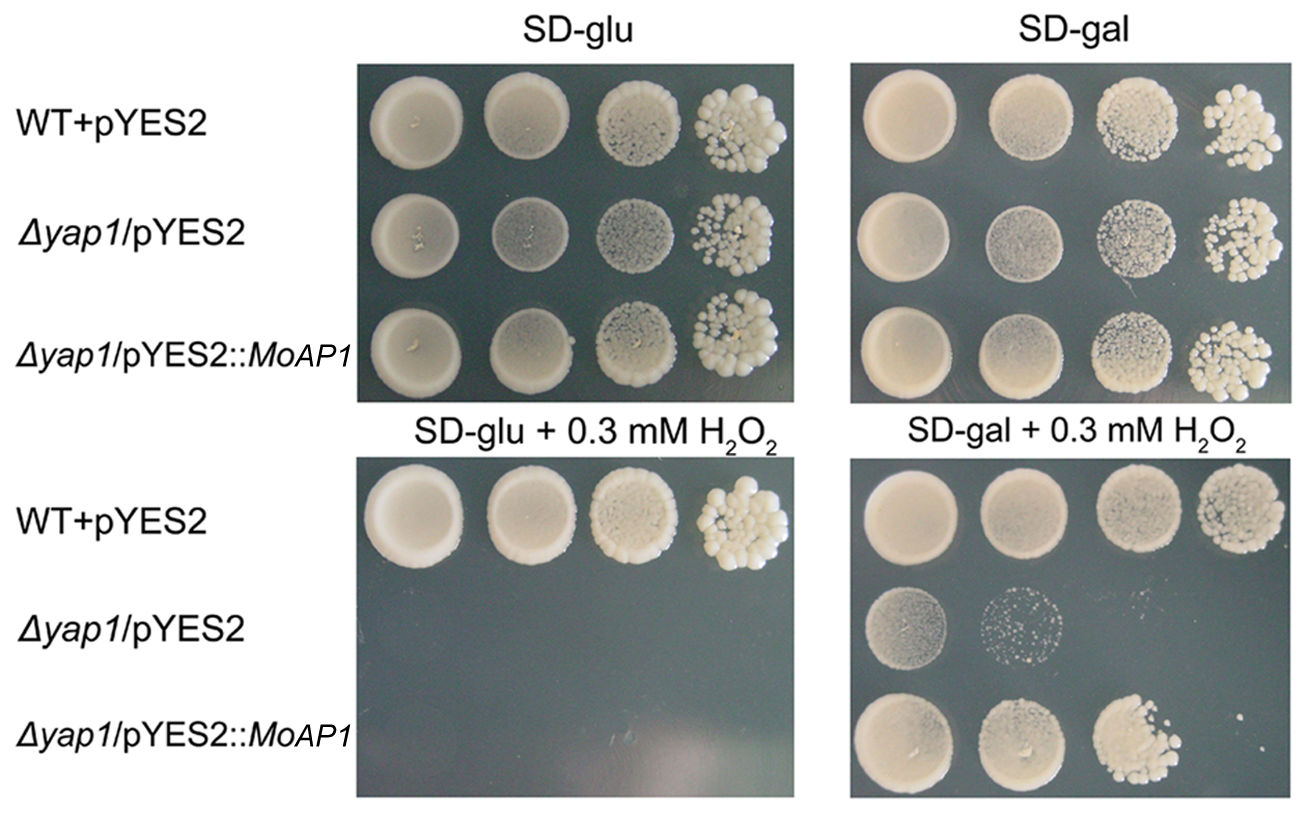

Supplement: Figure S2 — MoAP1 complements the H2 O2 sensitivity of a S. cerevisiae Δ yap1 mutant. The growth of S. cerevisiae BY4741+pYES2, BY4741DYML007w+pYES2, BY4741DYML007w+pYES2::Moap1 was tested on SD plates with glucose (top left panel), galactose (top right panel), SD plates with glucose supplemented with 0.3 mM H2O2 (bottom left panel), and with glucose supplemented with 0.3 mM H2O2 (bottom right panel). (1.36 MB TIF) [file ppat.1001302.s002.tif]

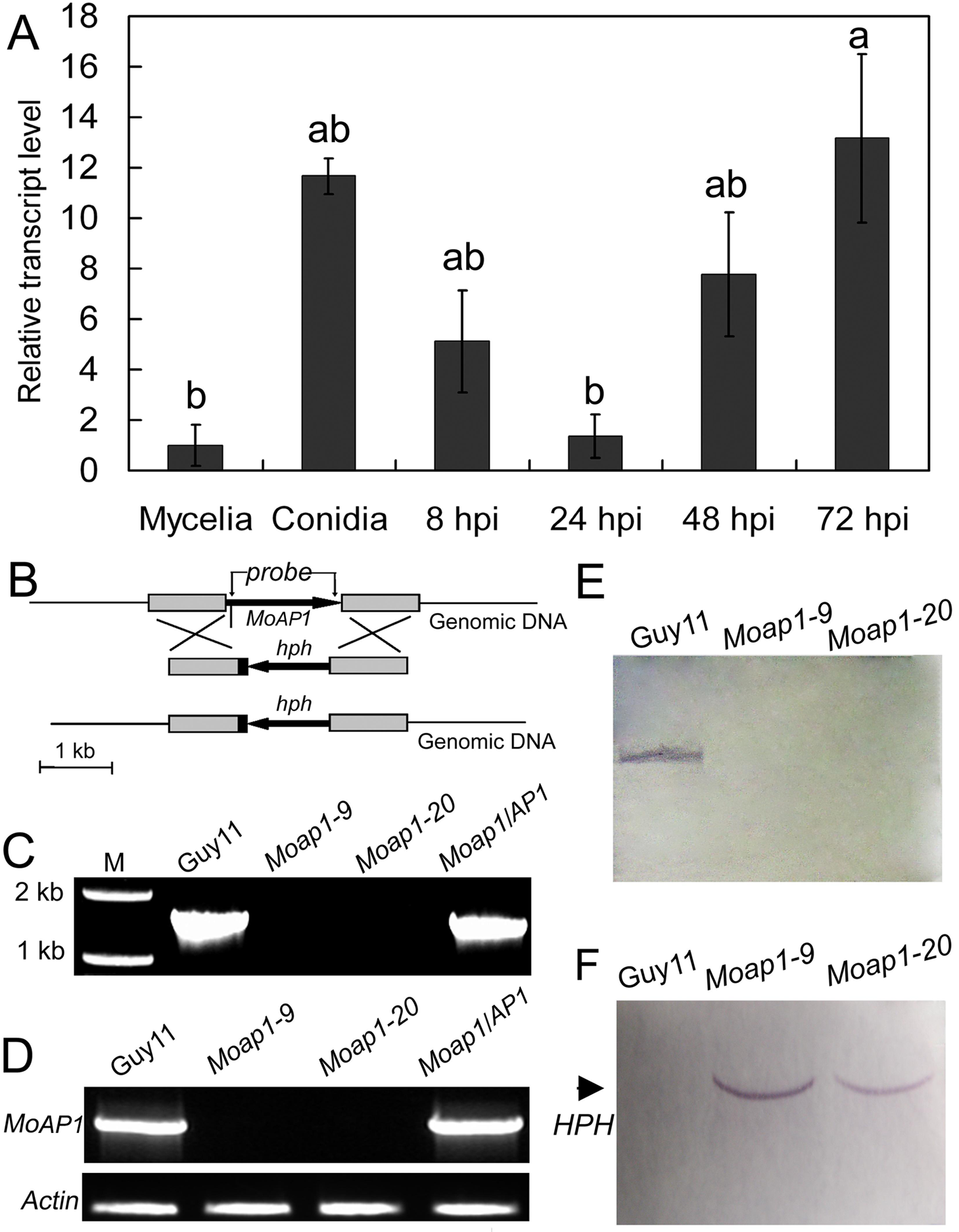

Supplement: Figure S3 — The MoAP1 phase specific expression, targeted gene replacement and complementation. (A) The phase specific expression of MoAP1. The expression of MoAP1 was measured by quantitative real-time RT-PCR with cDNA from samplings for infectious growth, vegetative growth, and conidia. The relative abundance of Moap1 transcripts during infectious growth (from ungerminated conidia to in planta fungal cells 72 hpi) was normalized by comparing with vegetative growth in liquid CM (Relative transcript level = 1). Each sample was harvested from 10 plants and three independent experiments, each with three replicates, were performed. Significant differences are presented in the figure (P < 0.01), and the error bar represents the standard deviation. (B) MoAP1 targeted gene replacement. A 1.89-kb fragment of the Moap1 coding region was replaced with a 1.4-kb fragment containing the hygromycin B resistance cassette to create the Moap1 mutant. The DNA fragment at the inner space of MoAP1 deletion region was used as the probe to validate the Moap1 deletion transformants by PCR amplification and Southern hybridization analysis (scale bar = 1 kb). (C) Genomic PCR was used to validate the deletion of Moap1 gene and reintroduction of Moap1 coding region to complement the mutant strain. (D) Semiquantitative RT-PCR was carried out to confirm the deletion and reintroduction of MoAP1 gene. Complete inactivation of Moap1 transcription in the deletion mutants was verified by reverse transcription (RT)-PCR using cDNA of the wild type strain, the Moap1 mutants, and the complemented strain. (E and F) Southern hybridization analysis was used to validate the deletion of the MoAP1 gene and the addition of a single copy integration of the HPH gene. The arrowhead in E (left) showed a single band hybridized by the HPH gene probe in the mutant. No band was present in the wild type strain. (1.24 MB TIF) [file ppat.1001302.s003.tif]

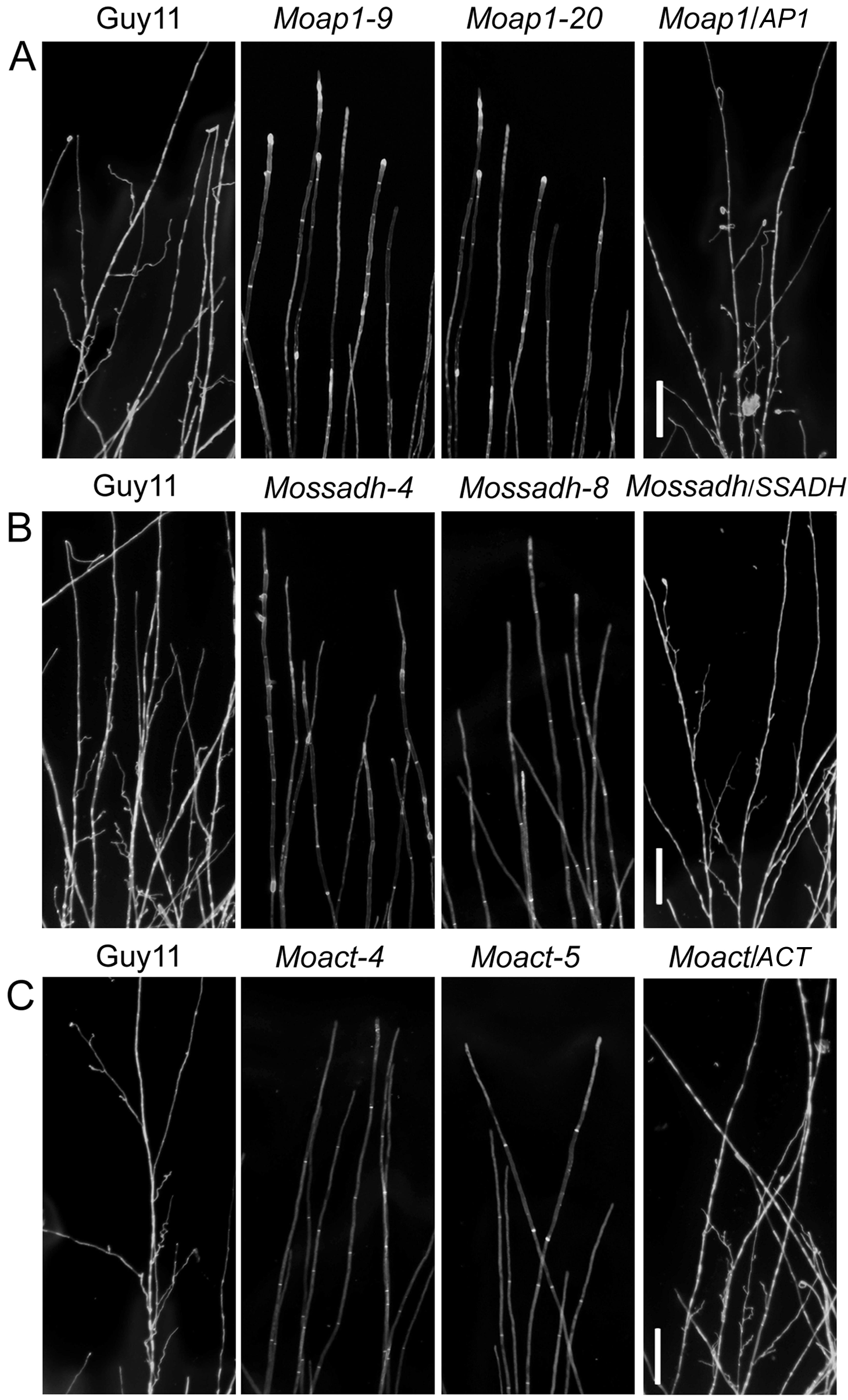

Supplement: Figure S4 — Hyphal branching reduction in the Moap1, Mossadh, and Moact mutants. (A) Branching patterns of mycelia on agar media containing coverslips 48 hrs after incubation. Frequent branching occurs at the mycelia of wild type while no or a few hyphal branches were observed in the Moap1 mutants. Calcofluor white staining is used to indicate the position of the mycelia. Bar = 50 μm. (B) Branching patterns of mycelia on agar media containing coverslips 48 hours after incubation. Frequent branching occurs at the mycelia of wild type while no or a few hyphal branches were observed in the Mossadh mutants. Calcofluor white staining indicates the position of the mycelia. Bar = 50 μm. (C) Branching patterns of mycelia on agar media containing coverslips 48 hrs after incubation. Frequent branching occurs at the mycelia of wild type while no or a few hyphal branches were observed in the Moact mutants. Calcofluor white staining is used as the indicator for the position of the mycelia. Bar = 50 μm. (2.19 MB TIF) [file ppat.1001302.s004.tif]

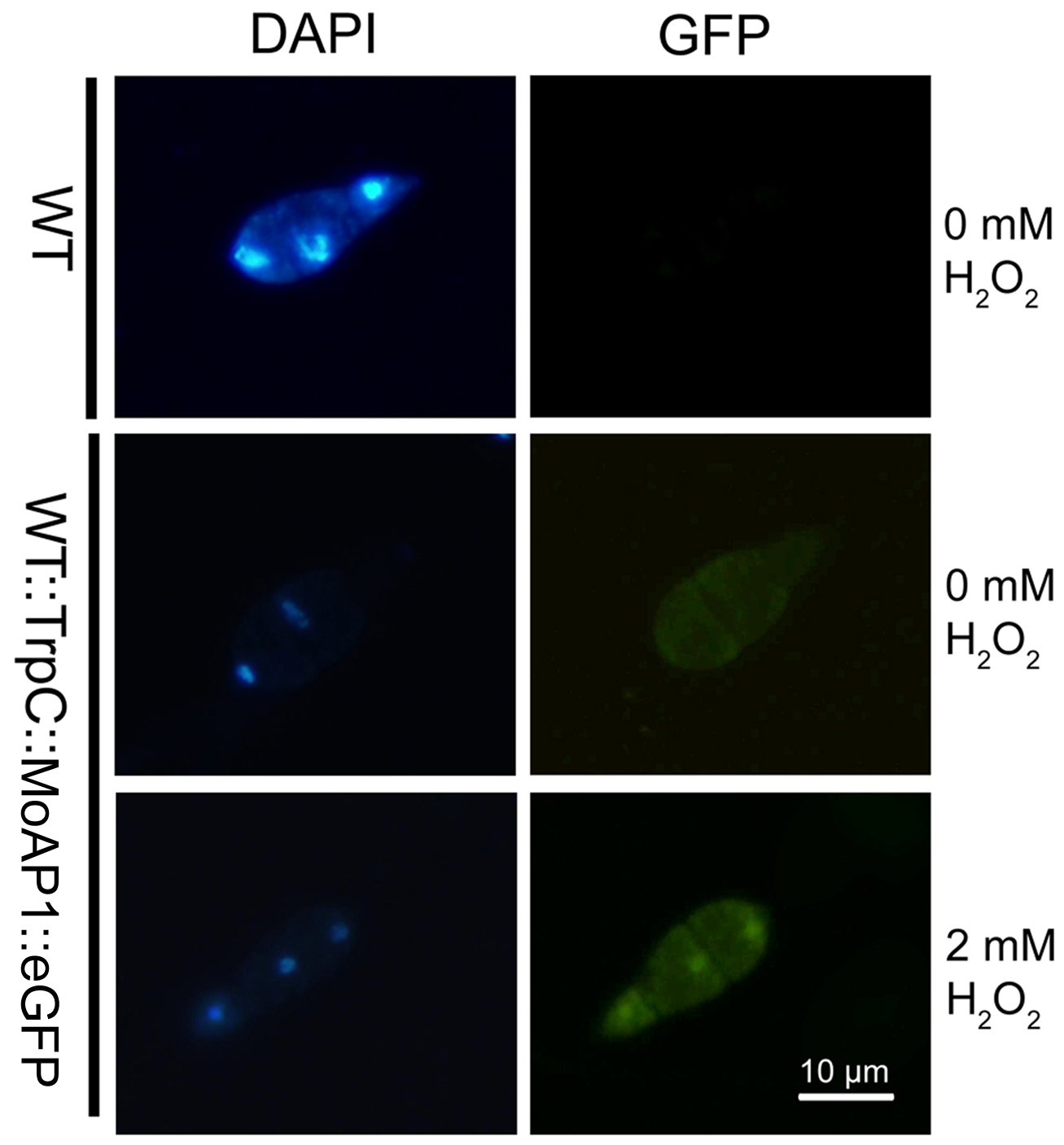

Supplement: Figure S5 — Subcellular localization of MoAP1 in the presence of H2O2. For green fluorescence observation, both conidia of wild type strain Guy11 (WT) and WT transformed with pCB1531::TrpC::Moap1::eGFP were treated with DAPI and then observed under an Olympus microscope with a specific filter set as described in the materials and methods. For subcellular localization, conidia of WT transformed with pCB1531::TrpC::Moap1::eGFP was treated with or without 2 mM H2O2 and then observed as described above. (0.70 MB TIF) [file ppat.1001302.s005.tif]

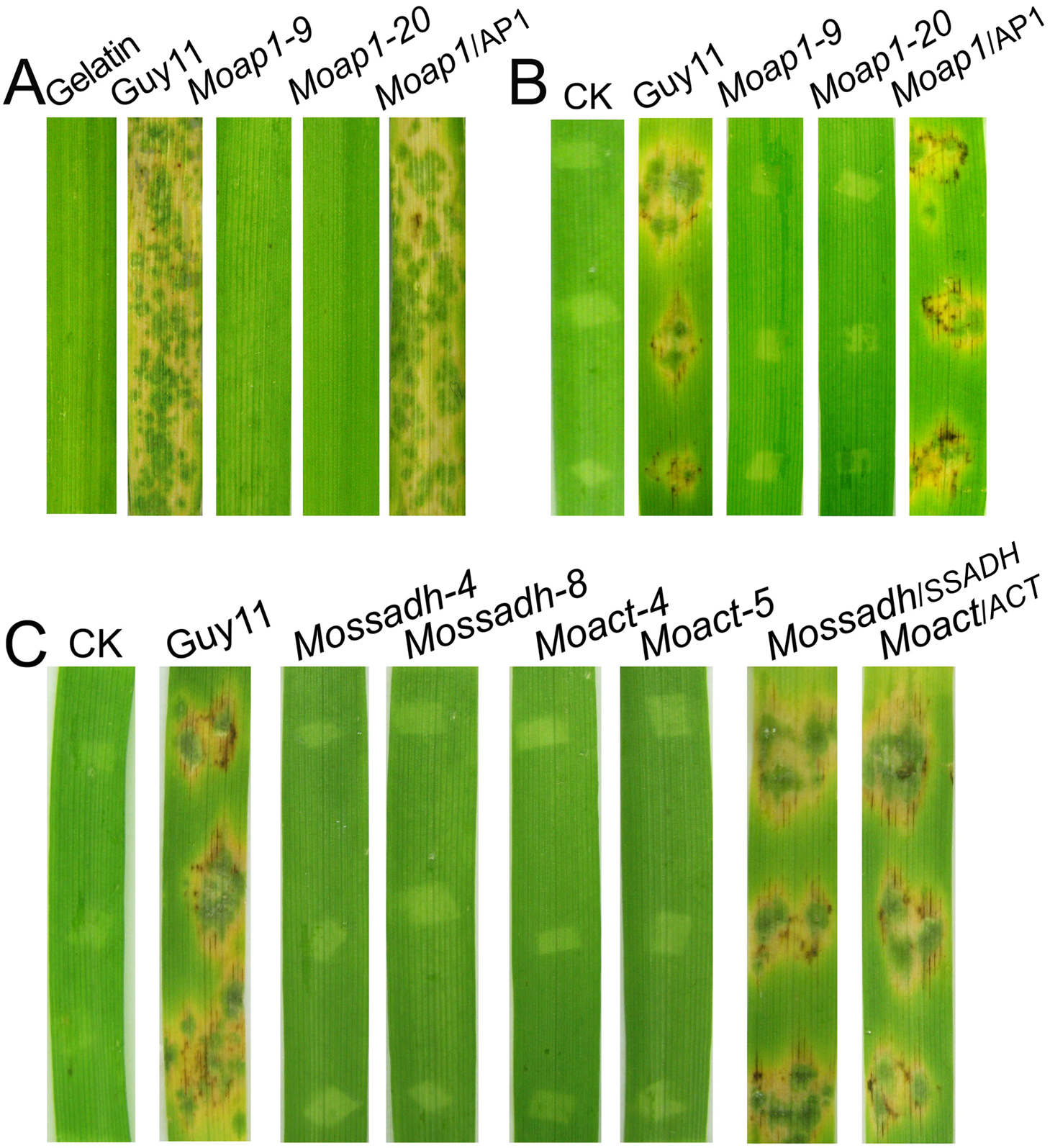

Supplement: Figure S6 — Pathogenicity test of Moap1, Mossadh and Moact mutant strains on the barley leaves. (A) Pathogenicity test of Moap1 mutant on barley leaves. 4 ml conidial suspension (1×10^5^ conidia/ml) of each strain was sprayed on seven-day-old barley seedlings (Four arris) and cultured as described in Figure 5A and the results were observed at 7 dpi. The barley leaves spraying of gelatin was used as negative control. (B and C). Pathogenicity test of Moap1, Mossadh and Moact mutant on barley leaves. Mycelia blocks of the wild type strain Guy11, Moap1, Mossadh, Moact, and the complemented strains were inoculated on seven-day-old barley leaves and then cultured as described in Materials and Methods. The barley leaves with the CM agar plugs on was used as negative control. (3.33 MB TIF) [file ppat.1001302.s006.tif]

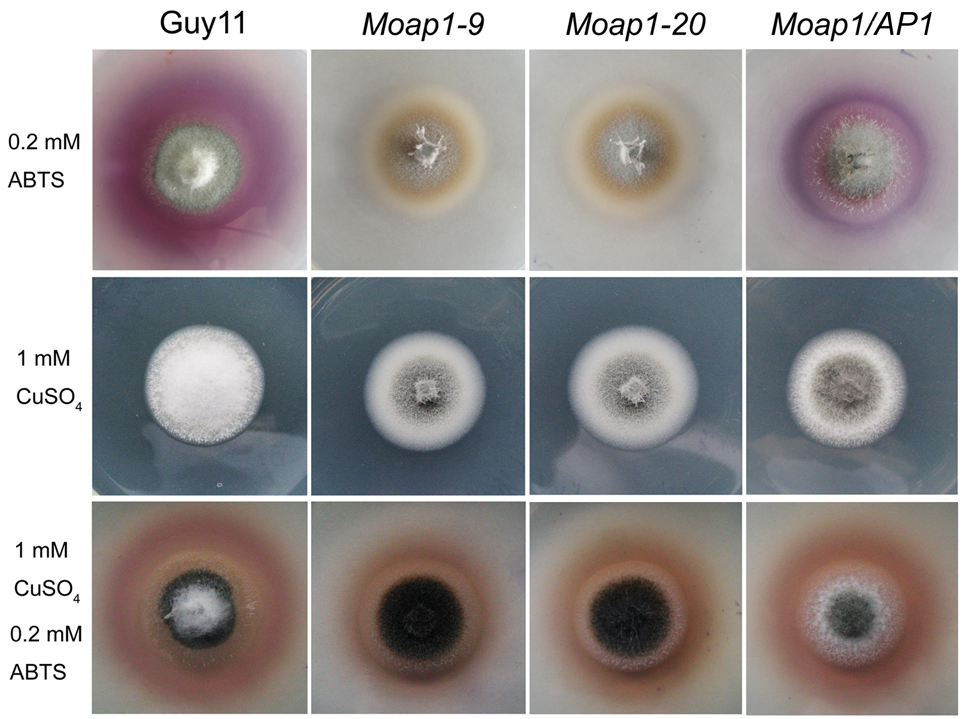

Supplement: Figure S7 — Laccase activity is restored by addition of copper sulphate. The assay for the activation of the laccase activity in the mutants was performed by addition of 1 mM copper sulphate to CM media that contains 0.2 mM laccase substrate ABTS and cultured under 28°C for 4 days to observe the phenotype. The photo on top showed Moap1 mutants inoculated on CM media containing 0.2 mM ABTS, and the photo at middle showed Moap1 mutants inoculated on CM media amending 1 mM copper sulphate, while the picture at bottom indicated Moap1 mutants inoculated on CM media supplemented with both 1 mM copper sulphate and 0.2 mM ABTS. (1.11 MB TIF) [file ppat.1001302.s007.tif]

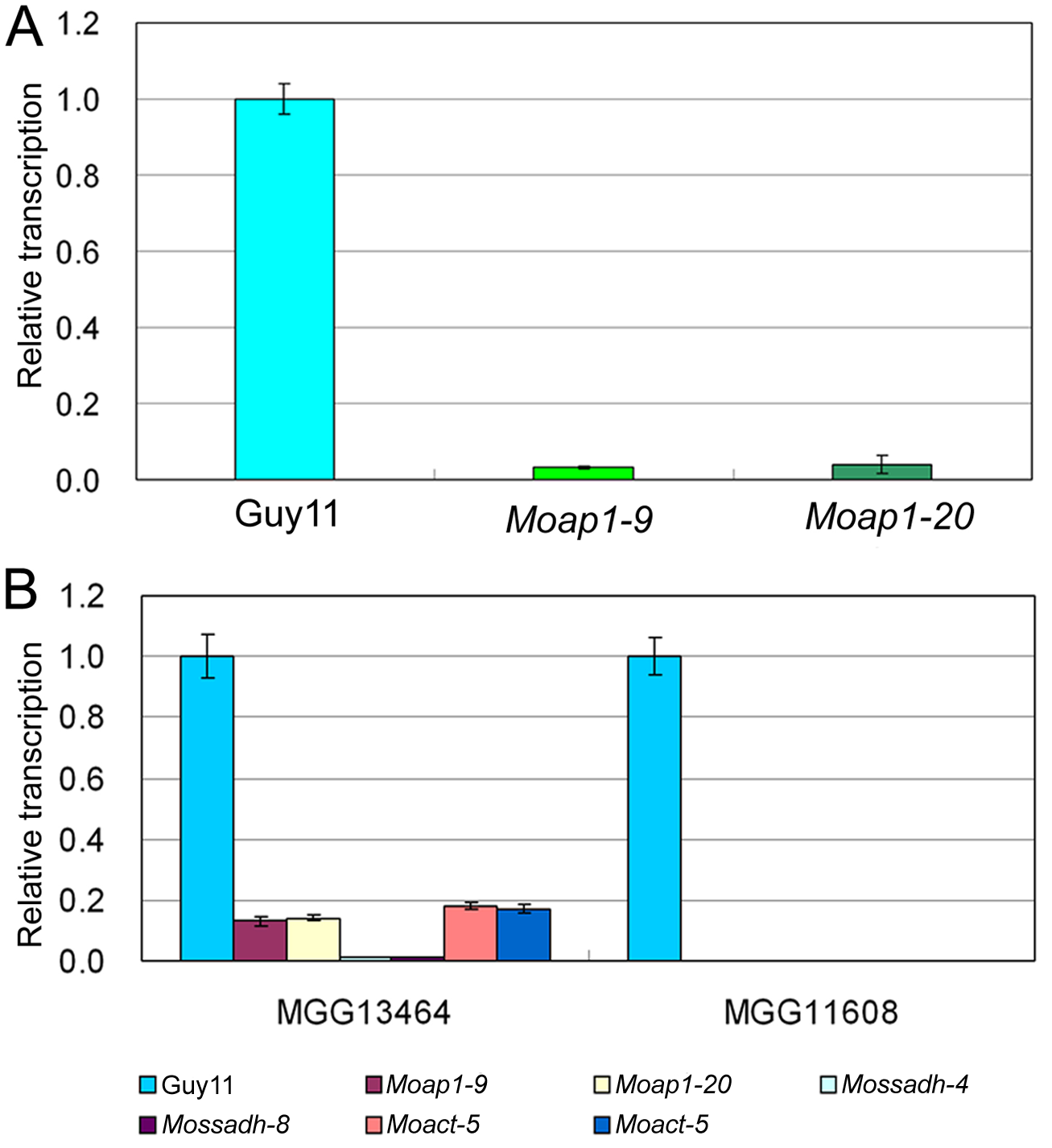

Supplement: Figure S8 — Expression profiles of MoCOS1 and putative laccase-encoding genes in Moap1, Mossadh, and Moact mutants. (A) The transcript levels of MoCOS1-encoding genes in both the Moap1 mutant and the wild type strain were indicated from three independent experiments. Error bars represent the standard deviations. (B) The transcript levels of the two putative laccase-encoding genes in the Moap1, Mossadh, Moact, and Guy11 were indicated from three independent experiments. Error bars represent the standard deviations. (0.37 MB TIF) [file ppat.1001302.s008.tif]

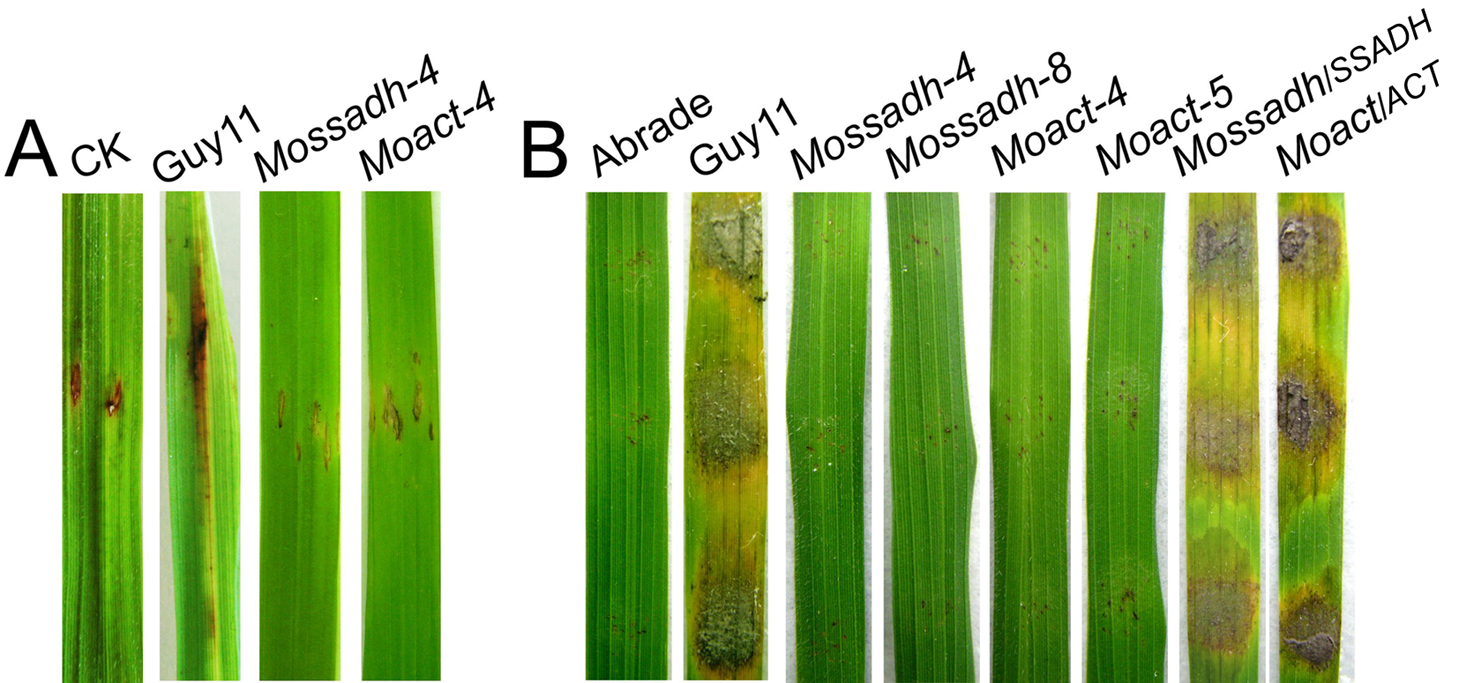

Supplement: Figure S9 — Pathogenicity test of Mossadh and Moact mutants on wounded rice plants. (A) Pathogenicity test of the mutant strain by injection of hyphal fragments. The hyphal fragments of the strains tested were treated as described in Materials and Methods and the results were scored at 7 dpi. (B) Pathogenicity test of Mossadh and Moact mutants. The mycelia blocks of the strains were inoculated on the wounded rice leaves as described above and then cultured under moist condition at 28°C for 7 days. (1.56 MB TIF) [file ppat.1001302.s009.tif]

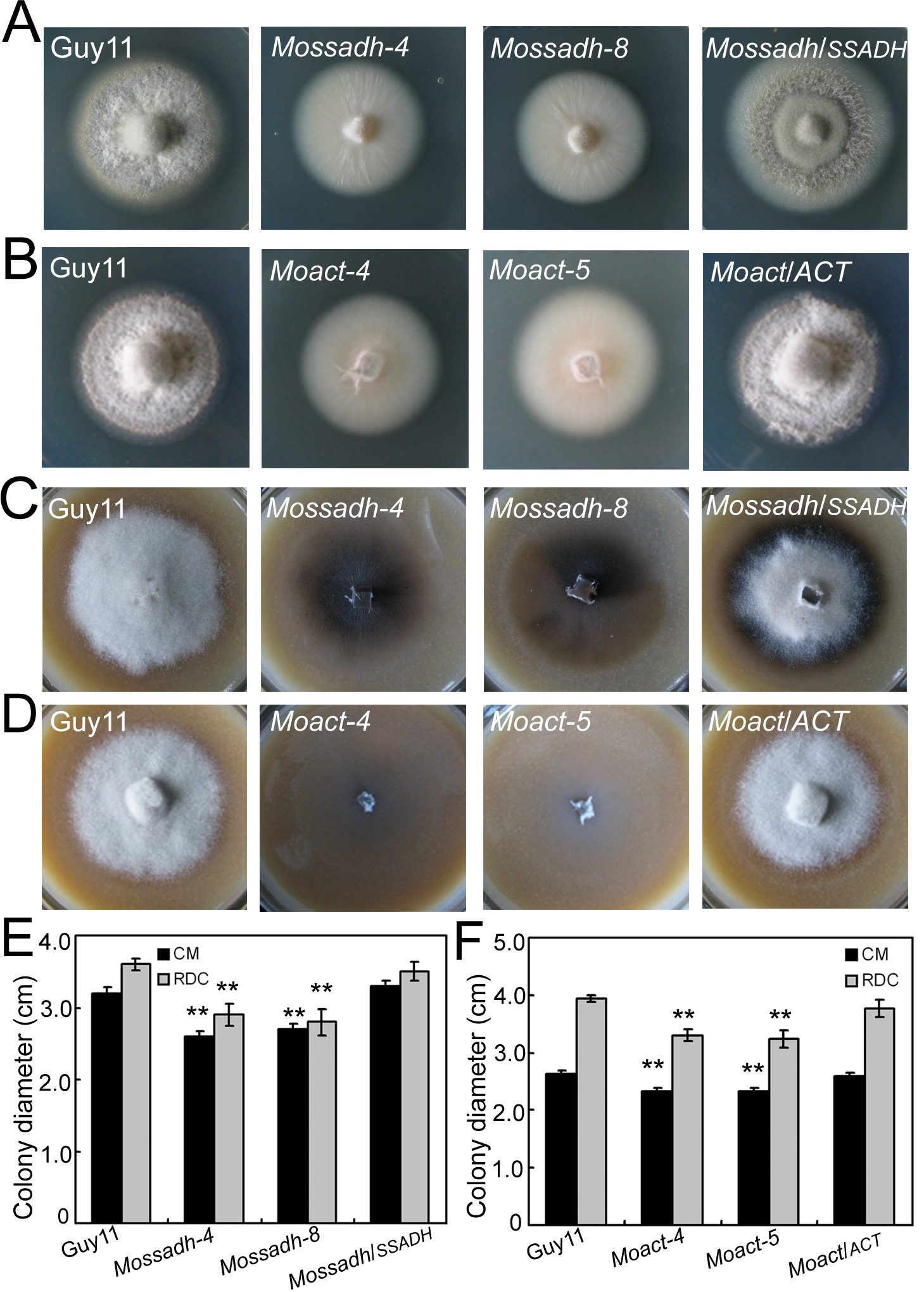

Supplement: Figure S10 — Mycelia growth of Mossadh and Moact mutant strains on two synthetic medium. (A and B) Phenotypes of Guy11, Mossadh, Moact, and complemented strains. Strains were inoculated on CM medium and cultured as described in the Materials and Methods. (C and D) Phenotypes of Guy11, Mossadh, Moact mutants, and the complemented strain on RDC media. The strains were cultured under darkness for 7 days at 28°C. (E) Statistical analysis of mycelia growth rate of Guy11, Mossadh, and the complemented strains on both CM and RDC agar media. Three independent experiments were performed and similar results were obtained. Error bars represent the standard deviations and asterisks represent significant differences in Guy11, Mossadh mutants and the complemented strain (p < 0.01). (F) Statistical analysis of mycelia growth rate of Guy11, Moact mutants and the complemented strains on both CM and RDC agar media. Error bars represent the standard deviation, and asterisks represent significant differences in Guy11, Moact mutants and the complemented strain (p < 0.01). (3.30 MB TIF) [file ppat.1001302.s010.tif]

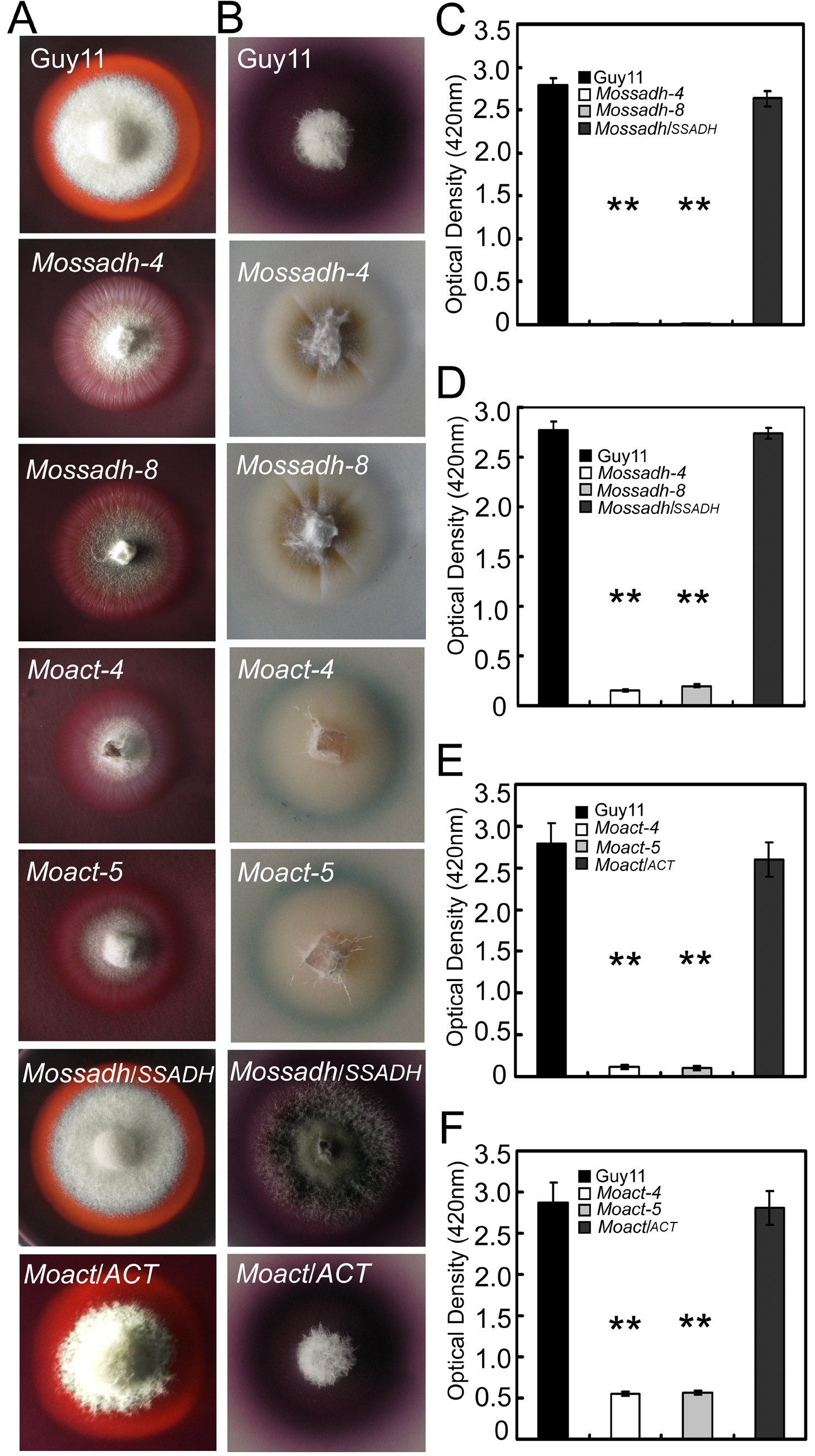

Supplement: Figure S11 — Compromised extracellular laccase and peroxidase activity displayed by Mossadh and Moact mutants. (A) Strains of Guy11, Mossadh, Moact, and the complemented strains were inoculated on CM agar medium containing 200 μg/ml Congo Red. The discoloration of Congo Red was observed after inoculation for 5 days. (B) The laccase activities of Guy11, Mossadh, Moact, and the complemented strains were monitored in complete media supplemented with 0.2 mM ABTS. The oxidized dark purple staining around the colony was observed after 3 days of incubation. (C and D) Strains of Guy11, Mossadh, and complemented strain were inoculated in CM liquid medium and peroxidase (C) and laccase activities (D) were measured in culture filtrates by ABTS oxidization test with or without H2O2. Error bars represent the standard deviations and asterisks indicated significant differences. (E and F) Guy11, Moact mutant, and the complemented strain were inoculated in CM liquid medium and peroxidase (E) and laccase (F) activities were measured as described above. The differences among Guy11, Moact mutants, and the complemented strain were statistically significant (p < 0.01). (4.18 MB TIF) [file ppat.1001302.s011.tif]

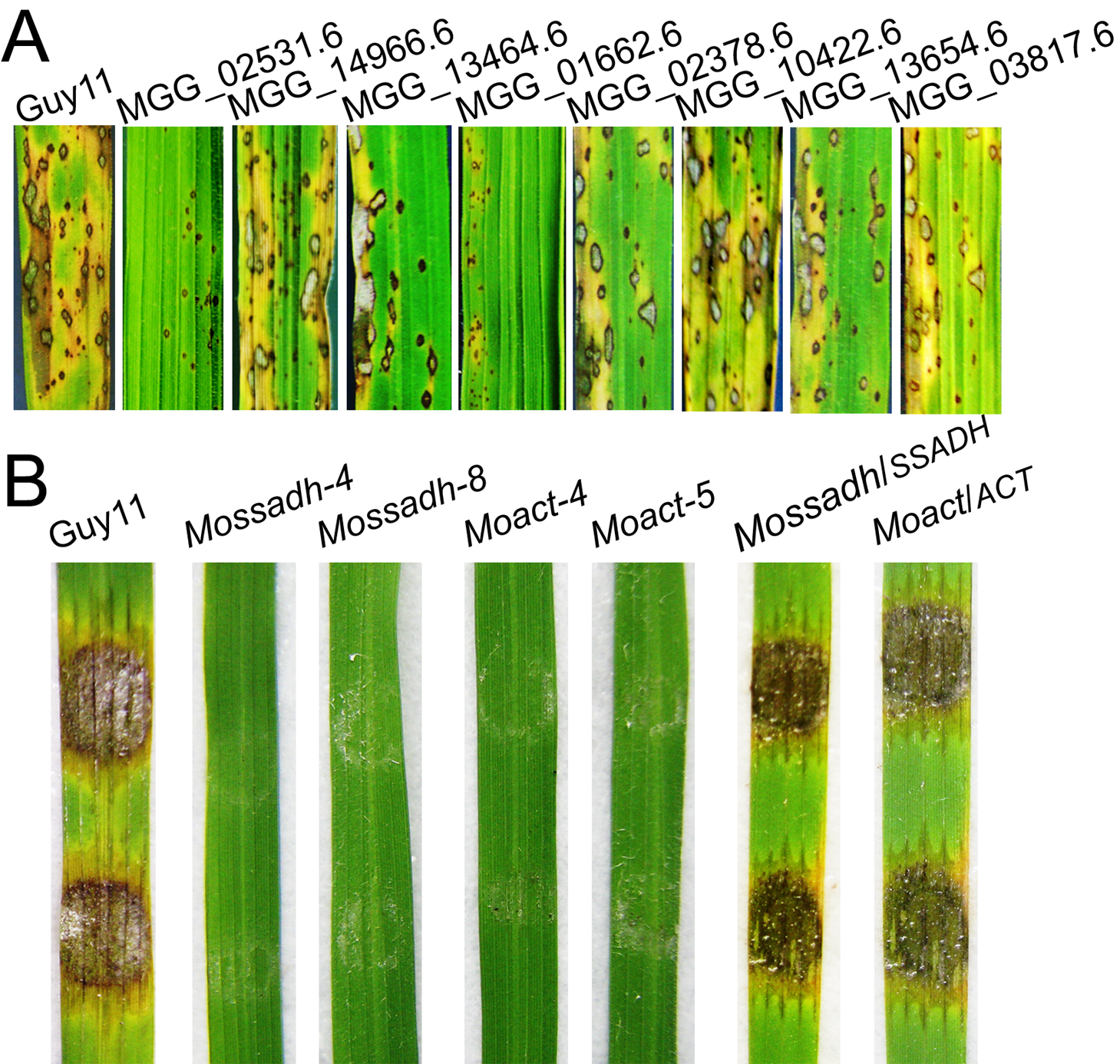

Supplement: Figure S12 — Pathogenicity test of MoAP1 target gene disruption mutants. (A) Pathogenicity test of gene deletion mutants on the rice cultivar CO-39. The SAGE down-regulated gene deletion mutants were inoculated by spraying conidia suspensions on the four-week old rice cultivar CO-39 for 7 days and then photographed. (B) Pathogenicity test of Mossadh and Moact mutants on the rice cultivar CO-39 at 7 dpi with mycelial plugs. (3.10 MB TIF) [file ppat.1001302.s012.tif]
