# Supplementary material for: The bZIP Transcription Factor MoAP1 Mediates the Oxidative Stress Response and Is Critical for Pathogenicity of the Rice Blast Fungus Magnaporthe oryzae
Source: PLoS Pathog. 2011 Feb 24;7(2):e1001302. doi: 10.1371/journal.ppat.1001302 (PMC3044703; doi:10.1371/journal.ppat.1001302)
Supplement: Table S1 — Categorization of MoAP1 regulated genes with known function. (2.18 MB DOC) [file ppat.1001302.s013.doc]

**Table S1. Categorization of MoAP1 regulated genes with known function**

| Category | Gene I D ^a^ | Exp.^b^ | SignalP ^c^ | | | Blast hit | NCBI ID | E-value^d^ |
| --- | --- | --- | --- | --- | --- | --- | --- | --- |
| Secondary metalbolism ( GO:0019748) | | | | | | | | |
| 2.59 | MGG_04911* | DR | N | | cytochrome P450 78A3 [Pyrenophora tritici-repentis Pt-1C-BFP]. | | XP_001936907 | 2e-72 |
| 2.32 | MGG_06585 | DR | N | | reductase [Cercospora nicotianae]. | | ABK64183 | 8e-59 |
| Signal transduction (GO:0007165) | | | | | | | | |
| 1.96 | MGG_02450 | DR | N | | Pc21g02000 [Penicillium chrysogenum Wisconsin 54-1255]. | | XP_001404457 | 6e-83 |
| 1.58 | MGG_00261 | DR | N | | F-box and WD40 domain protein, putative [Neosartorya fischeri NRRL 181]. | | XP_001263278 | 3e-127 |
| 2.32 | MGG_01818 | UR | N | | MAGA [Magnaporthe grisea]. | | AAB65425 | 0.0 |
| 2.17 | MGG_03700* | UR | N | | Ras GTPase activating protein, putative [Talaromyces stipitatus ATCC 10500]. | | EED20452 | 0.0 |
| 2.17 | MGG_12709* | UR | N | | extragenic suppressor of kinetochore protein 1 [Pyrenophora tritici-repentis Pt-1C-BFP]. | | XP_001931757 | 0.0 |
| 2.00 | MGG_12886* | UR | N | | calmodulin, putative [Penicillium marneffei ATCC 18224]. | | XP_002149912 | 2e-56 |
| 2.00 | MGG_03241* | UR | N | | arrestin domain protein [Aspergillus clavatus NRRL 1]. | | XP_001268158 | 2e-168 |
| 1.80 | MGG_03232 | UR | N | | cell morphogenesis protein Sog2, putative [Neosartorya fischeri NRRL 181]. | | XP_001267300 | 1e-141 |
| 1.58 | MGG_01150 | UR | N | | C2 and Extensin domain protein [Aspergillus flavus NRRL3357]. | | EED52565 | 4e-67 |
| 1.58 | MGG_15156* | UR | N | | TOR kinase [Gibberella fujikuroi]. | | CAJ44735 | 0.0 |
| 1.58 | MGG_11425* | UR | N | | neurofibromatosis type 1 [Takifugu rubripes]. | | AAD15839 | 0.0 |
| 1.58 | MGG_09961* | UR | N | | WD domain containing protein [Pyrenophora tritici-repentis Pt-1C-BFP]. | | XP_001937903 | 1e-72 |
| 1.58 | MGG_01813 | UR | N | | mediator of replication checkpoint 1 (PMID 14585996) [Schizosaccharomyces pombe]. | | NP_594486 | 1e-13 |
| 1.58 | MGG_06439* | UR | N | | SH3 domain protein, putative [Talaromyces stipitatus ATCC 10500]. | | EED14881 | 6e-98 |
| Amino acid Metabolism (GO:0006520) | | | | | | | | |
| 4.67 | MGG_08074* | DR | N | | NADP-specific glutamate dehydrogenase [Neurospora crassa OR74A]. | | XP_961561 | 0.0 |
| 4.45 | MGG_02378* | DR | N | | glutamate decarboxylase [Chaetomium globosum CBS 148.51]. | | XP_001228383 | 0.0 |
| 2 | MGG_05223 | DR | N | | beta isopropylmalate dehydrogenase. | | 2004294A | 2e-169 |
| 1.96 | MGG_04244* | DR | N | | proline oxidase, putative [Talaromyces stipitatus ATCC 10500]. | | EED16574 | 3e-106 |
| 1.85 | MGG_14171* | DR | N | | L-serine dehydratase [Pyrenophora tritici-repentis Pt-1C-BFP]. | | XP_001939098 | 1e-96 |
| 1.58 | MGG_05827* | DR | N | | glutamyl-tRNA(Gln) amidotransferase subunit A [Pyrenophora tritici-repentis Pt-1C-BFP]. | | XP_001930848 | 2e-168 |
| 1.58 | MGG_11389* | DR | N | | l-allo-threonine aldolase [Aspergillus clavatus NRRL 1]. | | XP_001275648 | 4e-123 |
| 1.58 | MGG_03962* | DR | Y | | tyrosinase [Aspergillus fumigatus Af293]. | | XP_74842 | 2e-36 |
| 1.58 | MGG_00149* | DR | N | | serine/threonine-protein phosphatase PP-Z [Neurospora crassa OR74A]. | | XP_965036 | 0.0 |
| 2.32 | MGG_02205* | UR | N | | alcohol dehydrogenase, putative [Aspergillus flavus NRRL3357]. | | XP_365923 | 7e-79 |
| 2.32 | MGG_13783* | UR | N | | aspartate--tRNA ligase [Cryptococcus neoformans var. neoformans JEC21]. | | XP_571738 | 0.0 |
| 2.32 | MGG_07781* | UR | N | | quinate 5-dehydrogenase [Neurospora crassa]. | | CAA32751 | 2e-109 |
| 2.00 | MGG_00912 | UR | N | | protein-tyrosine phosphatase 2 [Penicillium marneffei ATCC 18224]. | | XP_002143996 | 6e-31 |
| 2.00 | MGG_07545* | UR | N | | CTP synthase [Neurospora crassa OR74A]. | | XP_957732 | 0.0 |
| 2.00 | MGG_05519* | UR | N | | quinone oxidoreductase, putative [Talaromyces stipitatus ATCC10500]. | | EED12502 | 2e-85 |
| 1.58 | MGG_01182* | UR | N | | glycerate dehydrogenase [Pyrenophora tritici-repentis Pt-1C-BFP]. | | XP_001937212 | 1e-90 |
| 1.58 | MGG_07528 | UR | N | | imidazoleglycerol-phosphate dehydratase [Neurospora crassa OR74A]. | | XP_961386 | 2e-98 |
| 1.58 | MGG_09299 | UR | N | | peroxisome biosynthesis protein (PAS1/Peroxin-1), putative [Penicillium marneffei ATCC 18224]. | | XP_002146350 | 0.0 |
| Oxidoreductase Activity (GO:0016491) | | | | | | | | |
| 2.31 | MGG_09212 | DR | N | | oxidoreductase related to nitroreductase [Acinetobacter baumannii ACICU]. | | YP_001847897 | 3e-61 |
| 1.58 | MGG_01345* | DR | N | | gamma-cysteine synthetase regulatory subunit, putative [Aspergillus clavatus NRRL 1]. | | XP_001271381 | 3e-76 |
| 4.11 | MGG_01230* | DR | N | | succinate-semialdehyde dehydrogenase NADP+ [Chaetomium globosum CBS 148.51]. | | XP_001221129 | 0.0 |
| 8.01 | MGG_02069* | DR | Y | | glyoxalase family protein [Neosartorya fischeri NRRL 181]. | | XP_001266448 | 4e-27 |
| 1.54 | MGG_02612* | DR | N | | short-chain dehydrogenase [Aspergillus clavatus NRRL 1]. | | XP_001270195 | 4e-135 |
| 1.53 | MGG_14956* | DR | N | | cytochrome b5 reductase, putative [Aspergillus flavus NRRL 3357]. | | EED48869 | 2e-142 |
| 1.64 | MGG_01384* | DR |  | | amine oxidase, putative [Aspergillus flavus NRRL3357]. | | EED48219 | 3e-142 |
| 1.60 | MGG_02593* | DR | Y | | 2-nitropropane dioxygenase precursor [Neurospora crassa OR74A]. | | XP_957588 | 6e-66 |
| 1.58 | MGG_10053* | DR | N | | Pc13g06720 [Penicillium chrysogenum Wisconsin 54-1255]. | | CAP91741 | 5e-34 |
| 1.58 | MGG_05084* | DR | N | | NAD(P)H-dependent FMN reductase LOT6, putative [Aspergillus flavus NRRL3357]. | | EED54383 | 2e-34 |
| 1.58 | MGG_03919* | DR | Y | | glycine rich protein (het-COR) [Neurospora crassa]. | | CAD21080 | 0.0 |
| 1.58 | MGG_04240* | DR | Y | | salicylate 1-monooxygenase [Streptomyces pristinaespiralis ATCC 25486]. | | YP_002196185 | 3e-18 |
| 1.58 | MGG_13764 | DR | Y | | phenol oxidase A [Stachybotrys chartarum]. | | AAY23005 | 0.0 |
| 1.58 | MGG_00652 | DR | N | | salicylaldehyde dehydrogenase [Pyrenophora tritici-repentis Pt-1C-BFP]. | | XP_001940963 | 2e-145 |
| 2.00 | MGG_00461 | DR | N | | L-ascorbate oxidase [Laccaria bicolor S238N-H82]. | | XP_001875324 | 4e-40 |
| 1.58 | MGG_14550 | DR | N | | 3-oxoacyl-(acyl-carrier-protein) reductase [Pyrenophora tritici-repentis Pt-1C-BFP]. | | XP_001930997 | 1e-23 |
| 1.58 | MGG_06553* | DR | N | | retinol dehydrogenase 13 [Pyrenophora tritici-repentis Pt-1C-BFP]. | | XP_001940210 | 6e-70 |
| 1.58 | MGG_11542* | DR | N | | oxidoreductase, putative [Talaromyces stipitatus ATCC 10500]. | | EED22408 | 8e-81 |
| 1.58 | MGG_08293* | DR | N | | salicylate hydroxylase, putative [Penicillium marneffei ATCC 18224]. | | XP_002149772 | 2e-104 |
| 7.61 | MGG_13464* | DR | Y | | laccase [Gaeumannomyces graminis var. tritici]. | | CAD10749 | 0.0 |
| 2.29 | MGG_09715* | DR | N | | aldo-keto reductase (AKR13), puatative [Neosartorya fischeri NRRL 181]. | | XP_001262703 | 4e-101 |
| 7.56 | MGG_01655 | DR | Y | | copper radical oxidase [Phanerochaete chrysosporium]. | | ABD61575 | 0.0 |
| 7.18 | MGG_12228* | DR | N | | alcohol dehydrogenase, putative [Penicillium marneffei ATCC 18224]. | | XP_002144973 | 6e-42 |
| 6.23 | MGG_09328* | DR | N | | alcohol dehydrogenase, putative [Aspergillus flavus NRRL3357]. | | EED52013 | 3e-78 |
| 6.14 | MGG_13239* | DR | N | | linoleate diol synthase precursor [Magnaporthe grisea]. | | AAR21080 | 0 |
| 6.02 | MGG_05132* | DR | N | | zinc-binding oxidoreductase ToxD [Aspergillus fumigatus Af293]. | | XP_750901 | 2e-81 |
| 5.06 | MGG_01313* | DR | Y | | related to D-amino acid oxidase [Neurospora crassa]. | | CAE76563 | 6e-84 |
| 3.70 | MGG_04962* | DR | N | | Pc12g09170 [Penicillium chrysogenum Wisconsin 54-1255]. | | CAP80544 | 4e-124 |
| 3.58 | MGG_14232* | DR | N | | NADH-cytochrome B5 reductase, putative [Aspergillus flavusNRRL3357]. | | EED46514 | 0.0 |
| 3.40 | MGG_11608 | DR | Y | | laccase [Gaeumannomyces graminis var. tritici]. | | CAD10747 | 0.0 |
| 3.36 | MGG_04777 | DR | N | | antibiotic biosynthesis monooxygenase [Burkholderia phymatum STM815]. | | YP_001860150 | 1e-30 |
| 3.17 | MGG_08865* | DR | Y | | cytochrome P450, putative [Talaromyces stipitatus ATCC 10500]. | | EED14836 | 1e-133 |
| 3.17 | MGG_07982* | DR | N | | cytochrome P450 monoxygenase [Botryotinia fuckeliana]. | | CAH64679 | 7e-145 |
| 2.81 | MGG_05565 | DR | N | | glutathione S-transferase GST-6.0, putative [Aspergillus flavus NRRL3357]. | | EED51527 | 4e-22 |
| 2.81 | MGG_04404* | DR | Y | | pisatin demethylase [Pyrenophora tritici-repentis Pt-1C-BFP]. | | XP_001940087 | 2e-125 |
| 2.76 | MGG_01713* | DR | N | | norsolorinic acid reductase [Neurospora crassa OR74A]. | | XP_961973 | 3e-155 |
| 2.64 | MGG_05485* | DR | N | | amine oxidase, putative [Aspergillus flavus NRRL3357]. | | EED48219 | 8e-111 |
| 2.58 | MGG_00648* | DR | N | | PEX11 domain protein [Penicillium marneffei ATCC 18224]. | | XP_002145768 | 4e-13 |
| 2.32 | MGG_09139* | DR | Y | | laccase [Gaeumannomyces graminis var. tritici]. | | CAD10748 | 0.0 |
| 2.32 | MGG_08072* | DR | N | | related to cholesterol oxidase precursor [Neurospora crassa]. | | CAD21388 | 0.0 |
| 2.32 | MGG_07239* | DR | N | | Zbd1 [Fusarium oxysporum] | | ACA51797 | 2e-91 |
| 2.32 | MGG_01924* | DR | Y | | benzoate 4-monooxygenase cytochrome P450 [Pyrenophora tritici-repentis Pt-1C-BFP]. | | XP_001941086 | 7e-98 |
| 2.32 | MGG_07406 | DR | N | | benzoate 4-monooxygenase cytochrome P450, putative [Penicillium marneffei ATCC 18224]. | | XP_002151889 | 0.0 |
| 2.32 | MGG_15026* | DR | N | | glucose-methanol-choline (gmc) oxidoreductase, putative [Aspergillus flavus NRRL3357]. | | EED49404 | 6e-129 |
| 2.26 | MGG_01569* | DR | N | | minor allergen Alt a 7 [Neurospora crassa OR74A]. | | XP_965630 | 4e-85 |
| 2.00 | MGG_05215* | DR | N | | cytochrome P450 monooxygenase, putative [Neosartorya fischeri NRRL 181]. | | XP_001261614 | 8e-176 |
| 2.00 | MGG_12749* | DR | N | | glutathione-disulfide reductase (EC 1.8.1.7) [similarity] - Neurospora crassa. | | T51908 | 0.0 |
| 1.73 | MGG_13291* | DR | Y | | membrane copper amine oxidase, putative [Aspergillus flavus NRRL3357]. | | EED52335 | 3e-120 |
| 1.66 | MGG_04469* | DR | N | | cytochrome P450 78A3 [Pyrenophora tritici-repentis Pt-1C-BFP]. | | XP_001936907 | 2e-137 |
| 1.66 | MGG_06494* | DR | N | | short-chain dehydrogenase/reductase SDR [Actinobacillus succinogenes 130Z]. | | YP_001343445 | 8e-55 |
| 3.05 | MGG_05010* | UR | N | | NAD dependent epimerase/dehydratase, putative [Aspergillus flavus NRRL3357]. | | EED54484 | 5e-23 |
| 3.00 | MGG_14929* | UR | N | | 2-haloalkanoic acid dehalogenase, putative [Penicillium marneffei ATCC 18224]. | | XP_002147177 | 9e-78 |
| 2.81 | MGG_04975* | UR | N | | quinone oxidoreductase, putative [Talaromyces stipitatus ATCC 10500]. | | EED14784 | 2e-97 |
| 2.81 | MGG_01138 | UR | N | | O-methylsterigmatocystin oxidoreductase, putative [Penicillium marneffei ATCC 18224]. | | XP_002149781 | 1e-115 |
| 2.00 | MGG_10934* | UR | N | | antibiotic biosynthesis monooxygenase [Polaromonas sp. JS666]. | | YP_547214 | 0.002 |
| 2.00 | MGG_02902* | UR | N | | NAD-binding Rossmann fold oxidoreductase family protein [Neosartorya fischeri NRRL 181]. | | XP_001262160 | 3e-57 |
| 2.00 | MGG_07743 | UR | Y | | cytochrome P450 oxidoreductase GliC [Aspergillus flavus NRRL3357]. | | EED49633 | 5e-114 |
| 2.00 | MGG_14513* | UR | Y | | amine oxidase, flavin-containing superfamily [Aspergillus fumigatus Af293]. | | XP_748221 | 7e-38 |
| 1.93 | MGG_06179* | UR | Y | | apoptosis-inducing factor, putative [Penicillium marneffei ATCC 18224]. | | XP_002143222 | 5e-61 |
| 1.58 | MGG_11856* | UR | Y | | peroxidase, putative [Talaromyces stipitatus ATCC 10500]. | | EED21063 | 1e-40 |
| 1.58 | MGG_08730* | UR | Y | | flavin-containing monooxygenase, putative [Aspergillus fumigatus]. | | CAE47905 | 5e-54 |
| 1.58 | MGG_10012* | UR | N | | monooxygenase, putative [Talaromyces stipitatus ATCC 10500]. | | EED12123 | 1e-123 |
| 1.58 | MGG_07463* | UR | N | | putative D-galacturonic acid reductase [Hypocrea jecorina]. | | ABQ53585 | 9e-180 |
| 1.58 | MGG_02210 | UR | N | | Chain A, Crystal Structure Of A Trapped Phosphate-Intermediate In Vanadium Apochloroperoxidase Catalyzing A Dephosphorylation Reaction. | | 3BB0_A | 3e-146 |
| 1.58 | MGG_01923 | UR | N | | alcohol dehydrogenase, putative [Aspergillus flavus NRRL3357]. | | EED51852 | 1e-36 |
| 1.58 | MGG_02439* | UR | N | | oxidoreductase, 2-nitropropane dioxygenase family, putative [Aspergillus flavus NRRL3357]. | | EED53815 | 7e-114 |
| 1.58 | MGG_02737 | UR | N | | 2-dehydropantoate 2-reductase [Aspergillus fumigatus Af293]. | | XP_754967 | 4e-08 |
| 1.58 | MGG_07461* | UR | N | | FAD dependent oxidoreductase, putative [Neosartorya fischeri NRRL 181]. | | XP_001257626 | 3e-47 |
| Proteolysis (GO:0006508) | | | | | | | | |
| 4.78 | MGG_00311* | DR | Y | | acid protease [Pyrenophora tritici-repentis Pt-1C-BFP]. | | XP_001932923 | 4e-27 |
| 3.29 | MGG_11945* | DR | Y | | secreted aspartic proteinase [Hypocrea lixii]. | | ABK64120 | 1e-66 |
| 3.17 | MGG_02863 | DR | Y | | alkaline serine protease Alp1 [Aspergillus fumigatus Af293]. | | XP_751651 | 2e-102 |
| 3.17 | MGG_14860* | DR | Y | | alkaline serine protease [Aureobasidium pullulans]. | | ABP82774 | 5e-47 |
| 2.32 | MGG_02607* | DR | N | | dihydrofolate reductase [Aspergillus clavatus NRRL 1]. | | XP_001270681 | 3e-39 |
| 2.17 | MGG_03337 | DR | Y | | serine peptidase, putative [Aspergillus clavatus NRRL 1]. | | XP_001273182 | 9e-155 |
| 2.00 | MGG_07536 | DR | N | | aspartyl aminopeptidase [Neosartorya fischeri NRRL 181]. | | XP_001266136 | 4e-174 |
| 2.00 | MGG_03692* | DR | Y | | glutaminyl cyclase, putative [Aspergillus fumigatus A1163]. | | EDP50206 | 3e-115 |
| 2.00 | MGG_08854* | DR | N | | CaaX prenyl proteinase Rce1 [Talaromyces stipitatus ATCC 10500]. | | EED12504 | 6e-80 |
| 1.81 | MGG_14872* | DR | N | | calpain-5 [Pyrenophora tritici-repentis Pt-1C-BFP]. | | XP_001935550 | 5e-165 |
| 1.58 | MGG_10942 | DR | Y | | peptidase [Xanthomonas campestris pv. campestris str. B100]. | | YP_001903166 | 0.0 |
| 1.58 | MGG_02086* | DR | N | | proteasome component PRE3 precursor [Neurospora crassa OR74A]. | | XP_958142 | 3e-108 |
| 1.58 | MGG_12599* | DR | N | | acylase and diesterase protein [Agrobacterium vitis S4]. | | YP_002548930 | 3e-89 |
| 3 | MGG_02898* | UR | Y | | aspartic protease [Fusarium venenatum]. | | AAL69900 | 3e-111 |
| 2.58 | MGG_05151* | UR | N | | N-terminal acetyltransferase catalytic subunit Nat1, putative [Aspergillus flavus NRRL3357]. | | EED55521 | 0.0 |
| 2.58 | MGG_03637* | UR | N | | transcription initiation factor TFIID subunit TSM1/127kD, putative [Aspergillus flavus NRRL3357]. | | EED57202 | 0.0 |
| 2.32 | MGG_02529 | UR | Y | | putative lipoprotein [Solibacter usitatus Ellin6076]. | | YP_823487 | 1e-05 |
| 2.00 | MGG_06951* | UR | N | | probable zinc metallo-protease [Neurospora crassa]. | | CAC28689 | 0.0 |
| 2.00 | MGG_09246* | UR | Y | | subtilisin-like serine protease [Hypocrea lixii]. | | ABK64119 | 7e-112 |
| 1.58 | MGG_05684* | UR | N | | Xaa-Pro dipeptidase [Pyrenophora tritici-repentis Pt-1C-BFP]. | | XP_001940905 | 1e-111 |
| Carbohydrate Metabolism (GO:0005975) | | | | | | | | |
| 4.46 | MGG_02573 | DR | N | | glucan 1,3-beta-glucosidase [Sclerotinia sclerotiorum 1980]. | | XP_001593690 | 2e-156 |
| 1.58 | MGG_04718* | DR | N | | phosphoglycerate mutase [Pyrenophora tritici-repentis Pt-1C-BFP]. | | XP_001939050 | 1e-42 |
| 1.58 | MGG_10333 | DR | Y | | glycosyl hydrolase, family 18, putative [Neosartorya fischeri NRRL 181]. | | XP_001261449 | 2e-82 |
| 1.58 | MGG_01096* | DR | N | | Chain A, Glycoside Hydrolase Family 15 Glucoamylase From Hypocrea Jecorina. | | 2VN4_A | 0.0 |
| 1.58 | MGG_01264* | DR | N | | glycosyl hydrolase [Pyrenophora tritici-repentis Pt-1C-BFP]. | | XP_001932128 | 0.0 |
| 1.58 | MGG_09433* | DR | N | | glucanase, putative [Aspergillus clavatus NRRL 1]. | | XP_001273400 | 0.0 |
| 3.64 | MGG_14602* | DR | N | | endo-1,6-beta-D-glucanase [Neurospora crassa OR74A]. | | XP_957472 | 7e-155 |
| 2.7 | MGG_06631* | DR | Y | | 6-phosphogluconolactonase [Bacillus cereus B4264]. | | YP_002368087 | 2e-17 |
| 2.08 | MGG_09642* | DR | N | | alpha-amylase, putative [Aspergillus flavus NRRL3357]. | | EED55078 | 6e-127 |
| 2.00 | MGG_03643* | DR | N | | glucosyl/glucuronosyl transferase, putative [Talaromyces stipitatus ATCC 10500]. | | EED18718 | 0.0 |
| 2.00 | MGG_07306* | DR | N | | beta-1,3-endoglucanase [Colletotrichum gloeosporioides]. | | ACM90498 | 3e-73 |
| 1.72 | MGG_01732* | DR | Y | | 3-carboxymuconate cyclase [Pyrenophora tritici-repentis Pt-1C-BFP]. | | XP_001937682 | 2e-45 |
| 1.58 | MGG_06780* | DR | Y | | calx-beta domain protein [Clostridium perfringens E str. JGS1987]. | | ZP_02632076 | 2e-90 |
| 1.58 | MGG_01347 | DR | N | | 4-carboxymuconolactone decarboxylase, putative [Neosartorya fischeri NRRL 181]. | | XP_001258276 | 7e-54 |
| 1.58 | MGG_06636* | DR | N | | class II aldolase/adducin domain containing protein [Pyrenophora tritici-repentis Pt-1C-BFP]. | | XP_001940875 | 1e-72 |
| 1.58 | MGG_01314 | DR | Y | | related to alpha-mannosidase 1a [Neurospora crassa]. | | CAE76560 | 2e-161 |
| 1.58 | MGG_08424 | UR | Y | | endo-1,4-betaxylanase [Ascochyta pisi]. | | CAA93120 | 4e-85 |
| 3.09 | MGG_08123* | UR | N | | beta-glucosidase [Aspergillus clavatus NRRL 1]. | | XP_001274045 | 0.0 |
| 3.04 | MGG_01542* | UR | Y | | putative endo-beta-1,4-D-xylanase precursor [Magnaporthe grisea]. | | AAC41684 | 6e-166 |
| 3.00 | MGG_03245* | UR | Y | | aldose 1-epimerase [Pyrenophora tritici-repentis Pt-1C-BFP]. | | XP_001931792 | 1e-145 |
| 2.81 | MGG_09726* | UR | Y | | probable arabinogalactan endo-1, 4-beta-galactosidase [Neurosporacrassa]. | | CAE76342 | 2e-127 |
| 2.81 | MGG_02532 | UR | Y | | cellulase [Melanocarpus albomyces]. | | CAD56666 | 6e-145 |
| 2.58 | MGG_00882* | UR | N | | putative glycosyl hydrolase [Coccidioides posadasii]. | | ABA54914 | 0.0 |
| 2.53 | MGG_05809* | UR | Y | | vacuolar segregation protein (Pep7), putative [Aspergillus flavus NRRL3357]. | | EED54315 | 2e-170 |
| 2.41 | MGG_08583* | UR | N | | glycosyl hydrolase, putative [Neosartorya fischeri NRRL 181]. | | XP_001262303 | 6e-145 |
| 2.32 | MGG_12735* | UR | N | | alpha-mannosidase [Aspergillus flavus NRRL3357]. | | EED57992 | 0.0 |
| 2.32 | MGG_04534* | UR | Y | | chitinase [Chaetomium cupreum]. | | ABI48362 | 0.0 |
| 2.00 | MGG_00374* | UR | N | | 1,3-1,4-endoglucanase [Paenibacillus sp. f-40]. | | ABD94065 | 4e-08 |
| 2.00 | MGG_13777* | UR | Y | | endo-1,3(4)-beta-glucanase [Aspergillus fumigatus Af293]. | | XP_748630 | 8e-79 |
| 2.00 | MGG_01281* | UR | N | | glycerol kinase, putative [Talaromyces stipitatus ATCC 10500]. | | EED24342 | 0.0 |
| 1.87 | MGG_04015* | UR | Y | | cell wall glycosyl hydrolase Dfg5, putative [Talaromyces stipitatus ATCC 10500]. | | EED14334 | 1e-124 |
| 1.87 | MGG_03257* | UR | Y | | Alpha-L-fucosidase [Opitutus terrae PB90-1]. | | YP_001820098 | 1e-141 |
| 1.73 | MGG_03508* | UR | Y | | glycosyl hydrolase, putative [Neosartorya fischeri NRRL 181]. | | XP_001264012 | 0.0 |
| 1.58 | MGG_00319* | UR | Y | | Cel5b [Hypocrea jecorina]. | | AAP57754 | 2e-116 |
| 1.58 | MGG_01418 | UR | N | | glycosyl hydrolase [Metarhizium anisopliae]. | | ABD49724 | 1e-77 |
| 1.58 | MGG_13549 | UR | Y | | Putative esterase superfamily protein [Algoriphagus sp. PR1]. | | ZP_01718038 | 6e-38 |
| 1.58 | MGG_01389 | UR | N | | alpha-N-arabinofuranosidase [Pyrenophora tritici-repentis Pt-1C-BFP]. | | XP_001935209 | 0.0 |
| 1.58 | MGG_01247* | UR | N | | chitinase 1 precursor [Neurospora crassa OR74A]. | | XP_965309 | 3e-127 |
| 1.58 | MGG_01261 | UR | Y | | trehalase precursor [Pyrenophora tritici-repentis Pt-1C-BFP]. | | XP_001941464 | 0.0 |
| 1.58 | MGG_05489* | UR | Y | | endo-1,3-beta-glucanase Engl1 [Penicillium marneffei ATCC 18224]. | | XP_002150278 | 3e-179 |
| 1.58 | MGG_09601* | UR | N | | glycosyl hydrolase, putative [Aspergillus flavus NRRL3357]. | | EED57325 | 0.0 |
| Lipid Metabolism (GO:0006629) | | | | | | | | |
| 2.80 | MGG_01369* | DR | N | | hormone-sensitive lipase [Magnaporthe grisea]. | | ABG79927 | 3e-108 |
| 2.90 | MGG_12304* | DR | N | | proteinase, putative [Penicillium marneffei ATCC 18224]. | | XP_002152181 | 2e-63 |
| 1.58 | MGG_06254 | DR | N | | Chain A, Crystal Structure Of A Type Iii Polyketide Synthase Pksiiinc From Neurospora Crassa. | | 3E1H_A | 7e-75 |
| 2.80 | MGG_10397* | DR | N | | Pc12g14690 [Penicillium chrysogenum Wisconsin 54-1255]. | | CAP81096 | 2e-73 |
| 2.58 | MGG_04956* | DR | N | | long-chain-fatty-acid-CoA ligase 1 [Pyrenophora tritici-repentis Pt-1C-BFP]. | | XP_001941525 | 5e-171 |
| 2.58 | MGG_04194* | DR | Y | | acetyl esterase [Hypocrea jecorina]. | | ABI34466 | 4e-93 |
| 2.32 | MGG_04867 | DR | N | | lipase [Aspergillus fumigatus Af293]. | | XP_747967 | 5e-111 |
| 2.16 | MGG_04935* | DR | N | | integral membrane protein [Pyrenophora tritici-repentis Pt-1C-BFP]. | | XP_001935475 | 3e-33 |
| 1.74 | MGG_06610 | DR | Y | | lipase [Streptomyces clavuligerus ATCC 27064]. | | YP_002193099 | 1e-19 |
| 1.58 | MGG_02543* | DR | Y | | FG-GAP repeat domain-containing protein [Streptomyces sviceus ATCC 29083]. | | YP_002205181 | 2e-22 |
| 1.58 | MGG_03369* | DR | Y | | temperature-induced lipocalin [Medicago truncatula]. | | ABB02396 | 1e-12 |
| 2.80 | MGG_09314* | UR | Y | | lipolytic enzyme [Pyrenophora tritici-repentis Pt-1C-BFP]. | | XP_001939963 | 1e-114 |
| 2.80 | MGG_04869* | UR | N | | candidate lipase/esterase enzyme from carbohydrate esterase family CE10 [Postia placenta Mad-698-R]. | | EED82748 | 3e-47 |
| 2.58 | MGG_02913* | UR | N | | lipase, putative [Aspergillus clavatus NRRL 1]. | | XP_001270261 | 4e-141 |
| 2.58 | MGG_10202* | UR | Y | | 3-oxoacyl-[acyl-carrier-protein]-synthase [Neurospora crassa OR74A]. | | XP_955799 | 5e-174 |
| 2.32 | MGG_08416* | UR | Y | | lipase 1 precursor [Pyrenophora tritici-repentis Pt-1C-BFP]. | | XP_001931605 | 2e-176 |
| 2.32 | MGG_05879* | UR | Y | | esterase, putative [Aspergillus fumigatus A1163]. | | EDP55661 | 3e-18 |
| 2.32 | MGG_12999* | UR | N | | acetoacetyl-CoA synthase [Aspergillus fumigatus Af293]. | | XP_747283 | 0.0 |
| 2.00 | MGG_02987 | UR | Y | | acetylcholinesterase precursor [Pyrenophora tritici-repentis Pt-1C-BFP]. | | XP_001936657 | 2e-170 |
| 2.00 | MGG_13767* | UR | N | | polyketide synthase, putative [Talaromyces stipitatus ATCC 10500]. | | EED14251 | 0.0 |
| 2.00 | MGG_15272* | UR | N | | polyketide synthase [Monascus pilosus]. | | ABA02239 | 0.0 |
| 1.73 | MGG_05619* | UR | N | | dihydroceramidase [Pyrenophora tritici-repentis Pt-1C-BFP]. | | XP_001931181 | 6e-67 |
| 1.66 | MGG_11317* | UR | N | | long chain fatty alcohol oxidase [Pyrenophora tritici-repentis Pt-1C-BFP]. | | XP_001940486 | 3e-175 |
| 1.58 | MGG_08550* | UR | Y | | endoglucanase E precursor [Pyrenophora tritici-repentis Pt-1C-BFP]. | | XP_001936152 | 7e-79 |
| 1.58 | MGG_03444* | UR | N | | short-chain dehydrogenase, putative [Penicillium marneffei ATCC18224]. | | XP_002144653 | 2e-68 |
| 1.58 | MGG_01156 | UR | N | | phospholipid metabolism enzyme regulator, putative [Aspergillus fumigatus A1163]. | | EDP54353 | 4e-71 |
| 1.58 | MGG_08281* | UR |  | | non-reducing polyketide synthase [Hypomyces subiculosus]. | | ACD39753 | 0.0 |
| 1.58 | MGG_03998* | UR | N | | Phospholipase/Carboxylesterase superfamily [Talaromyces stipitatus ATCC 10500]. | | EED18082 | 4e-34 |
| 1.58 | MGG_14244* | UR | Y | | extracellular lipase [Gibberella zeae]. | | AAQ23181 | 6e-45 |
| 1.58 | MGG_00744 | UR | N | | importin 11, putative [Aspergillus clavatus NRRL 1]. | | XP_001269796 | 0.0 |
| Cell development (GO:0007275) | | | | | | | | |
| 5.02 | MGG_00097* | DR | N | NADP-dependent glycerol-2-dehydrogenase [Hypocrea jecorina]. | | | ABD83953 | 1e-136 |
| 1.66 | MGG_10856* | DR | N | Pc21g16860 [Penicillium chrysogenum Wisconsin 54-1255]. | | | CAP96583 | 2e-08 |
| 1.62 | MGG_15429 | DR | N | PREDICTED: similar to ZK1055.7, partial [Hydra magnipapillata]. | | | XP_002168864 | 9e-18 |
| 8.14 | MGG_05232* | DR | Y | IgE-binding protein [Aspergillus fumigatus Af293]. | | | XP_731512 | 1e-18 |
| 1.58 | MGG_06888* | DR | N | glutamine synthetase [Magnaporthe grisea 70-15]. | | | XP_001402868 | 5e-168 |
| 1.58 | MGG_08113 | DR | Y | non-classical export protein Nce102, putative [Aspergillus flavus NRRL3357]. | | | EED51983 | 4e-04 |
| 1.58 | MGG_08003* | DR | N | UbiA prenyltransferase [Chloroflexus sp. Y-400-fl]. | | | YP_002568665 | 5e-21 |
| 1.58 | MGG_03494* | DR | N | aminotransferase, class III [Talaromyces stipitatus ATCC 10500]. | | | EED22339 | 4e-149 |
| 1.53 | MGG_14917* | DR | Y | ring canal kelch-like protein [Xanthomonas campestris pv. campestris str. ATCC 33913]. | | | AAM43333 | 2e-15 |
| 4.91 | MGG_15157* | DR | N | acetyltransferase, GNAT family family [Aspergillus fumigatus | | | XP_747370 | 3e-20 |
| 4.86 | MGG_10533* | DR | Y | agmatinase [Aspergillus fumigatus Af293]. | | | XP_753336 | 0.0 |
| 4.84 | MGG_03823* | DR | N | NADH oxidase [Pyrenophora tritici-repentis Pt-1C-BFP]. | | | XP_001934295 | 3e-131 |
| 4.25 | MGG_04736* | DR | N | Pc13g11240 [Penicillium chrysogenum Wisconsin 54-1255]. | | | CAP92193 | 8e-40 |
| 4.17 | MGG_00099* | DR | N | CDP-alcohol phosphatidyltransferase [Botryotinia fuckeliana B05.10]. | | | XP_001556772 | 0.0 |
| 3.86 | MGG_07747 | DR | N | DUF74 domain protein [Penicillium marneffei ATCC 18224]. | | | XP_002145737 | 2e-39 |
| 3.82 | MGG_10274* | DR | N | NADPH-dependent 1-acyl dihydroxyacetone phosphate reductase, putative [Aspergillus clavatus NRRL 1]. | | | XP_001272279 | 8e-70 |
| 3.70 | MGG_15113 | DR | N | aldo/keto reductase, putative [Talaromyces stipitatus ATCC 10500]. | | | EED22571 | 8e-74 |
| 3.7 | MGG_12982* | DR | Y | short-chain dehydrogenase, putative [Talaromyces stipitatus ATCC 10500]. | | | EED21267 | 1e-69 |
| 3.46 | MGG_05228* | DR | N | Pc18g02430 [Penicillium chrysogenum Wisconsin 54-1255]. | | | CAP94467 | 0.002 |
| 3.27 | MGG_03915 | DR | N | pyridoxamine phosphate oxidase family protein [Aspergillus fumigatus Af293]. | | | XP_751027 | 1e-49 |
| 3.17 | MGG_05368 | DR | N | ankyrin repeat-containing protein, putative [Penicillium marneffei ATCC 18224]. | | | XP_002145339 | 8e-45 |
| 2.84 | MGG_00270* | DR | N | zinc-containing alcohol dehydrogenase, putative [Neosartory fischeri NRRL 181]. | | | XP_001267198 | 1e-135 |
| 2.81 | MGG_09261* | DR | N | cell surface protein, putative [Talaromyces stipitatus ATCC 10500]. | | | EED20992 | 1e-21 |
| 2.81 | MGG_11237 | DR | N | Pc22g12430 [Penicillium chrysogenum Wisconsin 54-1255]. | | | CAP98531 | 6e-18 |
| 2.81 | MGG_03038* | DR | N | Pc20g14000 [Penicillium chrysogenum Wisconsin 54-1255]. | | | CAP86729 | 8e-42 |
| 2.70 | MGG_02992 | DR | N | hypothetical protein MPER_02872 [Moniliophthora perniciosa FA553]. | | | EEB97743 | 2e-20 |
| 2.58 | MGG_03689* | DR | Y | short-chain dehydrogenase, putative [Talaromyces stipitatus ATCC 10500]. | | | EED15254 | 2e-57 |
| 2.42 | MGG_13290* | DR | N | Alpha/beta hydrolase [Fulvimarina pelagi HTCC2506]. | | | ZP_01438122 | 1e-27 |
| 2.41 | MGG_06840* | DR | Y | putative d-galactose 1-dehydrogenase protein [Fulvimarina pelagi HTCC2506]. | | | ZP_01438058 | 0.53 |
| 2.32 | MGG_05124 | DR | Y | Pc13g14080 [Penicillium chrysogenum Wisconsin 54-1255]. | | | CAP92477 | 1e-09 |
| 2.32 | MGG_06865 | DR | N | hypothetical protein NCU07978 [Neurospora crassa OR74A]. | | | XP_962648 | 6e-12 |
| 2.32 | MGG_05813* | DR | N | predicted protein [Chaetomium globosum CBS 148.51]. | | | XP_001224204 | 3e-04 |
| 2.32 | MGG_02574 | DR | N | NCP1 pseudogene [Cryptococcus neoformans var. neoformans]. | | | AAN75605 | 3e-83 |
| 2.32 | MGG_13261 | DR | N | extracellular serine-rich protein, putative [Penicillium marneffei ATCC 18224]. | | | XP_002149600 | 2e-10 |
| 2.27 | MGG_05980* | DR | N | pyridoxine biosynthesis protein [Aspergillus fumigatus Af293]. | | | XP_753827 | 1e-131 |
| 2.20 | MGG_06461* | DR | N | Pc06g00910 [Penicillium chrysogenum Wisconsin 54-1255]. | | | CAP79084 | 2e-113 |
| 2.13 | MGG_03464* | DR | N | Pc13g02500 [Penicillium chrysogenum Wisconsin 54-1255]. | | | CAP91319 | 2e-66 |
| 2.07 | MGG_05732 | DR | N | integral membrane protein, putative [Penicillium marneffei ATCC18224]. | | | XP_002148425 | 2e-55 |
| 2.05 | MGG_07464* | DR | N | HypA-like protein, putative [Neosartorya fischeri NRRL 181]. | | | XP_001266201 | 8e-84 |
| 2.00 | MGG_03980 | DR | N | Pc20g12450 [Penicillium chrysogenum Wisconsin 54-1255]. | | | CAP86574 | 1e-10 |
| 2.00 | MGG_11148* | DR | N | Pc22g00510 [Penicillium chrysogenum Wisconsin 54-1255]. | | | CAP97339 | 5e-21 |
| 2.00 | MGG_12377* | DR | N | putative phytochrome-like histidine kinase PHY1p [Gibberella moniliformis]. | | | AAR30124 | 0.0 |
| 2.00 | MGG_02387* | DR | N | Leucine Rich Repeat domain protein [Penicillium marneffei ATCC 18224]. | | | XP_002144303 | 3e-10 |
| 1.88 | MGG_10704* | DR | N | GNAT family acetyltransferase, putative [Neosartorya fischeri NRRL 181]. | | | XP_001259077 | 8e-26 |
| 1.88 | MGG_04682* | DR | N | integral membrane protein, putative [Neosartorya fischeri NRRL 181]. | | | XP_001264627 | 6e-32 |
| 1.81 | MGG_00312* | DR | N | 2-hydroxyacid dehydrogenase, putative [Penicillium marneffei ATCC 18224]. | | | XP_002149100 | 2e-77 |
| 1.79 | MGG_08050* | DR | N | Pc13g06390 [Penicillium chrysogenum Wisconsin 54-1255]. | | | CAP91708 | 6e-35 |
| 1.75 | MGG_05139* | DR | Y | histidine acid phosphatase, putative [Aspergillus clavatus NRRL 1]. | | | XP_001276358 | 1e-99 |
| 1.75 | MGG_00738* | DR | N | quinone oxidoreductase [Aspergillus fumigatus Af293]. | | | XP_001265442 | 2e-107 |
| 1.67 | MGG_06601* | DR | Y | Pc16g13370 [Penicillium chrysogenum Wisconsin 54-1255]. | | | CAP94007 | 1e-10 |
| 2.80 | MGG_09260* | UR | N | integral membrane protein [Aspergillus fumigatus Af293]. | | | XP_753773 | 8e-32 |
| 2.16 | MGG_07099* | UR | N | DEAD box RNA helicase HelA, putative [Neosartorya fischeri NRR 181]. | | | XP_001264188 | 5e-119 |
| 2.00 | MGG_05054 | UR | Y | PREDICTED: similar to CG6180-PA [Apis mellifera]. | | | XP_001122227 | 1e-09 |
| 2.32 | MGG_05049* | UR | N | Pc21g19990 [Penicillium chrysogenum Wisconsin 54-1255]. | | | CAP96896 | 2e-122 |
| 2.16 | MGG_09925* | UR | N | RecName: Full=Mediator of RNA polymerase II transcription subunit 14; AltName: Full=Mediator complex subunit 14. | | | Q7S154 | 0.0 |
| 2.00 | MGG_15036* | UR | N | nuclear distribution protein RO10, putative [Talaromyces stipitatus ATCC 10500]. | | | EED17600 | 1e-23 |
| 1.83 | MGG_09955* | UR | N | RNA binding protein, putative [Talaromyces stipitatus ATCC 10500]. | | | EED21084 | 3e-26 |
| 1.81 | MGG_10526* | UR | Y | RING zinc finger protein, putative [Aspergillus fumigatus A1163]. | | | EDP56629 | 0.0 |
| 1.81 | MGG_10456* | UR | Y | hypersensitive response-inducing protein [Ophiostoma ulmi]. | | | ABK76310 | 2e-10 |
| 1.73 | MGG_01085* | UR | Y | ThiJ/PfpI family protein [Talaromyces stipitatus ATCC 10500]. | | | EED16209 | 1e-38 |
| 1.58 | MGG_02454 | UR | N | MYND domain protein (SamB), putative [Aspergillus fumigatus A1163]. | | | EDP51961 | 7e-169 |
| 1.58 | MGG_10859 | UR | N | fatty acid oxygenase, putative [Aspergillus flavus NRRL3357]. | | | EED45847 | 0.0 |
| 1.58 | MGG_08867 | UR | N | ribosome assembly protein Noc2, putative [Aspergillus fumigatus A1163]. | | | EDP56846 | 0.0 |
| 1.58 | MGG_08893* | UR | N | Pc12g10860 [Penicillium chrysogenum Wisconsin 54-1255]. | | | CAP80713 | 5e-04 |
| Cell Wall & Surface (GO:0044036) | | | | | | | | |
| 8.54 | MGG_14966 | DR | N | | chitin deacetylase, putative [Aspergillus clavatus NRRL 1]. | | XP_001268141 | 2e-61 |
| 3.25 | MGG_03529* | DR | Y | | integral membrane protein [Aspergillus clavatus NRRL 1]. | | XP_001268327 | 5e-140 |
| 2.32 | MGG_07522* | DR | N | | Pc12g11890 [Penicillium chrysogenum Wisconsin 54-1255]. | | CAP80816 | 5e-33 |
| 1.92 | MGG_10661* | DR | Y | | surface protein 1 [Glomerella lindemuthiana]. | | CAL38822 | 7e-08 |
| 1.58 | MGG_12468 | DR | Y | | chitinase 3 [Coccidioides posadasii]. | | ABA38735 | 5e-06 |
| 1.80 | MGG_07571* | DR | Y | | LysM domain protein [Neosartorya fischeri NRRL 181]. | | XP_001257349 | 5e-31 |
| 1.51 | MGG_14095* | DR | Y | | TPA: putative cuticle protein [Bombyx mori]. | | FAA00649 | 4e-27 |
| 3.81 | MGG_04348 | UR | Y | | PEL_EMENI Pectate lyase precursor [Aspergillus nidulans FGSC A4]. | | XP_658345 | 6e-118 |
| 2.32 | MGG_11774* | UR | N | | endoglucanase, putative [Neosartorya fischeri NRRL 181]. | | XP_001259157 | 7e-45 |
| 2.00 | MGG_03970 | UR | N | | 1,3-beta-glucan biosynthesis protein [Aspergillus fumigatus Af293]. | | XP_754055 | 4e-138 |
| 2.00 | MGG_06694* | UR | N | | DUF250 domain membrane protein [Talaromyces stipitatus ATCC 10500]. | | EED19866 | 3e-139 |
| 1.58 | MGG_01801 | UR | N | | actin-related protein RO7 [Neurospora crassa]. | | AAF74760 | 3e-126 |
| 1.58 | MGG_10690 | UR | N | | actin-like protein 2 [Sclerotinia sclerotiorum 1980]. | | XP_001598984 | 0.0 |
| 1.58 | MGG_03879 | UR | N | | ARP3_NEUCR Actin-like protein 3 [Gibberella zeae PH-1]. | | XP_391032 | 0.0 |
| Electron transport Metabolism (GO:0006118) | | | | | | | | |
| 3.17 | MGG_02818 | DR | Y | FAD binding domain protein [Neosartorya fischeri NRRL 181]. | | | XP_001262117 | 6e-79 |
| 2.58 | MGG_04911 | DR | N | short-chain dehydrogenases/reductase, putative [Talaromyces stipitatus ATCC 10500]. | | | EED13656 | 2e-61 |
| 1.58 | MGG_09816 | DR | N | TRI15 [Fusarium sporotrichioides]. | | | AAG47841 | 1e-41 |
| 1.58 | MGG_07856 | DR | Y | PDA-like cytochrome P450 monooxygenase , putative [Aspergillus fumigatus A1163]. | | | EDP49520 | 2e-147 |
| 1.58 | MGG_04144* | DR | N | sulfite reductase [Neurospora crassa]. | | | AAL14263 | 0.0 |
| 2.32 | MGG_10727* | DR | N | Pc21g14660 [Penicillium chrysogenum Wisconsin 54-1255]. | | | CAP96363 | 1e-11 |
| 2.07 | MGG_04236* | DR | N | thioredoxin [Botryotinia fuckeliana B05.10]. | | | XP_001546606 | 1e-31 |
| 2.02 | MGG_01708* | DR | N | AN1 zinc finger protein [Aspergillus flavus NRRL3357]. | | | EED44789 | 3e-69 |
| 2.00 | MGG_09602 | DR | Y | membrane copper amine oxidase, putative [Penicillium marneffei ATCC 18224]. | | | XP_002145636 | 1e-122 |
| 6.17 | MGG_10907* | DR | N | FAD-dependent oxygenase, putative [Aspergillus flavus NRRL3357]. | | | EED53712 | 2e-74 |
| 6.17 | MGG_13929* | DR | N | isoflavone reductase family protein [Penicillium marneffei ATCC 18224]. | | | XP_002145668 | 3e-78 |
| 2.00 | MGG_14110* | DR | N | Pc13g10860 [Penicillium chrysogenum Wisconsin 54-1255]. | | | CAP92155 | 5e-13 |
| 1.79 | MGG_05447* | DR | N | glutaredoxin Grx1, putative [Penicillium marneffei ATCC 18224]. | | | XP_002144051 | 4e-33 |
| 1.58 | MGG_08500* | DR | N | benzoate 4-monooxygenase cytochrome P450, putative [Aspergillus flavus NRRL3357]. | | | EED47415 | 5e-123 |
| 1.58 | MGG_03059* | DR | N | Pc21g00720 [Penicillium chrysogenum Wisconsin 54-1255]. | | | CAP94969 | 2e-22 |
| 1.58 | MGG_06872* | DR | Y | related to cytochrome P450 3A7 [Neurospora crassa]. | | | CAC18306 | 9e-122 |
| 1.58 | MGG_06761* | DR | N | FAD binding domain protein [Neosartorya fischeri NRRL 181]. | | | XP_001261662 | 5e-44 |
| 2.90 | MGG_06653 | UR | Y | extracellular serine-rich protein, putative [Penicillium marneffei ATCC 18224]. | | | XP_002149600 | 3e-19 |
| 2.81 | MGG_09198 | UR | N | cytochrome P450 [Aspergillus clavatus NRRL 1]. | | | XP_001274956 | 9e-84 |
| 2.32 | MGG_08487 | UR | Y | cellobiose dehydrogenase, putative [Aspergillus flavus NRRL3357]. | | | EED49453 | 5e-158 |
| 2.32 | MGG_05964* | UR | N | 2Fe-2S iron-sulfur cluster binding domain protein [Aspergillus fumigatus Af293]. | | | XP_747954 | 2e-45 |
| 2.00 | MGG_06325 | UR | N | vacuolar protein sorting protein DigA [Neosartorya fischeri NRRL 181]. | | | XP_001265993 | 0.0 |
| 2.00 | MGG_11346* | UR | N | unnamed protein product [Podospora anserina]. | | | XP_001908413 | 9e-45 |
| 2.00 | MGG_06662* | UR | Y | FAD dependent oxidoreductase, putative [Aspergillus clavatus NRRL 1]. | | | XP_001272391 | 3e-91 |
| 2.00 | MGG_01941 | UR | N | FAD binding domain protein [Aspergillus clavatus NRRL 1]. | | | XP_001275696 | 1e-57 |
| 2.00 | MGG_00973* | UR | Y | FAD dependent oxidoreductase, putative [Aspergillus clavatus NRRL1]. | | | XP_001272391 | 1e-116 |
| 1.81 | MGG_11229* | UR | N | CCR4-NOT core complex subunit Not4, putative [Aspergillus flavus NRRL3357]. | | | EED48636 | 1e-139 |
| 1.70 | MGG_06198* | UR | N | LIM domain containing protein [Pyrenophora tritici-repentis Pt-1C-BFP]. | | | XP_001935779 | 9e-47 |
| 1.58 | MGG_06489* | UR | N | oxidoreductase, zinc-binding [Talaromyces stipitatus ATCC 10500]. | | | EED19975 | 2e-106 |
| 1.58 | MGG_15003* | UR | N | 37S ribosomal protein Rsm22 [Aspergillus flavus NRRL3357]. | | | EED52633 | 2e-136 |
| 1.58 | MGG_11239* | UR | N | RING-10 protein [Pyrenophora tritici-repentis Pt-1C-BFP]. | | | XP_001932983 | 3e-142 |
| Metabolism (GO:0008152) | | | | | | | | |
| 2.58 | MGG_10023* | DR | Y | short-chain dehydrogenase, putative [Penicillium marneffei ATCC 18224]. | | | XP_002150110 | 4e-37 |
| 2.58 | MGG_08923* | DR | N | threonine synthase [Neurospora crassa OR74A]. | | | XP_956711 | 0.0 |
| 2.39 | MGG_08201* | DR | N | DUF636 domain protein [Aspergillus flavus NRRL3357]. | | | EED54328 | 6e-67 |
| 2.38 | MGG_07309* | DR | N | enoyl-CoA hydratase/isomerase family protein [Aspergillus clavatus NRRL 1]. | | | XP_001271402 | 3e-101 |
| 2.32 | MGG_13017* | DR | N | Amt4 [Alternaria alternata]. | | | BAF76162 | 2e-31 |
| 2.32 | MGG_03414* | DR | Y | short chain dehydrogenase/reductase family [Penicillium marneffei ATCC 18224]. | | | XP_002147732 | 3e-55 |
| 2.23 | MGG_10503* | DR | N | mannitol-1-phosphate 5-dehydrogenase [Neurospora crassa OR74A]. | | | XP_962476 | 3e-177 |
| 2.20 | MGG_02616* | DR | N | mitochondrial 2-methylisocitrate lyase [Neurospora crassa OR74A]. | | | XP_965075 | 0.0 |
| 2.17 | MGG_04895 | DR | N | isocitrate lyase [Neurospora crassa OR74A]. | | | XP_960854 | 0.0 |
| 2.07 | MGG_13518* | DR | N | short chain dehydrogenase, putative [Penicillium marneffei ATCC 18224]. | | | XP_002150505 | 3e-106 |
| 2.04 | MGG_14767* | DR | N | related to AM-toxin synthetase (AMT) [Neurospora crassa]. | | | CAD70509 | 0.0 |
| 2.00 | MGG_07491* | DR | N | short-chain dehydrogenase, putative [Aspergillus clavatus NRRL 1]. | | | XP_001268764 | 3e-85 |
| 2.00 | MGG_12726* | DR | N | PhzF family phenazine biosynthesis protein [Burkholderia ambifaria MC40-6]. | | | YP_001811321 | 1e-49 |
| 2.00 | MGG_05159* | DR | N | dihydrodipicolinate synthetase family protein [Talaromyces stipitatus ATCC 10500]. | | | EED19711 | 1e-115 |
| 2.00 | MGG_00065* | DR | N | haloacid dehalogenase-like hydrolase, putative [Aspergillus clavatus NRRL 1]. | | | XP_001273020 | 8e-29 |
| 2.00 | MGG_10560* | DR | N | N-acetyltransferase family protein, putative [Aspergillus flavus NRRL3357]. | | | EED55421 | 1e-41 |
| 1.93 | MGG_08455* | DR | Y | FAD binding domain containing protein [Pyrenophora tritici-repentis Pt-1C-BFP]. | | | XP_001932676 | 2e-102 |
| 1.93 | MGG_00550* | DR | N | 2-keto-4-pentenoate hydratase [Sclerotinia sclerotiorum 1980]. | | | XP_001594644 | 2e-98 |
| 1,82 | MGG_00805 | DR | N | kynurenine 3-monooxygenase [Aspergillus fumigatus Af293]. | | | XP_750627 | 5e-158 |
| 1.79 | MGG_05915 | DR | N | fumarylacetoacetate hydrolase [Neosartorya fischeri NRRL 181]. | | | XP_001257289 | 9e-107 |
| 1.74 | MGG_05022* | DR | N | NmrA-like family protein [Aspergillus flavus NRRL3357]. | | | EED52322 | 4e-78 |
| 1.73 | MGG_06466* | DR | Y | cysteine synthase [Chaetomium globosum CBS 148.51]. | | | XP_001221807 | 0.0 |
| 1.71 | MGG_08288 | DR | N | long-chain-fatty-acid-CoA ligase [Aspergillus fumigatus Af293]. | | | XP_753087 | 0.0 |
| 1.65 | MGG_04243* | DR | Y | peptide methionine sulphoxide [Botryotinia fuckeliana B05.10]. | | | XP_001560063 | 3e-79 |
| 1.58 | MGG_08900* | DR | N | WD40 domain protein [Talaromyces stipitatus ATCC 10500]. | | | EED21447 | 3e-36 |
| 1.58 | MGG_06642 | DR | N | phosphoglycerate mutase family domain protein [Aspergillus clavatus NRRL 1]. | | | XP_001271005 | 7e-121 |
| 1.58 | MGG_04386* | DR | N | urea amidolyase [Pichia stipitis CBS 6054]. | | | XP_001387473 | 0.0 |
| 1.58 | MGG_15167 | DR | Y | succinyl-CoA ligase subunit alpha [Pyrenophora tritici-repentis Pt-1C-BFP]. | | | XP_001939199 | 3e-95 |
| 1.58 | MGG_07763* | DR | N | alkaline phosphatase, putative [Aspergillus flavus NRRL3357]. | | | EED53656 | 2e-104 |
| 6.49 | MGG_08297 | DR | N | NADPH dehydrogenase [Botryotinia fuckeliana B05.10]. | | | XP_001558622 | 6e-115 |
| 6.04 | MGG_08989* | DR | N | short chain dehydrogenase (AtsC), putative [Aspergillus flavus NRRL3357]. | | | EED51702 | 3e-62 |
| 5.52 | MGG_12983* | DR | N | short-chain dehydrogenase, putative [Talaromyces stipitatus ATCC 10500] | | | EED21264 | 5e-57 |
| 5.43 | MGG_12714* | DR | N | oxidoreductase CipA-like, putative [Aspergillus flavus NRRL3357]. | | | EED54115 | 3e-36 |
| 4.84 | MGG_03823 | DR | N | NADH oxidase [Pyrenophora tritici-repentis Pt-1C-BFP]. | | | XP_001934295 | 3e-131 |
| 4.67 | MGG_07261* | DR | Y | 2-nitropropane dioxygenase precursor [Neurospora crassa OR74A]. | | | XP_957588 | 3e-94 |
| 3.53 | MGG_07009 | DR | Y | short chain dehydrogenase [Botryotinia fuckeliana B05.10]. | | | XP_001556323 | 4e-80 |
| 3.46 | MGG_00402* | DR | N | short-chain dehydrogenase, putative [Penicillium marneffei ATCC 18224]. | | | XP_002149745 | 6e-76 |
| 3.46 | MGG_10631 | DR | Y | glycoside hydrolase family 24 protein [Laccaria bicolor S238N-H82]. | | | XP_001887554 | 3e-26 |
| 3.32 | MGG_10714 | DR | N | cyanide hydratase [Fusarium solani]. | | | CAM82815 | 1e-174 |
| 3.32 | MGG_03807* | DR | N | putative outer membrane protein precursor [Pseudomonas aeruginosa UCBPP-PA14]. | | | YP_790336 | 0.13 |
| 3.09 | MGG_05759* | DR | N | related to hxB protein [Neurospora crassa]. | | | CAB97294 | 1e-178 |
| 3.00 | MGG_00156* | DR | N | NmrA family transcriptional regulator, putative [Penicillium marneffei ATCC 18224]. | | | XP_002148633 | 1e-69 |
| 2.82 | MGG_09345* | DR | N | cysteine desulfurylase, putative [Aspergillus flavus NRRL3357]. | | | EED48908 | 5e-95 |
| 2.82 | MGG_04569* | DR | N | NADH-dependent flavin oxidoreductase-like protein [Magnaporthe grisea]. | | | AAX07634 | 0.0 |
| 2.81 | MGG_05101* | DR | N | NmrA-like family protein [Neosartorya fischeri NRRL 181]. | | | XP_001260891 | 1e-46 |
| 2.62 | MGG_11927* | DR | N | 3-oxoacyl-acyl-carrier-protein reductase [Botryotinia fuckeliana B05.10]. | | | XP_001547173 | 9e-80 |
| 2.32 | MGG_02095* | DR | N | isoflavone reductase family protein (CipA), putative [Neosartorya fischeri NRRL 181]. | | | XP_001262282 | 1e-37 |
| 2.32 | MGG_01553 | DR | N | 3-isopropylmalate dehydratase [Neurospora crassa OR74A]. | | | XP_957462 | 0.0 |
| 2.32 | MGG_09785* | DR | N | short-chain dehydrogenase, putative [Aspergillus flavus NRRL3357]. | | | EED51778 | 3e-68 |
| 2.28 | MGG_01606* | DR | N | methylmalonate-semialdehyde dehydrogenase, mitochondrial precursor [Neurospora crassa OR74A]. | | | XP_964701 | 0.0 |
| 5.67 | MGG_10910* | DR | N | Pc22g19590 [Penicillium chrysogenum Wisconsin 54-1255]. | | | CAP99247 | 8e-44 |
| 5.61 | MGG_11468 | DR | N | NmrA-like family protein [Aspergillus fumigatus Af293]. | | | XP_731527 | 8e-56 |
| 2.80 | MGG_08047* | UR | N | glycerophosphoryl diester phosphodiesterase family protein [Aspergillus fumigatus Af293]. | | | XP_751103 | 6e-130 |
| 2.58 | MGG_01537* | UR | N | bifunctional pyrimidine biosynthesis protein (PyrABCN), putative [Penicillium marneffei ATCC 18224]. | | | XP_002150090 | 0.0 |
| 2.12 | MGG_00183* | UR | N | mRNA cleavage factor complex component Pcf11, putative [Penicillium marneffei ATCC 18224]. | | | XP_002149182 | 4e-116 |
| 2.00 | MGG_06388 | UR | N | SNF2-family ATP dependent chromatin remodeling factor snf21 [Neurospora crassa OR74A]. | | | XP_957128 | 0.0 |
| 2.00 | MGG_02919* | UR | N | Pc20g09890 [Penicillium chrysogenum Wisconsin 54-1255]. | | | CAP86318 | 7e-41 |
| 2.00 | MGG_00919 | UR | N | GMP synthase [Talaromyces stipitatus ATCC 10500]. | | | EED14082 | 0.0 |
| 2.00 | MGG_04900* | UR | Y | protein tyrosine phosphatase [Metarhizium anisopliae var. acridum]. | | | ABC01064 | 0.0 |
| 2.00 | MGG_11223 | UR | N | enoyl-CoA hydratase/isomerase family protein [Aspergillus clavatus NRRL 1]. | | | XP_001275158 | 3e-58 |
| 1.84 | MGG_05596 | UR | N | protein N-terminal amidase [Botryotinia fuckeliana B05.10]. | | | XP_001546472 | 9e-55 |
| 1.58 | MGG_05975* | UR | N | UDP-glucuronosyl/UDP-glucosyltransferase [Mycobacterium vanbaalenii PYR-1]. | | | YP_955665 | 1e-32 |
| 1.58 | MGG_09068* | UR | N | short-chain dehydrogenase/reductase, putative [Aspergillus clavatus NRRL 1]. | | | XP_001268505 | 4e-68 |
| 1.58 | MGG_05798* | UR | Y | cutinase precursor [Pyrenophora tritici-repentis Pt-1C-BFP]. | | | XP_001935104 | 5e-49 |
| 1.58 | MGG_10687* | UR | N | related to DCG1 protein [Neurospora crassa]. | | | CAD71132 | 2e-57 |
| 1.58 | MGG_00335 | UR | N | mitochondrial cytochrome b2, putative [Aspergillus clavatus NRRL 1]. | | | XP_001269442 | 0.0 |
| 1.58 | MGG_08842 | UR | N | AGR323Cp [Ashbya gossypii ATCC 10895]. | | | NP_986989 | 3e-47 |
| 1.58 | MGG_05349* | UR | N | vacuolar protein sorting protein Vps66, putative [Talaromyces stipitatus ATCC 10500]. | | | EED19815 | 4e-83 |
| 1.58 | MGG_02831 | UR | N | inositol 5-phosphatase, putative [Penicillium marneffei ATCC 18224]. | | | XP_002145497 | 8e-51 |
| Cellular Metabolic Process (GO:0044237) | | | | | | | | |
| 5.61 | MGG_09945* | DR | Y | | cytochrome P450 46A1 [Pyrenophora tritici-repentis Pt-1C-BFP]. | | XP_001938675 | 1e-100 |
| 1.58 | MGG_14526* | DR | N | | iron-sulfur cluster assembly associated protein Nar1, putative [Talaromyces stipitatus ATCC 10500]. | | EED24251 | 8e-168 |
| 1.58 | MGG_07084* | DR | N | | 3' exoribonuclease family protein [Pyrenophora tritici-repentis Pt-1C-BFP]. | | XP_001939168 | 2e-76 |
| 1.58 | MGG_06517* | DR | N | | pre-rRNA processing protein, putative [Neosartorya fischeri NRRL 181]. | | XP_001260105 | 1e-18 |
| 1.58 | MGG_11533* | DR | N | | RNase III [Pelobacter carbinolicus DSM 2380]. | | YP_356819 | 1e-04 |
| 1.58 | MGG_07175* | DR | N | | KOW motif domain protein [Talaromyces stipitatus ATCC 10500]. | | EED23826 | 2e-31 |
| 1.58 | MGG_05888* | DR | N | | 5-methyltetrahydropteroyltriglutamate-homocysteine methyltransferase [Pyrenophora tritici-repentis Pt-1C-BFP]. | | XP_001938845 | 5e-120 |
| 1.58 | MGG_12098 | DR | N | | orotidine-5'-phosphate decarboxylase (EC 4.1.1.23) - fungus (Acremonium lolii). | | JC4104 | 2e-136 |
| 1.58 | MGG_06921 | DR | N | | alpha/beta fold family hydrolase, putative [Neosartorya fischeri NRRL 181]. | | XP_001265407 | 4e-85 |
| 1.58 | MGG_02429* | DR | N | | RING-2 [Gibberella zeae]. | | ABG78604 | 1e-51 |
| 1.58 | MGG_05976* | DR | N | | beta-lactamase family protein [Neosartorya fischeri NRRL 181]. | | XP_001262163 | 1e-54 |
| 5.17 | MGG_01662* | DR | Y | | 4-aminobutyrate aminotransferase [Neurospora crassa OR74A]. | | XP_959026 | 0.0 |
| 3.95 | MGG_02072 | DR | N | | amino-acid permease inda1 [Pyrenophora tritici-repentis Pt-1C-BFP]. | | XP_001939063 | 0.0 |
| 3.70 | MGG_00203* | DR | N | | Pc06g02130 [Penicillium chrysogenum Wisconsin 54-1255]. | | CAP79206 | 6e-05 |
| 3.39 | MGG_09297* | DR | N | | acohol dehydrogenase domain protein [Klebsiella pneumoniae 342]. | | YP_002239015 | 8e-44 |
| 3.32 | MGG_12895* | DR | N | | thiamine-4 [Neurospora crassa]. | | BAA21049 | 3e-168 |
| 3.17 | MGG_01353* | DR | N | | cytochrome P450 monooxygenase [Pyrenophora tritici-repentis Pt-1C-BFP]. | | XP_001932099 | 7e-104 |
| 3.00 | MGG_06782* | DR | N | | neutral amino acid permease, putative [Talaromyces stipitatus ATCC 10500]. | | EED24120 | 6e-169 |
| 2.81 | MGG_11636 | DR | N | | ankyrin repeat domain containing protein [Pyrenophora tritici-repentis Pt-1C-BFP]. | | XP_001939919 | 9e-12 |
| 2.80 | MGG_01506 | DR | N | | 6-phosphogluconate dehydrogenase 2 [Pyrenophora tritici-repentis Pt-1C-BFP]. | | XP_001941482 | 3e-88 |
| 2.75 | MGG_05123 | DR | N | | Pc13g02230 [Penicillium chrysogenum Wisconsin 54-1255]. | | CAP91292 | 2e-53 |
| 2.70 | MGG_12844* | DR | N | | zeta toxin family protein [Frankia sp. EAN1pec]. | | YP_001510623 | 2e-59 |
| 2.70 | MGG_05981* | DR | N | | pyridoxine [Talaromyces stipitatus ATCC 10500]. | | EED21888 | 9e-68 |
| 2.58 | MGG_04248* | DR | N | | oligopeptide transporter [Laccaria bicolor S238N-H82]. | | XP_001881083 | 5e-133 |
| 2.58 | MGG_02657* | DR | N | | cytochrome c oxidase-assembly factor cox-16, mitochondrial precursor [Pyrenophora tritici-repentis Pt-1C-BFP]. | | XP_001938493 | 4e-33 |
| 2.58 | MGG_09131 | DR | N | | alpha/beta hydrolase fold [Frankia sp. EAN1pec]. | | YP_001506354 | 8e-25 |
| 2.46 | MGG_09333* | DR | Y | | nucleoside-diphosphate-sugar epimerases, putative [Neosartorya fischeri NRRL 181]. | | XP_001267578 | 2e-67 |
| 2.32 | MGG_01322* | DR | N | | threonyl-tRNA synthetase, mitochondrial precursor [Neurospora crassa OR74A]. | | XP_964988 | 4e-177 |
| 2.20 | MGG_07317 | DR | N | | glutamate-cysteine ligase catalytic subunit [Neurospora crassa OR74A]. | | XP_961523 | 0.0 |
| 2.17 | MGG_04026 | DR | N | | related to translation initiation factor IF-3 [Neurospora crassa]. | | CAE76140 | 7e-18 |
| 2.17 | MGG_05125 | DR | N | | Ankyrin repeat protein [Neosartorya fischeri NRRL 181]. | | XP_001258750 | 0.0 |
| 2.13 | MGG_02709* | DR | N | | Pc22g23680 [Penicillium chrysogenum Wisconsin 54-1255]. | | CAP99656 | 1e-43 |
| 2.11 | MGG_05066 | DR | N | | HpcH/HpaI aldolase [Burkholderia phymatum STM815]. | | YP_001862904 | 1e-36 |
| 2.00 | MGG_14907* | DR | N | | glutamine-dependent NAD(+) synthetase [Neosartorya fischeri NRRL 181]. | | XP_001266199 | 0.0 |
| 2.00 | MGG_10497* | DR | N | | protein bli-3 [Neurospora crassa OR74A]. | | XP_962121 | 3e-77 |
| 2.00 | MGG_10297* | DR | N | | C6 transcription factor, putative [Talaromyces stipitatus ATCC 10500]. | | EED11978 | 1e-12 |
| 2.00 | MGG_07014 | DR | N | | DNA repair protein RAD16 [Neurospora crassa OR74A]. | | XP_961377 | 0.0 |
| 2.00 | MGG_04410* | DR | N | | origin recognition complex subunit [Pyrenophora tritici-repentis Pt-1C-BFP]. | | XP_001937982 | 5e-95 |
| 2.00 | MGG_05122 | DR | N | | kinesin, putative [Talaromyces stipitatus ATCC 10500]. | | EED13534 | 2e-63 |
| 2.00 | MGG_09508* | DR | N | | 30S ribosomal protein S7 [Aspergillus fumigatus Af293]. | | XP_750175 | 2e-41 |
| 1.99 | MGG_00212 | DR | N | | superoxide dismutase, mitochondrial precursor [Neurospora crassa OR74A]. | | XP_959485 | 2e-88 |
| 1.87 | MGG_08486 | DR | Y | | beta-lactamase, putative [Talaromyces stipitatus ATCC 10500]. | | EED16113 | 3e-64 |
| 1.87 | MGG_06485* | DR | N | | aminomethyl transferase, putative [Talaromyces stipitatus ATCC 10500]. | | EED22102 | 8e-82 |
| 1.83 | MGG_13815* | DR | N | | ATP synthase subunit 9 [Pyrenophora tritici-repentis Pt-1C-BFP]. | | XP_001935361 | 2e-31 |
| 1.78 | MGG_06454* | DR | Y | | glutathione synthetase large chain [Pyrenophora tritici-repentis Pt-1C-BFP]. | | XP_001931457 | 6e-143 |
| 1.71 | MGG_06683 | DR | N | | 3' exoribonuclease family protein (Rrp42), putative [Penicillium marneffei ATCC 18224]. | | XP_002147094 | 4e-96 |
| 1.71 | MGG_01826* | DR | N | | cell cycle control protein (Cwf26) [Aspergillus fumigatus Af293]. | | XP_746502 | 2e-57 |
| 1.69 | MGG_07161* | DR | N | | related to pyruvate dehydrogenase kinase isoform 2, mitochondrial [Neurospora crassa]. | | CAB91764 | 0.0 |
| 1.58 | MGG_00567* | DR | N | | NACHT and TPR domain protein [Neosartorya fischeri NRRL 181]. | | XP_001261617 | 3e-67 |
| 1.58 | MGG_03869* | DR | N | | glutamate decarboxylase, putative [Talaromyces stipitatus ATCC 10500]. | | EED19925 | 2e-171 |
| 2.81 | MGG_00630* | UR | N | | related to ATP-dependent DNA helicase [Neurospora crassa]. | | CAE85555 | 0.0 |
| 2.58 | MGG_05990 | UR | N | | mRNA capping nucleoside-triphosphatase [Aspergillus fumigatus Af293]. | | XP_751975 | 2e-52 |
| 2.58 | MGG_08122* | UR | N | | DNA replication licensing factor mcm2 [Neurospora crassa OR74A]. | | XP_960973 | 0.0 |
| 2.46 | MGG_01893 | UR | N | | ribonuclease P complex subunit Pop4, putative [Aspergillus clavatus NRRL 1]. | | XP_001274409 | 8e-49 |
| 2.00 | MGG_07148* | UR | N | | ribose-phosphate pyrophosphokinase II [Neurospora crassa OR74A]. | | XP_962610 | 1e-143 |
| 2.00 | MGG_04981* | UR | N | | DUF28 domain protein [Aspergillus clavatus NRRL 1]. | | XP_00127013 | 2e-37 |
| 2.00 | MGG_01541 | UR | N | | RNase3 domain protein [Neosartorya fischeri NRRL 181]. | | XP_001259517 | 0.0 |
| 2.00 | MGG_05260* | UR | N | | rRNA biogenesis protein RRP5, putative [Neosartorya fischeri NRRL 181] | | XP_001261068 | 0.0 |
| 2.00 | MGG_04882 | UR | N | | Cdc48-dependent protein degradation adaptor protein (Shp1), putative [Penicillium marneffei ATCC 18224]. | | XP_002147779 | 1e-74 |
| 2.00 | MGG_01512* | UR | N | | ATP-dependent DNA helicase II, 70 kDa subunit [Sordaria macrospora]. | | CAJ41424 | 0.0 |
| 1.92 | MGG_03002 | UR | N | | cryptochrome DASH [Pyrenophora tritici-repentis Pt-1C-BFP]. | | XP_001936482 | 2e-133 |
| 1.88 | MGG_03329* | UR | N | | small heat shock protein [Hypocrea lixii]. | | AAX55622 | 3e-37 |
| 1.83 | MGG_00881* | UR | N | | DNA primase small subunit [Neurospora crassa OR74A]. | | XP_959645 | 0.0 |
| 1.81 | MGG_12831 | UR | N | | mRNA capping enzyme alpha subunit, putative [Neosartorya fischeri NRRL 181]. | | XP_001260200 | 4e-102 |
| 1.81 | MGG_09170 | UR | N | | PAB1 binding protein (Pbp1), putative [Aspergillus flavus NRRL3357]. | | EED54897 | 5e-77 |
| 1.81 | MGG_07121* | UR | N | | DNA mismatch repair protein Mlh1, putative [Penicillium marneffei ATCC 18224]. | | XP_002144802 | 0.0 |
| 1.63 | MGG_08049 | UR | N | | DEAD box RNA helicase (Hca4), putative [Penicillium marneffei ATCC 18224]. | | XP_002150065 | 0.0 |
| 1.58 | MGG_09461* | UR | N | | UV-endonuclease UVE-1 [Neosartorya fischeri NRRL 181]. | | XP_001258214 | 5e-153 |
| 1.58 | MGG_04145* | UR | N | | chitin synthase B [Aspergillus flavus NRRL3357]. | | EED44774 | 0.0 |
| 1.58 | MGG_11267 | UR | N | | folylpolyglutamate synthetase; FPGS [Neurospora crassa]. | | AAB61730 | 4e-153 |
| 1.58 | MGG_14004* | UR | N | | ribonuclease H [Aspergillus fumigatus Af293]. | | XP_750465 | 2e-67 |
| 1.58 | MGG_14265* | UR | Y | | nucleoside-diphosphate-sugar epimerase, putative [Aspergillus flavus NRRL3357]. | | EED54448 | 7e-92 |
| 1.58 | MGG_01378* | UR | N | | Pc22g11510 [Penicillium chrysogenum Wisconsin 54-1255]. | | CAP98439 | 2e-178 |
| 1.58 | MGG_08527* | UR | Y | | nucleoside-diphosphate-sugar epimerase, putative [Neosartorya fischeri NRRL 181]. | | XP_001263186 | 4e-87 |
| 1.58 | MGG_09235 | UR | N | | DnaJ domain protein [Aspergillus fumigatus A1163]. | | EDP48071 | 6e-152 |
| 1.58 | MGG_05146 | UR | N | | nucleolar protein NOP52 variant [Pyrenophora tritici-repentis Pt-1C-BFP]. | | XP_001931285 | 1e-35 |
| 1.58 | MGG_07502 | UR | Y | | DnaJ domain protein, putative [Penicillium marneffei ATCC 18224]. | | XP_002146527 | 3e-139 |
| 1.58 | MGG_04310* | UR | N | | 60S ribosomal protein L6, putative [Talaromyces stipitatus ATCC 10500]. | | EED23403 | 3e-68 |
| 1.58 | MGG_05025* | UR | Y | | long-chain fatty acid transport protein 3 [Pyrenophora tritici-repentis Pt-1C-BFP]. | | XP_001935974 | 2e-174 |
| 1.58 | MGG_08260* | UR | N | | nucleoside-diphosphate-sugar epimerase [Stenotrophomonas sp. SKA14]. | | YP_002705583 | 0.002 |
| 1.56 | MGG_10180* | UR | N | | cell cycle control protein (Cwf22) [Aspergillus fumigatus Af293]. | | XP_750051 | 0.0 |
| 1.53 | MGG_02853* | UR | Y | | probable nuclease S1 precursor [Neurospora crassa]. | | CAD79647 | 5e-95 |
| 1.53 | MGG_08876* | UR | N | | PREDICTED: similar to U2-associated SR140 protein [Ciona intestinalis]. | | XP_002129060 | 1e-16 |
| Protein modification (GO:0006464) | | | | | | | | |
| 1.58 | MGG_06679* | DR | N | | ubiquitin conjugating enzyme [Aspergillus fumigatus Af293]. | | XP_751438 | 2e-164 |
| 2.32 | MGG_04517 | UR | N | | Sin3 complex subunit (Stb2) [Aspergillus fumigatus Af293]. | | XP_750196 | 2e-164 |
| 2.12 | MGG_00183 | UR |  | | MAP kinase kinase kinase SskB, putative [Penicillium marneffei ATCC 18224]. | | XP_002151422 | 0.0 |
| 1.81 | MGG_13931* | UR | N | | serine/threonine-protein kinase prk1 [Neurospora crassa OR74A]. | | XP_957397 | 1e-155 |
| 1.58 | MGG_09322 | UR | N | | tripeptidyl peptidase precursor [Beauveria bassiana]. | | AAQ89573 | 5e-63 |
| 1.58 | MGG_06696 | UR | N | | sensor histidine kinase/response regulator, putative [Aspergillus flavus NRRL3357]. | | EED57269 | 0.0 |
| 1.58 | MGG_01393 | UR | N | | Mitogen-activated protein kinase kinase kinase, putative [Ricinus communis]. | | EEF49249 | 3e-09 |
| 1.58 | MGG_09565 | UR | N | | MAP kinase 1 [Gaeumannomyces graminis]. | | AAG44657 | 0.0 |
| 1.58 | MGG_03488* | UR | N | | cell cycle protein kinase, putative [Aspergillus clavatus NRRL 1]. | | XP_001270578 | 3e-135 |
| 1.58 | MGG_11140* | UR | N | | Protein kinase domain-containing protein [Talaromyces stipitatus ATCC 10500]. | | EED20735 | 9e-155 |
| 1.58 | MGG_05312* | UR | N | | protein kinase (Lkh1), putative [Neosartorya fischeri NRRL 181]. | | XP_001264078 | 0.0 |
| 1.58 | MGG_01074* | UR | N | | WD repeat containing protein 48 [Pyrenophora tritici-repentis Pt-1C-BFP]. | | XP_001931049 | 0.0 |
| 1.58 | MGG_10826* | UR | N | | checkpoint protein kinase, putative [Penicillium marneffei ATCC 18224]. | | XP_002153511 | 1e-136 |
| Protein synthesis (GO:0006412) | | | | | | | | |
| 1.58 | MGG_02895 | DR | N | | tyrosine-protein phosphatase precursor, putative [Talaromyces stipitatus ATCC 10500]. | | EED17608 | 5e-29 |
| Response to stress (GO:0006950) | | | | | | | | |
| 4.22 | MGG_08200 | DR | Y | | L-ascorbate oxidase [Laccaria bicolor S238N-H82]. | | XP_001875324 | 7e-67 |
| 2.87 | MGG_06747* | DR | Y | | glutathione S-transferase [Sclerotinia sclerotiorum 1980]. | | XP_001591749 | 3e-98 |
| 2.58 | MGG_07790 | DR | Y | | ligninase H2 precursor [Pyrenophora tritici-repentis Pt-1C-BFP]. | | XP_001933374 | 4e-96 |
| 2.17 | MGG_03896 | DR | N | | alpha/beta hydrolase fold [Mycobacterium vanbaalenii PYR-1]. | | YP_952969 | 5e-15 |
| 2 | MGG_01612* | DR | Y | | hypothetical protein CHGG_01482 [Chaetomium globosum CBS 148.51]. | | XP_001220703 | 3e-12 |
| 2.58 | MGG_00194 | UR | Y | | stress response protein Rds1, putative [Aspergillus flavus NRRL3357]. | | EED49274 | 8e-154 |
| 2.46 | MGG_06033 | UR | Y | | mucin family signaling protein Msb2, putative [Aspergillus flavus NRRL3357]. | | EED44743 | 1e-25 |
| 2.46 | MGG_15090* | UR | N | | Pc22g14370 [Penicillium chrysogenum Wisconsin 54-1255]. | | CAP98725 | 0.0 |
| 1.58 | MGG_09507* | UR | N | | universal stress protein family domain protein [Aspergillus flavus NRRL3357]. | | EED50881 | 2e-73 |
|  |  |  |  | |  | |  |  |
| Transcription regulation (GO:0006350) | | | | | | | | |
| -5.78 | MGG_04213 | DR | N | | C6 transcription factor, putative [Talaromyces stipitatus ATCC 0500]. | | EED18596 | 4e-14 |
| Ap1 | MGG_12814* | DR | Y | | Chap1 [Cochliobolus heterostrophus]. | | AAS64313 | 1e-75 |
| 4.21 | MGG_10717* | DR | N | | C6 transcription factor, putative [Aspergillus fumigatus A1163]. | | EDP54187 | 3e-90 |
| 3.80 | MGG_10422* | DR | N | | C6 transcription factor OefC [Aspergillus flavus NRRL3357]. | | EED57819 | 8e-54 |
| 3.08 | MGG_14586* | DR | N | | C6 zinc finger domain containing protein [Pyrenophora tritici-repentis Pt-1C-BFP]. | | XP_001933074 | 8e-65 |
| 2.83 | MGG_09200* | DR | N | | C2H2 finger domain protein, putative [Neosartorya fischeri NRRL 181]. | | XP_001262050 | 7e-109 |
| 2.58 | MGG_11764* | DR | N | | C6 transcription factor RosA-like, putative [Aspergillus flavus NRRL3357]. | | EED57649 | 1e-93 |
| 2.58 | MGG_01518* | DR | N | | pathway-specific nitrogen regulator [Tolypocladium inflatum]. | | CAB71797 | 0.0 |
| 2.32 | MGG_07800 | DR | N | | C6 zinc finger domain protein [Neosartorya fischeri NRRL 181]. | | XP_001258875 | 1e-33 |
| 2.32 | MGG_10694* | DR | N | | C6 transcription factor, putative [Aspergillus clavatus NRRL 1]. | | XP_001273840 | 2e-89 |
| 2 | MGG_06416* | DR | N | | C6 finger domain protein, putative [Penicillium marneffei ATCC 18224]. | | XP_002146019 | 4e-31 |
| 2 | MGG_02447 | DR | N | | TFIIIC transcription initiation factor complex subunits Tfc3 [Aspergillus flavus NRRL3357]. | | EED47698 | 8e-40 |
| 2 | MGG_00590 | DR | N | | related to histone transcription regulator [Neurospora crassa]. | | CAE85596 | 0.0 |
| 1.95 | MGG_04970* | DR | N | | C6 zinc finger domain protein [Aspergillus clavatus NRRL 1]. | | XP_001273498 | 7e-09 |
| 1.85 | MGG_13994* | DR | N | | C6 transcription factor, putative [Neosartorya fischeri NRRL 181]. | | XP_001263245 | 2e-39 |
| 1.81 | MGG_07339* | DR | N | | C2H2 type zinc finger domain protein [Aspergillus clavatus NRRL 1]. | | XP_001276534 | 9e-28 |
| 1.78 | MGG_09312 | DR | N | | C6 finger domain protein, putative [Aspergillus clavatus NRRL 1]. | | XP_001269337 | 2e-19 |
| 1.78 | MGG_04024* | DR | N | | Pc22g16800 [Penicillium chrysogenum Wisconsin 54-1255]. | | CAP98968 | 5e-04 |
| 1.73 | MGG_08185 | DR | N | | C6 transcription factor, putative [Aspergillus flavus NRRL3357]. | | EED47932 | 4e-30 |
| 1.58 | MGG_06690* | DR | N | | RNA polymerase TFIIH complex subunit Ssl1 [Aspergillus fumigatus Af293]. | | XP_001481497 | 8e-145 |
| 1.58 | MGG_01641* | DR | N | | chromatin-associated protein [Enterocytozoon bieneusi H348]. | | EED41801 | 4e-14 |
| 1.58 | MGG_00856* | DR | N | | CP2 transcription factor, putative [Talaromyces stipitatus ATCC 10500]. | | EED19919 | 0.0 |
| 1.58 | MGG_05959* | DR | N | | bZIP transcription factor (HapX), putative [Aspergillus fumigatus A1163]. | | EDP51238 | 5e-48 |
| 1.58 | MGG_01779* | DR | N | | C6 zinc finger domain protein [Aspergillus clavatus NRRL 1]. | | XP_001273498 | 5e-12 |
| 1.58 | MGG_12473* | DR | N | | nitrogen metabolite repression-(nmr)-responsible protein [Gibberella fujikuroi]. | | XP_370145 | 1e-146 |
| 1.54 | MGG_04933* | DR | N | | C6 transcription factor [Aspergillus fumigatus Af293]. | | XP_751289 | 1e-18 |
| 1.52 | MGG_00342 | DR | N | | bZIP-type transcription factor [Emericella nidulans]. | | CAM35586 | 4e-51 |
| 1.58 | MGG_07437* | UR | N | | homeobox transcription factor, putative [Neosartorya fischeri NRRL 181]. | | XP_001262370 | 8e-43 |
| 2.81 | MGG_08314* | UR | N | | C6 transcription factor [Aspergillus fumigatus Af293]. | | XP_749075 | 0.0 |
| 2.58 | MGG_09118* | UR | N | | C6 transcription factor (Mut3), putative [Talaromyces stipitatus ATCC 10500]. | | EED19981 | 9e-108 |
| 2.58 | MGG_06422* | UR | N | | forkhead box protein L2 [Aspergillus terreus NIH2624]. | | XP_001218091 | 3e-67 |
| 2.32 | MGG_12636* | UR | N | | RNA polymerase II mediator complex component SRB4, putative [Neosartorya fischeri NRRL 181]. | | XP_001267533 | 3e-56 |
| 2.32 | MGG_03877* | UR | N | | transcription factor TFIIH subunit Tfb4, putative [Neosartorya fischeri NRRL 181]. | | XP_001259536 | 2e-82 |
| 2.32 | MGG_08875* | UR | N | | copper-sensing transcription factor [Epichloe festucae]. | | ACB30150 | 4e-74 |
| 2.14 | MGG_15048 | UR | N | | SIR2 family histone deacetylase, putative [Penicillium marneffei ATCC 18224]. | | XP_002144717 | 1e-78 |
| 2.07 | MGG_00692 | UR | N | | APSES transcription factor [Glomerella cingulata]. | | ABQ43358 | 7e-143 |
| 2.00 | MGG_15093* | UR | N | | C6 transcription factor, putative [Aspergillus clavatus NRRL 1]. | | XP_001268124 | 2e-12 |
| 2.00 | MGG_13498* | UR | N | | transcription factor (Sin3), putative [Aspergillus fumigatus A1163]. | | EDP48756 | 0.0 |
| 2.00 | MGG_00184* | UR | N | | homeobox transcription factor, putative [Penicillium marneffei ATCC 18224]. | | XP_002151547 | 5e-15 |
| 2.00 | MGG_03485* | UR | N | | RNA Polymerase II CTD phosphatase Fcp1 [Aspergillus fumigatus Af293]. | | XP_754478 | 4e-121 |
| 2.00 | MGG_00885* | UR | Y | | transcription initiation factor TFIID subunit 12 [Pyrenophora tritici-repentis Pt-1C-BFP]. | | XP_001932341 | 6e-44 |
| 2.00 | MGG_04518* | UR | N | | regulator of gluconeogenesis Rmd5 [Aspergillus fumigatus Af293]. | | XP_001481705 | 1e-96 |
| 2.00 | MGG_01558 | UR | N | | PHD finger and SET domain protein, putative [Aspergillus flavus NRRL3357]. | | EED50643 | 5e-98 |
| 2.00 | MGG_04758* | UR | N | | bZIP transcription factor, putative [Aspergillus flavus NRRL3357]. | | EED57155 | 2e-64 |
| 2.00 | MGG_08295* | UR | N | | C6 transcription factor, putative [Aspergillus clavatus NRRL 1]. | | XP_001267937 | 7e-30 |
| 1.81 | MGG_05343* | UR | N | | C6 zinc finger domain containing protein [Pyrenophora tritici-repentis Pt-1C-BFP]. | | XP_001937314 | 1e-84 |
| 1.81 | MGG_07149* | UR | N | | C6 finger domain protein, putative [Aspergillus clavatus NRRL 1]. | | XP_001271908 | 3e-06 |
| 1.77 | MGG_08917* | UR | N | | C6 transcription factor, putative [Aspergillus clavatus NRRL 1]. | | XP_001268221 | 3e-74 |
| 1.58 | MGG_01653* | UR | N | | probable transcription factor HAP3 [Neurospora crassa]. | | CAE76299 | 2e-74 |
| 1.58 | MGG_14133* | UR | N | | mitochondrial DNA-directed RNA polymerase, putative [Talaromyces stipitatus ATCC 10500]. | | EED13045 | 0.0 |
| 1.58 | MGG_08212 | UR | N | | putative bZip transcription factor [Claviceps purpurea]. | | CAD21519 | 7e-126 |
| 1.58 | MGG_09186 | UR | N | | related to regulator of deoxyribodipyrimidine photo-lyase PHR1 [Neurospora crassa]. | | CAD21068 | 0.0 |
| 1.58 | MGG_04571 | UR | N | | C6 transcription factor, putative [Penicillium marneffei ATCC 18224]. | | XP_002153387 | 5e-30 |
| 1.58 | MGG_01887 | UR | N | | C6 zinc finger domain containing protein [Pyrenophora tritici-repentis Pt-1C-BFP]. | | XP_001939276 | 7e-132 |
| 1.58 | MGG_05306* | UR | N | | bZIP transcription factor (MeaB), putative [Aspergillus flavus NRRL3357]. | | EED55907 | 5e-59 |
| 1.58 | MGG_12268* | UR | N | | transcription initiation protein spt5 [Aspergillus fumigatus A1163]. | | EDP50228 | 0.0 |
| 1.58 | MGG_00632 | UR | N | | cell differentiation protein rcd1 [Neurospora crassa OR74A]. | | XP_960336 | 5e-169 |
| 1.58 | MGG_09562* | UR | N | | related to negative acting factor [Neurospora crassa]. | | CAB91400 | 9e-115 |
| 1.58 | MGG_09275* | UR | N | | Rho GTPase activator (Bem3), putative [Talaromyces stipitatus ATCC 10500]. | | EED20360 | 0.0 |
| 1.58 | MGG_07140* | UR | N | | C2H2 transcription factor (RfeC) [Aspergillus fumigatus Af293]. | | XP_755388 | 1e-61 |
| 1.58 | MGG_05939* | UR | N | | C6 transcription factor [Aspergillus fumigatus Af293]. | | XP_749478 | 5e-130 |
| 1.58 | MGG_02880* | UR | N | | C6 transcription factor, putative [Penicillium marneffei ATCC 18224]. | | XP_002145389 | 2e-94 |
| 1.50 | MGG_10674* | UR | N | | RNA polymerase I subunit Rpa43, putative [Aspergillus clavatus NRRL 1]. | | XP_001272336 | 1e-29 |
| Transport (GO:0006810) | | | | | | | | |
| 8.32 | MGG_03212* | DR | N | | methyltransferase, putative [Aspergillus flavus NRRL3357] | | EED53205 | 5e-74 |
| 1.62 | MGG_08002 | DR | N | | uracil permease [Neurospora crassa OR74A]. | | XP_962492 | 0.0 |
| 1.62 | MGG_05880* | DR | N | | glycerol uptake facilitator, putative [Talaromyces stipitatus ATCC 10500]. | | EED18600 | 2e-93 |
| 1.58 | MGG_11454* | DR | N | | calcium/proton exchanger [Neurospora crassa]. | | AAC08353 | 2e-145 |
| 1.58 | MGG_13332* | DR | N | | aquaporin, putative [Aspergillus flavus NRRL3357]. | | EED52460 | 6e-55 |
| 1.58 | MGG_07957* | DR | N | | uracil permease [Neurospora crassa OR74A]. | | XP_962492 | 8e-94 |
| 1.58 | MGG_02204* | DR | N | | MFS transporter Seo1, putative [Aspergillus flavus NRRL3357]. | | EED47464 | 2e-144 |
| 1.58 | MGG_04968* | DR | N | | cation diffusion facilitator 1 [Pyrenophora tritici-repentis Pt-1C-BFP]. | | XP_001934369 | 7e-153 |
| 1.58 | MGG_10702 | DR | N | | monocarboxylate permease homologue, mch4 [Neosartorya fischeri NRRL 181]. | | XP_001265376 | 3e-103 |
| 1.58 | MGG_04852* | DR | N | | P-type ATPase [Schizosaccharomyces pombe]. | | XP_001713045 | 0.0 |
| 1.58 | MGG_09520 | DR | N | | ABC transporter [Aspergillus fumigatus Af293]. | | XP_747569 | 2e-102 |
| 1.58 | MGG_01480 | DR | N | | related to large-conductance mechanosensitive channel [Neurospora crassa]. | | CAB91331 | 7e-40 |
| 1.58 | MGG_00111* | DR | N | | P-type ATPase [Magnaporthe grisea]. | | AAL01053 | 0.0 |
| 1.58 | MGG_03843* | DR | N | | MFS multidrug transporter, putative [Aspergillus fumigatus A1163]. | | EDP55980 | 8e-162 |
| 6.19 | MGG_14928* | DR | N | | MFS transporter, putative [Talaromyces stipitatus ATCC 10500]. | | EED14213 | 6e-67 |
| 5.55 | MGG_07606* | DR | N | | dicarboxylic amino acid permease [Aspergillus terreus NIH2624]. | | XP_001218343 | 1e-138 |
| 3.81 | MGG_04153* | DR | N | | florfenicol exporter, putative [Talaromyces stipitatus ATCC 10500]. | | EED20513 | 2e-67 |
| 3.46 | MGG_04357* | DR | N | | transporter, putative [Candida dubliniensis CD36]. | | CAX45167 | 2e-80 |
| 3.32 | MGG_11754* | DR | N | | heavy metal tolerance protein precursor [Pyrenophora tritici-repentis Pt-1C-BFP]. | | XP_001936908 | 2e-76 |
| 3.26 | MGG_05526 | DR | N | | ammonium transporter MEP2 [Neurospora crassa OR74A]. | | XP_961677 | 0.0 |
| 3.25 | MGG_13793* | DR | N | | nitrate transporter CrnA [Neosartorya fischeri NRRL 181]. | | XP_001264479 | 3e-143 |
| 3.17 | MGG_15331* | DR | N | | siderophore iron transporter [Coccidioides immitis RS]. | | XP_001247665 | 3e-51 |
| 3.11 | MGG_09015 | DR | N | | opsin-like protein [Gibberella fujikuroi]. | | CAD97459 | 2e-56 |
| 3.00 | MGG_05107* | DR | N | | general amino acid permease AGP3 [Neurospora crassa OR74A]. | | XP_957512 | 0.0 |
| 2.73 | MGG_12936* | DR | N | | alternative oxidase, mitochondrial precursor [Neurospora crassa OR74A]. | | XP_962086 | 2e-132 |
| 2.62 | MGG_00198* | DR | N | | flavohemoglobin [Fusarium oxysporum]. | | BAA33011 | 5e-123 |
| 2.58 | MGG_01516 | DR | N | | tetracycline-efflux transporter, putative [Talaromyces stipitatus ATCC 10500]. | | EED12910 | 1e-114 |
| 2.58 | MGG_04329* | DR | N | | MFS transporter, putative [Neosartorya fischeri NRRL 181]. | | XP_001266909 | 6e-72 |
| 2.58 | MGG_08056* | DR | N | | purine permease [Neurospora crassa OR74A]. | | XP_959265 | 0.0 |
| 2.44 | MGG_14937* | DR | N | | proline permease, putative [Aspergillus flavus NRRL3357]. | | EED47581 | 0.0 |
| 2.32 | MGG_07546 | DR | N | | MFS maltose permease [Aspergillus fumigatus Af293]. | | XP_747524 | 0.0 |
| 2.32 | MGG_10200* | DR | N | | small oligopeptide transporter, OPT family [Aspergillus clavatus NRRL 1]. | | XP_001275811 | 0.0 |
| 2.32 | MGG_15061* | DR | N | | PfkB family kinase, putative [Neosartorya fischeri NRRL 181]. | | XP_001265429 | 3e-49 |
| 2.32 | MGG_04385* | DR | N | | urea active transporter [Pyrenophora tritici-repentis Pt-1C-BFP]. | | XP_001934914 | 0.0 |
| 2.32 | MGG_10293* | DR | N | | sugar transporter, putative [Aspergillus flavus NRRL3357]. | | EED53374 | 7e-121 |
| 2.32 | MGG_12817* | DR | N | | MFS transporter, putative [Aspergillus clavatus NRRL 1]. | | XP_001275248 | 8e-158 |
| 2.32 | MGG_09941 | DR | N | | ABC transporter [Aspergillus fumigatus Af293]. | | XP_753691 | 0.0 |
| 2.32 | MGG_08369* | DR | N | | MFS transporter, putative [Talaromyces stipitatus ATCC 10500]. | | EED16840 | 6e-146 |
| 2.32 | MGG_14868* | DR | N | | MFS myo-inositol transporter [Aspergillus fumigatus Af293]. | | XP_752216 | 3e-140 |
| 2.04 | MGG_02101* | DR | N | | SAM independent family methyltransferase [Bifidobacterium thermophilum]. | | ACA23190 | 2e-27 |
| 2.00 | MGG_04934* | DR | N | | peptide transporter PTR2-A [Pyrenophora tritici-repentis Pt-1C-BFP]. | | XP_001939496 | 0.0 |
| 2.00 | MGG_03360 | DR | N | | related to carboxylic acid transport protein JEN1 [Neurospora crassa]. | | CAB88550 | 0.0 |
| 2.00 | MGG_07681* | DR | N | | C6 transcription factor [Aspergillus fumigatus Af293]. | | XP_001481491 | 6e-58 |
| 2.00 | MGG_09076* | DR | N | | MFS monocarboxylate transporter, putative [Neosartorya fischeri NRRL 181]. | | XP_001263879 | 7e-88 |
| 2.00 | MGG_05219 | DR | N | | UDP-Glc/Gal endoplasmic reticulum nucleotide sugar transporter  [Aspergillus clavatus NRRL 1]. | | XP_001269620 | 2e-115 |
| 2.00 | MGG_13279* | DR | N | | Na or K P-type ATPase [Neurospora crassa]. | | CAB65297 | 0.0 |
| 2.00 | MGG_02394* | DR | N | | C4-dicarboxylate transporter/malic acid transport protein, putative [Aspergillus flavus NRRL3357]. | | EED53359 | 1e-107 |
| 2.00 | MGG_08760 | DR | N | | MFS transporter [Aspergillus fumigatus Af293]. | | XP_749930 | 2e-114 |
| 1.87 | MGG_05979 | DR | N | | major facilitator transporter [Maricaulis maris MCS10]. | | YP_758251 | 2e-05 |
| 1.86 | MGG_13669 | DR | N | | MFS peptide transporter, putative [Aspergillus flavus NRRL3357]. | | EED49889 | 5e-170 |
| 1.82 | MGG_08580* | DR | N | | Na/H antiporter, putative [Aspergillus flavus NRRL3357]. | | EED54964 | 2e-158 |
| 1.82 | MGG_04927* | DR | N | | transporter-like protein [Magnaporthe grisea]. | | AAX07640 | 0.0 |
| 1.81 | MGG_02743 | DR | N | | WD repeat protein [Aspergillus clavatus NRRL 1]. | | XP_001270710 | 2e-149 |
| 1.81 | MGG_06476* | DR | N | | VPS9 domain protein [Aspergillus fumigatus Af293]. | | XP_755422 | 1e-123 |
| 1.81 | MGG_14906* | DR | N | | WD domain containing protein [Pyrenophora tritici-repentis Pt-1C-BFP]. | | XP_001941588 | 2e-40 |
| 1.81 | MGG_04640* | DR | N | | sulfate transporter, putative [Aspergillus clavatus NRRL 1]. | | XP_001269658 | 0.0 |
| 1.81 | MGG_09906* | DR | N | | mitochondrial phosphate carrier protein 2 [Neurospora crassa OR74A]. | | XP_963916 | 2e-144 |
| 1.73 | MGG_06751* | DR | N | | Pc22g23390 [Penicillium chrysogenum Wisconsin 54-1255]. | | CAP99627 | 0.037 |
| 1.67 | MGG_04855* | DR | N | | ATP-dependent bile acid permease [Neosartorya fischeri NRRL 181]. | | XP_001259771 | 0.0 |
| 1.66 | MGG_09281 | DR | N | | cytochrome c oxidase copper chaperone protein [Botryotinia fuckeliana B05.10]. | | XP_001547644 | 8e-16 |
| 2.81 | MGG_13338* | UR | N | | MFS transporter, putative [Neosartorya fischeri NRRL 181]. | | XP_001258781 | 0.0 |
| 2.81 | MGG_15203* | UR | N | | related to chloride-bicarbonate anion exchanger AE2 [Neurospora crassa]. | | CAE76152 | 0.0 |
| 2.75 | MGG_08426* | UR | N | | amino acid transporter [Pyrenophora tritici-repentis Pt-1C-BFP]. | | XP_00193879 | 1e-113 |
| 2.72 | MGG_03299 | UR | N | | phosphate permease [Gibberella zeae]. | | BAA33769 | 0.0 |
| 2.68 | MGG_09354 | UR | N | | oligopeptide transporter, putative [Talaromyces stipitatus ATCC 10500]. | | EED14685 | 0.0 |
| 2.58 | MGG_05643* | UR | N | | calcium channel subunit Cch1 [Neosartorya fischeri NRRL 181]. | | XP_001264658 | 0.0 |
| 2.42 | MGG_00085* | UR | N | | lactose permease [Pyrenophora tritici-repentis Pt-1C-BFP]. | | XP_001933299 | 0.0 |
| 2.39 | MGG_10916* | UR | Y | | putative sugar transporter [Gibberella moniliformis]. | | ABV60279 | 0.0 |
| 2.32 | MGG_12861 | UR | N | | high affinity methionine permease [Penicillium marneffei ATCC 18224]. | | XP_002152766 | 1e-80 |
| 2.00 | MGG_11429* | UR | N | | oligonucleotide transporter [Pyrenophora tritici-repentis Pt-1C-BFP]. | | XP_001932829 | 5e-166 |
| 2.00 | MGG_15313* | UR | N | | ankyrin repeat protein [Neosartorya fischeri NRRL 181]. | | XP_001264307 | 0.0 |
| 2.00 | MGG_07297* | UR | N | | autophagy ubiquitin-activating enzyme ApgG, putative [Penicillium marneffei ATCC 18224]. | | XP_002144542 | 0.0 |
| 2.00 | MGG_05379 | UR | N | | guanine nucleotide exchange factor Vps9, putative [Aspergillus flavus NRRL3357]. | | EED46970 | 0.0 |
| 2.00 | MGG_02941* | UR | N | | hepatocyte growth factor-regulated tyrosine kinase substrate-like protein [Oryza sativa (japonica cultivar-group)]. | | AAL58181 | 0.005 |
| 2.00 | MGG_11726* | UR | N | | golgi transport complex subunit Cog4 [Pyrenophora tritici-repentis Pt-1C-BFP]. | | XP_001932464 | 0.0 |
| 2.00 | MGG_05095* | UR | N | | non-repetitive nucleoporin, putative [Penicillium marneffei ATCC 18224]. | | XP_002152109 | 0.0 |
| 2.00 | MGG_03701* | UR | Y | | MFS transporter, putative [Aspergillus clavatus NRRL 1]. | | XP_001269781 | 1e-128 |
| 2.00 | MGG_06435 | UR | N | | TRAPP complex subunit (Bet5), putative [Neosartorya fischeri NRRL 181]. | | XP_001266039 | 5e-47 |
| 2.00 | MGG_01054* | UR | N | | AvaB protein [Emericella nidulans]. | | BAC57965 | 0.0 |
| 2.00 | MGG_00946* | UR | N | | Poly(A)+ RNA export protein [Neurospora crassa OR74A]. | | XP_961078 | 1e-169 |
| 2.00 | MGG_10027* | UR | N | | putative K, P-type ATPase [Magnaporthe grisea]. | | CAF25028 | 0.0 |
| 1.89 | MGG_06735* | UR | N | | Golgi membrane protein (Coy1), putative [Penicillium marneffei ATCC 18224]. | | XP_002146005 | 0.0 |
| 1.85 | MGG_06097* | UR | Y | | mitochondrial import receptor subunit (Tom20) [Aspergillus fumigatus Af293]. | | XP_751021 | 4e-37 |
| 1.81 | MGG_03548 | UR | N | | spindle-pole body protein (Pcp1), putative [Talaromyces stipitatus ATCC 10500]. | | EED20306 | 8e-96 |
| 1.70 | MGG_13028* | UR | N | | mitochondrial import inner membrane translocase subunit tim-54 [Neurospora crassa OR74A]. | | XP_962149 | 2e-109 |
| 1.62 | MGG_01274* | UR | N | | RNP domain protein [Aspergillus flavus NRRL3357]. | | EED56410 | 6e-53 |
| 1.58 | MGG_01425* | UR | N | | MFS transporter, putative [Neosartorya fischeri NRRL 181]. | | XP_001259585 | 3e-114 |
| 1.58 | MGG_03118 | UR | N | | MFS transporter, putative [Neosartorya fischeri NRRL 181]. | | XP_001264041 | 0.0 |
| 1.58 | MGG_04372 | UR | Y | | MFS sugar transporter, putative [Aspergillus flavus NRRL3357]. | | EED57403 | 2e-96 |
| 1.58 | MGG_01493* | UR | N | | mitochondrial carrier protein LEU5 [Neurospora crassa OR74A]. | | XP_962539 | 3e-138 |
| 1.58 | MGG_06141* | UR | N | | transporter of the mitochondrial inner membrane, putative [Candida dubliniensis CD36]. | | CAX41974 | 7e-72 |
| 1.58 | MGG_10289 | UR | N | | MFS transporter, putative [Aspergillus clavatus NRRL 1]. | | XP_001270167 | 2e-131 |
| 1.58 | MGG_06427* | UR | N | | mitochondrial intermembrane space translocase subunit Tim9, putative [Talaromyces stipitatus ATCC 10500]. | | EED19768 | 3e-18 |
| 1.58 | MGG_13334* | UR | N | | general amino acid permease (Agp2), putative [Neosartorya fischeri NRRL 181]. | | XP_001267394 | 7e-137 |
| 1.53 | MGG_15388* | UR | N | | sugar transporter, putative [Aspergillus flavus NRRL3357]. | | EED51606 | 8e-132 |
|  |  |  |  | |  | |  |  |
| Pathogenicity (GO:0009045) | | | | | | | | |
| 9.72 | MGG_10315* | DR | Y | | hydrophobin [Beauveria bassiana]. | | ABP58683 | 5e-08 |
| 2.00 | MGG_04621* | DR | N | | YT521-B-like splicing factor, putative [Penicillium marneffei ATCC 18224]. | | XP_002146401 | 7e-80 |
| 1.73 | MGG_07015* | DR |  | | DNA repair protein Rad7, protein [Neosartorya fischeri NRRL 181]. | | XP_001261304 | 3e-102 |
| 2.00 | MGG_11899* | UR | N | | SH3 domain protein [Neosartorya fischeri NRRL 181]. | | XP_001263433 | 0.0 |
| 1.73 | MGG_00760* | UR | N | | serine/threonine protein kinase [Epichloe festucae]. | | ACB30135 | 0.0 |
| 1.58 | MGG_10510* | UR | Y | | ribonuclease T2 precursor [Pyrenophora tritici-repentis Pt-1C-BFP]. | | XP_001938454 | 6e-83 |
| 1.58 | MGG_09471* | UR | N | | neutral trehalase [Neurospora crassa OR74A]. | | XP_960845 | 0.0 |
| GO:0016740 : transferase activity | | | | | | | | |
| 5.36 | MGG_00678* | DR | N | | Pc12g02220 [Penicillium chrysogenum Wisconsin 54-1255]. | | CAP79849 | 2e-45 |
| 4.89 | MGG_10108* | DR | N | | glutathione S-transferase, putative [Aspergillus clavatus NRRL 1]. | | XP_001273707 | 3e-73 |
| 8.33 | MGG_09138* | DR | N | | glutathione S-transferase Ure2-like [Aspergillus fumigatus Af293]. | | XP_751380 | 2e-69 |
| 4.07 | MGG_02105* | DR | N | | Pc13g04680 [Penicillium chrysogenum Wisconsin 54-1255]. | | CAP91537 | 4e-98 |
| 3.70 | MGG_13819* | DR | N | | MarR family transcriptional regulator [Serratia proteamaculans 568] | | YP_001478700 | 2e-49 |
| 3.49 | MGG_08189* | DR | N | | aromatic amino acid aminotransferase 1 [Pyrenophora tritici-repentis Pt-1C-BFP]. | | XP_001941234 | 2e-168 |
| 2.32 | MGG_05017* | DR | N | | glutamyl-tRNA(Gln) amidotransferase, subunit A [Penicillium marneffei ATCC 18224]. | | XP_002145146 | 8e-60 |
| 2.22 | MGG_07627* | DR | N | | homoserine acetyltransferase family protein [Pyrenophora tritici-repentis Pt-1C-BFP]. | | XP_001940705 | 3e-141 |
| 2.00 | MGG_01349* | DR | N | | Pc20g00330 [Penicillium chrysogenum Wisconsin 54-1255]. | | CAP85362 | 1e-46 |
| 2.00 | MGG_05330* | DR | N | | acetyltransferase [Pyrenophora tritici-repentis Pt-1C-BFP]. | | XP_001940025 | 5e-44 |
| 2.00 | MGG_04254 | DR | N | | Pc13g02250 [Penicillium chrysogenum Wisconsin 54-1255]. | | CAP91294 | 2e-08 |
| 1.80 | MGG_02120* | DR | N | | O-methyltransferase, putative [Penicillium marneffei ATCC 18224]. | | XP_002152906 | 3e-76 |
| 1.62 | MGG_08289 | DR | N | | acetylase [Ajellomyces capsulatus]. | | ACC64455 | 5e-102 |
| 1.58 | MGG_14001* | DR | N | | tRNA (uracil-5-)-methyltransferase TRM9 [Schizosaccharomyces japonicus yFS275]. | | XP_002173263 | 3e-57 |
| 1.58 | MGG_08768* | DR | N | | arsenite methyltransferase, putative [Talaromyces stipitatus ATCC 10500]. | | EED14234 | 1e-58 |
| 1.58 | MGG_00388* | DR | Y | | NACHT domain protein [Talaromyces stipitatus ATCC 10500]. | | EED12088 | 8e-67 |
| 1.58 | MGG_01191 | DR | N | | polysaccharide synthase Cps1, putative [Talaromyces stipitatus ATCC 10500]. | | EED12592 | 2e-116 |
| 1.58 | MGG_13924* | DR | N | | serine palmitoyltransferase 2 [Pyrenophora tritici-repentis Pt-1C-BFP]. | | XP_001935084 | 6e-106 |
| 2.81 | MGG_04036 | UR | N | | Methyltransferase type 11 [Rhizobium leguminosarum bv. trifolii WSM1325]. | | ZP_02298283 | 6e-48 |
| 2.81 | MGG_00073 | UR | N | | topoisomerase family protein TRF4, putative [Talaromyces stipitatus ATCC 10500]. | | EED21665 | 4e-98 |
| 2.32 | MGG_06441* | UR | N | | Pc22g12090 [Penicillium chrysogenum Wisconsin 54-1255]. | | CAP98497 | 1e-30 |
| 2.32 | MGG_02993 | UR | N | | GNAT family acetyltransferase, putative [Neosartorya fischeri NRRL 181]. | | XP_001257400 | 1e-08 |
| 2.00 | MGG_06498* | UR | N | | 5'-methylthioadenosine phosphorylase (Meu1), putative [Neosartorya fischeri NRRL 181]. | | XP_001257988 | 5e-115 |
| 2.00 | MGG_14765* | UR | N | | UbiE/COQ5 family methyltransferase, putative [Aspergillus clavatus NRRL 1]. | | XP_001275403 | 1e-17 |
| 1.96 | MGG_15353 | UR | Y | | hyaluronan synthase, putative [Penicillium marneffei ATCC 18224]. | | XP_002149862 | 9e-124 |
| 1.86 | MGG_15354* | UR | N | | glycosyl transferase [Aspergillus fumigatus Af293]. | | XP_747141 | 3e-40 |
| 1.62 | MGG_05254* | UR | N | | histone H3 methyltransferase, putative [Aspergillus flavus NRRL3357]. | | EED49233 | 1e-91 |
| 1.58 | MGG_02254 | UR | N | | methyltransferase, putative [Neosartorya fischeri NRRL 181]. | | XP_001260896 | 1e-49 |
| 1.58 | MGG_00810* | UR | N | | DHHC zinc finger membrane protein [Talaromyces stipitatus ATCC10500]. | | EED23084 | 7e-71 |
| 1.58 | MGG_03561 | UR | N | | L-A virus GAG protein N-acetyltransferase [Neurospora crassa OR74A]. | | XM_954506 | 1e-79 |
| 1.58 | MGG_06930 | UR | N | | Snf1 kinase complex beta-subunit Gal83 [Aspergillus fumigatus Af293]. | | XP_747628 | 4e-60 |
|  |  |  |  | |  | |  |  |
| **Process Unknown** | | | | | | | | |
| 2.61 | MGG_05766 | DR | N | | Pc21g09230 [Penicillium chrysogenum Wisconsin 54-1255]. | | CAP95820 | 2e-07 |
| 1.58 | MGG_02499* | DR | N | | Pc16g02200 [Penicillium chrysogenum Wisconsin 54-1255]. | | CAP92890 | 2e-58 |
| 1.58 | MGG_03714* | DR | N | | annexin ANXC4 [Aspergillus clavatus NRRL 1]. | | XP_001270721 | 7e-102 |
| 1.58 | MGG_06021* | DR | N | | TBC domain protein, putative [Neosartorya fischeri NRRL 181]. | | XP_001265259 | 3e-98 |
| 1.58 | MGG_09295* | DR | N | | translation initiation protein Sua5 [Neosartorya fischeri NRRL 181]. | | XP_001260414 | 1e-106 |
| 1.58 | MGG_04076* | DR | N | | low-density lipoprotein receptor YWTD repeat [Burkholderia sp. H160]. | | ZP_03266143 | 4e-57 |
| 1.58 | MGG_00693* | DR | N | | Pc21g11240 [Penicillium chrysogenum Wisconsin 54-1255]. | | CAP96021 | 3e-80 |
| 1.58 | MGG_00322* | DR | Y | | Pc16g02730 [Penicillium chrysogenum Wisconsin 54-1255]. | | CAP92943 | 7e-102 |
| 1.58 | MGG_03368* | DR | Y | | Pc20g13990 [Penicillium chrysogenum Wisconsin 54-1255]. | | CAP86728 | 4e-15 |
| 1.58 | MGG_09165 | DR | N | | putative sialidase [Trichophyton rubrum]. | | ABG67894 | 2e-09 |
| 1.58 | MGG_12213* | DR | N | | HET and Ankyrin domain protein [Aspergillus flavus NRRL3357]. | | EED51601 | 7e-52 |
| 1.58 | MGG_06271 | DR | N | | Pc20g07730 [Penicillium chrysogenum Wisconsin 54-1255]. | | CAP86102 | 3e-20 |
| 1.58 | MGG_07417* | DR | N | | DUF895 domain membrane protein [Aspergillus flavus NRRL3357]. | | EED51620 | 4e-159 |
| 1.58 | MGG_09811* | DR | N | | XAP5 domain protein [Talaromyces stipitatus ATCC 10500]. | | EED13160 | 4e-71 |
| 1.58 | MGG_00250* | DR | N | | arrestin (or S-antigen) domain protein [Talaromyces stipitatus ATCC 10500]. | | EED12649 | 4e-52 |
| 1.58 | MGG_05436* | DR | N | | PUA RNA binding domain protein, putative [Talaromyces stipitatus ATCC 10500] | | EED24032 | 3e-50 |
| 1.58 | MGG_03892* | DR | N | | Pc21g20110 [Penicillium chrysogenum Wisconsin 54-1255]. | | CAP96908 | 9e-89 |
| 1.58 | MGG_11305* | DR | N | | YqcI [Bacillus amyloliquefaciens FZB42]. | | YP_001419963 | 6e-32 |
| 1.58 | MGG_03161* | DR | N | | Pc22g11110 [Penicillium chrysogenum Wisconsin 54-1255]. | | CAP98399 | 6e-33 |
| 1.58 | MGG_08653 | DR | N | | Pc16g09120 [Penicillium chrysogenum Wisconsin 54-1255]. | | CAP93582 | 5e-12 |
| 1.58 | MGG_09547* | DR | N | | actin-2 [Neurospora crassa OR74A]. | | XP_961238 | 0.0 |
| 1.58 | MGG_06171 | DR | N | | integral membrane protein Pth11-like, putative [Aspergillus flavus NRRL3357]. | | EED53109 | 2e-24 |
| 1.81 | MGG_13219* | DR | N | | DUF323 domain protein [Aspergillus clavatus NRRL 1]. | | XP_001275843 | 0.0 |
| 1.76 | MGG_03249* | DR | N | | Pc12g11640 [Penicillium chrysogenum Wisconsin 54-1255]. | | CAP80791 | 5e-07 |
| 1.74 | MGG_01890 | DR | Y | | integral membrane protein [Penicillium marneffei ATCC 18224]. | | XP_002149317 | 1e-20 |
| 1.74 | MGG_05849 | DR | N | | Pc15g00300 [Penicillium chrysogenum Wisconsin 54-1255]. | | CAP82916 | 6e-23 |
| 1.70 | MGG_04085* | DR | N | | AAA family ATPase, putative [Neosartorya fischeri NRRL 181]. | | XP_001261618 | 3e-110 |
| 1.66 | MGG_12316* | DR | N | | esdC homologue [Sordaria macrospora]. | | CAH03680 | 6e-84 |
| 1.58 | MGG_00208* | DR | N | | Pc22g03270 [Penicillium chrysogenum Wisconsin 54-1255]. | | CAP97615 | 3e-51 |
| 1.58 | MGG_08741 | DR | N | | mRNA binding post-transcriptional regulator (Csx1) [Aspergillus fumigatus Af293]. | | XP_746709 | 2e-140 |
| 2.32 | MGG_04757* | DR | Y | | unnamed protein product [Podospora anserina]. | | CAP65629 | 7e-79 |
| 2.32 | MGG_08489 | DR | N | | Pc20g06430 [Penicillium chrysogenum Wisconsin 54-1255]. | | CAP85972 | 6e-05 |
| 2.32 | MGG_04541* | DR | Y | | putative lipoprotein [Myxococcus xanthus DK 1622]. | | YP_633198 | 2e-14 |
| 2.32 | MGG_00796 | DR |  | | predicted protein [Aspergillus terreus NIH2624]. | | XP_001210259 | 6e-44 |
| 2.32 | MGG_03826* | DR | Y | | kelch repeat-containing protein [Methylobacterium extorquens PA1]. | | YP_001638060 | 4e-24 |
| 2.32 | MGG_14010* | DR | N | | Pc22g16800 [Penicillium chrysogenum Wisconsin 54-1255]. | | CAP98968 | 9e-08 |
| 2.32 | MGG_00338* | DR | N | | SNF2 family helicase, putative [Aspergillus clavatus NRRL 1]. | | XP_001271954 | 4e-141 |
| 2.22 | MGG_09061* | DR | N | | Pc22g17450 [Penicillium chrysogenum Wisconsin 54-1255]. | | CAP99033 | 9e-23 |
| 2.17 | MGG_01945* | DR | N | | Pc21g08800 [Penicillium chrysogenum Wisconsin 54-1255]. | | CAP95777 | 0.012 |
| 2.17 | MGG_01547* | DR | N | | methyltransferase LaeA-like, putative [Talaromyces stipitatus ATCC10500]. | | EED237 | 9e-34 |
| 2.17 | MGG_05994* | DR | N | | hypothetical protein FG08848.1 [Gibberella zeae PH-1]. | | XP_389024 76 | 8e-46 |
| 2.06 | MGG_00443 | DR | N | | unnamed protein product [Podospora anserina]. | | XP_001905766 | 1e-09 |
| 2.05 | MGG_09883* | DR | N | | Pc21g04050 [Penicillium chrysogenum Wisconsin 54-1255]. | | CAP95302 | 3e-13 |
| 2.00 | MGG_09868* | DR | N | | Pc12g06740 [Penicillium chrysogenum Wisconsin 54-1255]. | | CAP80301 | 1e-10 |
| 2.00 | MGG_14857* | DR | N | | Pc22g07220 [Penicillium chrysogenum Wisconsin 54-1255]. | | CAP98010 | 4e-06 |
| 2.00 | MGG_11567* | DR | N | | Pc13g11960 [Penicillium chrysogenum Wisconsin 54-1255]. | | CAP92265 | 2e-38 |
| 2.00 | MGG_02752 | DR | N | | glycine dehydrogenase subunit 2 [Chloroflexus aurantiacus J-10-fl]. | | YP_001637074 | 0.21 |
| 2.00 | MGG_02004 | DR | N | | PREDICTED: similar to myosin VC [Bos taurus]. | | XP_611694 | 0.18 |
| 2.00 | MGG_01548* | DR | N | | Pc21g02240 [Penicillium chrysogenum Wisconsin 54-1255]. | | CAP95121 | 5e-46 |
| 2.00 | MGG_11188* | DR | N | | Pc21g21920 [Penicillium chrysogenum Wisconsin 54-1255]. | | CAP97089 | 0.003 |
| 2.00 | MGG_14659* | DR | N | | Pc22g16070 [Penicillium chrysogenum Wisconsin 54-1255]. | | CAP98895 | 4e-56 |
| 2.00 | MGG_02775 | DR | N | | C2H2 finger domain protein, putative [Aspergillus clavatus NRRL 1]. | | XP_001270347 | 2e-23 |
| 2.00 | MGG_03141* | DR | N | | unnamed protein product [Podospora anserina]. | | XP_001909760 | 3e-15 |
| 2.00 | MGG_03234* | DR | N | | Pc22g18580 [Penicillium chrysogenum Wisconsin 54-1255]. | | CAP99146 | 4e-27 |
| 2.00 | MGG_05411* | DR | N | | unnamed protein product [Podospora anserina]. | | XP_001905197 | 2e-15 |
| 2.00 | MGG_09240 | DR | N | | F-box domain protein [Neosartorya fischeri NRRL 181]. | | XP_001257861 | 5e-45 |
| 1.94 | MGG_05806 | DR | N | | ankyrin repeat-containing protein, putative [Penicillium marneffei ATCC 18224]. | | XP_002149104 | 6e-04 |
| 1.91 | MGG_03462* | DR | N | | general amidase GmdA, putative [Aspergillus fumigatus A1163]. | | EDP49372 | 5e-123 |
| 1.87 | MGG_02976 | DR | N | | kinesin light chain 3 [Pyrenophora tritici-repentis Pt-1C-BFP]. | | XP_001941711 | 0.0 |
| 1.87 | MGG_03519 | DR | N | | ipa protein [Leptosphaeria maculans]. | | ABY83629 | 1e-142 |
| 1.58 | MGG_00696* | DR | N | | Pc22g23630 [Penicillium chrysogenum Wisconsin 54-1255]. | | CAP99651 | 2e-35 |
| 6.18 | MGG_14863* | DR | N | | trans-aconitate 2-methyltransferase [Pyrenophora tritici-repentis Pt-1C-BFP]. | | XP_001938347 | 3e-66 |
| 5.39 | MGG_05940 | DR | N | | short chain dehydrogenase/reductase, putative [Neosartorya fischeri NRRL 181]. | | XP_001262718 | 2e-45 |
| 5.39 | MGG_12981* | DR | N | | Cupin domain protein [Aspergillus flavus NRRL3357]. | | EED49549 | 5e-29 |
| 5.25 | MGG_11335* | DR | Y | | related to glyoxal oxidase precursor [Neurospora crassa]. | | CAE76318 | 5e-23 |
| 5.05 | MGG_01461* | DR | N | | Pc13g06350 [Penicillium chrysogenum Wisconsin 54-1255]. | | CAP91704 | 1e-52 |
| 4.95 | MGG_04164* | DR | N | | DUF427 domain protein [Aspergillus clavatus NRRL 1]. | | XP_001273683 | 3e-32 |
| 4.85 | MGG_08519 | DR | N | | aldehyde reductase (GliO), putative [Neosartorya fischeri NRRL 181]. | | XP_001266331 | 4e-83 |
| 4.61 | MGG_02988* | DR | N | | RTA1 domain protein, putative [Neosartorya fischeri NRRL 181]. | | XP_001258655 | 6e-36 |
| 4.41 | MGG_14882* | DR | N | | epoxide hydrolase [Aspergillus niger]. | | XP_001397530 | 2e-99 |
| 4.17 | MGG_01297 | DR | N | | Pc12g08070 [Penicillium chrysogenum Wisconsin 54-1255]. | | CAP80434 | 8e-63 |
| 4.17 | MGG_01764 | DR | Y | | integral membrane protein [Talaromyces stipitatus ATCC 10500]. | | EED17189 | 3e-60 |
| 4.09 | MGG_02647* | DR | Y | | UVI-1 [Bipolaris oryzae]. | | BAA96293 | 2e-103 |
| 4.09 | MGG_11663* | DR | N | | zinc-binding oxidoreductase CipB [Pyrenophora tritici-repentis Pt-1C-BFP]. | | XP_001930726 | 5e-87 |
| 4.00 | MGG_04018 | DR | N | | BCS1-like ATPase, putative [Penicillium marneffei ATCC 18224]. | | XP_002149665 | 2e-87 |
| 3.80 | MGG_00070 | DR | N | | molybdenum cofactor biosynthesis protein A [Cyanothece sp. PCC 8801]. | | YP_002371971 | 0.57 |
| 3.70 | MGG_04369 | DR | N | | AAA ATPase, putative [Aspergillus flavus NRRL3357]. | | EED51143 | 1e-112 |
| 3.66 | MGG_01410* | DR | N | | Pc21g02430 [Penicillium chrysogenum Wisconsin 54-1255]. | | CAP95140 | 2e-136 |
| 3.58 | MGG_13126* | DR | N | | Pc13g14230 [Penicillium chrysogenum Wisconsin 54-1255]. | | CAP92492 | 2e-17 |
| 3.49 | MGG_07791 | DR | Y | | Pc13g06930 [Penicillium chrysogenum Wisconsin 54-1255]. | | CAP91762 | 7e-21 |
| 3.46 | MGG_03501* | DR | N | | DUF1264 domain protein [Penicillium marneffei ATCC 18224]. | | XP_002145074 | 3e-71 |
| 3.17 | MGG_15043* | DR | N | | related to heterokaryon incompatibility protein het-6 [Neurospora crassa]. | | CAD37033 | 2e-32 |
| 3.09 | MGG_08936* | DR | N | | unnamed protein product [Podospora anserina]. | | XP_001903556 | 2e-54 |
| 3.00 | MGG_00121* | DR | N | | helix-turn-helix domain-containing protein [Burkholderia phymatum STM815]. | | YP_001861187 | 6e-24 |
| 2.91 | MGG_02854* | DR | Y | | pirin domain protein, putative [Penicillium marneffei ATCC 18224]. | | XP_002146824 | 1e-92 |
| 2.86 | MGG_08304* | DR | N | | Mechanosensitive ion channel family [Talaromyces stipitatus ATCC10500]. | | EED22784 | 8e-171 |
| 2.85 | MGG_03333* | DR | N | | integral membrane protein [Aspergillus fumigatus A1163]. | | EDP47183 | 0.007 |
| 2.81 | MGG_03367* | DR | Y | | Pc20g13980 [Penicillium chrysogenum Wisconsin 54-1255]. | | CAP86727 | 5e-41 |
| 2.81 | MGG_08873* | DR | N | | Pc12g07620 [Penicillium chrysogenum Wisconsin 54-1255]. | | CAP80389 | 9e-118 |
| 2.77 | MGG_00756 | DR | Y | | Pc21g10110 [Penicillium chrysogenum Wisconsin 54-1255]. | | CAP95908 | 0.005 |
| 2.65 | MGG_10517 | DR | N | | BTB/POZ domain protein [Aspergillus fumigatus A1163]. | | EDP53444 | 1e-09 |
| 2.58 | MGG_14184* | DR | N | | Pc21g03780 [Penicillium chrysogenum Wisconsin 54-1255]. | | CAP95275 | 2e-07 |
| 2.58 | MGG_10480 | DR | N | | ankyrin repeat protein [Aspergillus fumigatus Af293]. | | XP_752820 | 4e-21 |
| 2.58 | MGG_07625* | DR | Y | | NH(3)-dependent NAD(+) synthetase [Erwinia tasmaniensis Et1/99]. | | YP_001907784 | 0.95 |
| 2.58 | MGG_06473 | DR | N | | C6 zinc finger domain protein [Neosartorya fischeri NRRL 181]. | | XP_001261821 | 4e-08 |
| 2.58 | MGG_02916 | DR | Y | | unnamed protein product [Podospora anserina]. | | XP_001903068 | 4e-07 |
| 2.51 | MGG_09477* | DR | N | | Pc15g00980 [Penicillium chrysogenum Wisconsin 54-1255]. | | CAP82984 | 9e-04 |
| 2.50 | MGG_00082* | DR | N | | related to heterokaryon incompatibility protein het-6 [Neurospora crassa]. | | CAD37033 | 3e-28 |
| 2.49 | MGG_10699* | DR | Y | | Pc22g07240 [Penicillium chrysogenum Wisconsin 54-1255]. | | CAP98012 | 3e-16 |
| 2.46 | MGG_01243* | DR | Y | | unnamed protein product [Podospora anserina]. | | XP_001905237 | 1e-19 |
| 2.36 | MGG_06902* | DR | N | | kinesin [Trypanosoma brucei TREU927]. | | XP_844423 | 0.076 |
| 4.80 | MGG_07356* | UR | Y | | isoamyl alcohol oxidase [Aspergillus fumigatus Af293]. | | XP_746836 | 9e-30 |
| 3.32 | MGG_00245* | UR | Y | | Pc16g13040 [Penicillium chrysogenum Wisconsin 54-1255]. | | CAP93974 | 6e-36 |
| 3.32 | MGG_08574* | UR | N | | Pc22g16970 [Penicillium chrysogenum Wisconsin 54-1255]. | | CAP98985 | 1e-13 |
| 3.00 | MGG_08367* | UR | N | | Pc21g01170 [Penicillium chrysogenum Wisconsin 54-1255]. | | CAP95014 | 2e-23 |
| 2.81 | MGG_08536 | UR | N | | related to nitrate assimilation regulatory protein nirA [Neurospora crassa]. | | CAD79658 | 4e-25 |
| 2.81 | MGG_01057* | UR | N | | PtaB protein, putative [Neosartorya fischeri NRRL 181]. | | XP_001260750 | 7e-44 |
| 2.73 | MGG_04889 | UR | Y | | Pc12g08970 [Penicillium chrysogenum Wisconsin 54-1255]. | | CAP80524 | 5e-10 |
| 2.70 | MGG_03630* | UR | N | | fr [Neurospora crassa]. | | BAA36220 | 0.0 |
| 2.58 | MGG_08935 | UR | N | | Pc22g04760 [Penicillium chrysogenum Wisconsin 54-1255]. | | CAP97764 | 1e-21 |
| 2.58 | MGG_10614* | UR | N | | isoflavone reductase family protein [Neosartorya fischeri NRRL 181]. | | XP_001259744 | 9e-41 |
| 2.58 | MGG_07765* | UR | N | | Pc16g10920 [Penicillium chrysogenum Wisconsin 54-1255]. | | CAP93762 | 1e-11 |
| 2.51 | MGG_13622* | UR | Y | | endoglucanase [Aspergillus fumigatus Af293]. | | XP_750843 | 3e-45 |
| 2.50 | MGG_00469* | UR | Y | | Sad1/UNC domain protein [Talaromyces stipitatus ATCC 10500]. | | EED18185 | 2e-108 |
| 2.32 | MGG_09732* | UR | Y | | feruloyl esterase B precursor, putative [Aspergillus flavus  NRRL3357]. | | EED45098 | 1e-106 |
| 2.32 | MGG_01751 | UR | N | | F-box and WD domain protein [Talaromyces stipitatus ATCC 10500]. | | EED16921 | 2e-180 |
| 2.32 | MGG_00992* | UR | Y | | N-glycosylation site corresponding to basepairs 208-210 [Blumeria graminis]. | | AAB05211 | 3e-17 |
| 2.32 | MGG_06854* | UR | N | | integral membrane protein (Pth11), putative [Aspergillus flavus NRRL3357]. | | EED45803 | 9e-12 |
| 2.32 | MGG_12321* | UR | N | | Pc15g01940 [Penicillium chrysogenum Wisconsin 54-1255]. | | CAP83080 | 2e-08 |
| 2.32 | MGG_04078* | UR | Y | | Pc21g18650 [Penicillium chrysogenum Wisconsin 54-1255]. | | CAP96762 | 1e-112 |
| 2.32 | MGG_14969* | UR | N | | rbm25 protein, putative [Aspergillus flavus NRRL3357]. | | EED52776 | 4e-126 |
| 2.32 | MGG_03910 | UR | Y | | Pc21g20190 [Penicillium chrysogenum Wisconsin 54-1255]. | | CAP96916 | 8e-24 |
| 2.32 | MGG_03227* | UR | N | | mitochondrial ATP synthase epsilon chain domain-containing protein [Botryotinia fuckeliana B05.10]. | | XP_001558839 | 3e-25 |
| 2.32 | MGG_06218* | UR | N | | R3H domain protein, putative [Penicillium marneffei ATCC 18224]. | | XP_002151100 | 2e-78 |
| 2.32 | MGG_08057* | UR | Y | | Pc13g08240 [Penicillium chrysogenum Wisconsin 54-1255]. | | CAP91893 | 1e-104 |
| 2.32 | MGG_02882* | UR | N | | Pc20g05000 [Penicillium chrysogenum Wisconsin 54-1255]. | | CAP85829 | 4e-04 |
| 2.32 | MGG_04040* | UR | N | | DEAH-box RNA helicase (Dhr1), putative [Aspergillus fumigatus A1163]. | | EDP47552 | 0.0 |
| 2.15 | MGG_04873* | UR | N | | CipC-like antibiotic response protein, putative [Talaromyces stipitatus ATCC 10500]. | | EED18081 | 3e-26 |
| 2.00 | MGG_12967* | UR | N | | Pc16g06240 [Penicillium chrysogenum Wisconsin 54-1255]. | | CAP93294 | 3e-09 |
| 2.00 | MGG_03886* | UR | Y | | nuclear envelope protein, putative [Aspergillus flavus NRRL3357]. | | EED53898 | 2e-98 |
| 2.00 | MGG_05048* | UR | N | | Pc21g20000 [Penicillium chrysogenum Wisconsin 54-1255]. | | XP_366911 | 1e-58 |
| 2.00 | MGG_12758* | UR | N | | deacetylase complex subunit Sds3, putative [Aspergillus flavus NRRL3357]. | | EED54816 | 6e-46 |
| 2.00 | MGG_06535* | UR | N | | Pc18g06020 [Penicillium chrysogenum Wisconsin 54-1255]. | | CAP94826 | 2e-32 |
| 2.00 | MGG_08632 | UR | N | | ubiquitin interaction motif protein [Aspergillus clavatus NRRL 1]. | | XP_001274207 | 4e-26 |
| 2.00 | MGG_06934 | UR | N | | Pc20g11870 [Penicillium chrysogenum Wisconsin 54-1255]. | | CAP86516 | 3e-31 |
| 2.00 | MGG_01982 | UR | N | | capsular associated protein [Aspergillus fumigatus Af293]. | | XP_750279 | 1e-113 |
| 2.00 | MGG_01628 | UR | N | | cyclase [Hypocrea virens]. | | ABE60722 | 2e-23 |
| 2.00 | MGG_01456* | UR | N | | Pc16g13610 [Penicillium chrysogenum Wisconsin 54-1255]. | | CAP94031 | 1e-125 |
| 2.00 | MGG_01287* | UR | N | | Pc22g12410 [Penicillium chrysogenum Wisconsin 54-1255]. | | CAP98529 | 2e-117 |
| 2.00 | MGG_03456* | UR | Y | | conidiation-specific protein, putative [Penicillium marneffei ATCC 18224]. | | XP_002146063 | 7e-20 |
| 2.00 | MGG_09506 | UR | Y | | Pc12g07290 [Penicillium chrysogenum Wisconsin 54-1255]. | | CAP80356 | 3e-103 |
| 1.90 | MGG_08594* | UR | N | | Pc18g02910 [Penicillium chrysogenum Wisconsin 54-1255]. | | CAP94515 | 9e-54 |
| 1.87 | MGG_00125* | UR | N | | Pc18g01260 [Penicillium chrysogenum Wisconsin 54-1255]. | | CAP94350 | 2e-13 |
| 1.84 | MGG_03439* | UR | N | | acid phosphatase [Aspergillus fumigatus Af293]. | | XP_746350 | 3e-79 |
| 1.81 | MGG_06414 | UR | N | | DUF185 domain protein [Aspergillus flavus NRRL3357]. | | EED52786 | 2e-134 |
| 1.81 | MGG_04190* | UR | N | | signal recognition particle, putative [Aspergillus flavus NRRL3357]. | | EED55590 | 7e-137 |
| 1.78 | MGG_02851 | UR | Y | | [Podospora anserina]. | | XP_001929723 | 2e-66 |
| 1.74 | MGG_04961* | UR | N | | vacuolar protein, putative [Candida dubliniensis CD36]. | | CAX40698 | 3e-25 |
| 1.74 | MGG_09199* | UR | Y | | histidine acid phosphatase, putative [Aspergillus clavatus NRRL 1]. | | XP_001275101 | 2e-126 |
| 1.73 | MGG_08454* | UR | Y | | necrosis- and ethylene-inducing protein 2 precursor [Botrytis tulipae]. | | ABB43262 | 2e-61 |
| 1.58 | MGG_08088 | UR | Y | | Pc21g14910 [Penicillium chrysogenum Wisconsin 54-1255]. | | CAP96388 | 5e-28 |
| 1.58 | MGG_11914 | UR | N | | Pc13g09950 [Penicillium chrysogenum Wisconsin 54-1255]. | | CAP92064 | 4e-22 |
| 1.58 | MGG_00062* | UR | N | | integral membrane protein [Talaromyces stipitatus ATCC 10500]. | | EED18561 | 2e-06 |
| 1.58 | MGG_03946 | UR | Y | | extracellular serine-rich protein [Aspergillus fumigatus Af293]. | | XP_754260 | 2e-09 |
| 1.58 | MGG_02937* | UR | N | | histone-lysine N-methyltransferase (Ash1), putative [Penicillium marneffei ATCC 18224]. | | XP_002152511 | 5e-88 |
| 1.58 | MGG_04209* | UR | Y | | related to exo-alpha-sialidase / neuraminidase [Neurospora crassa]. | | CAD70852 | 3e-19 |
| 1.58 | MGG_04535* | UR | Y | | esterase family protein [Aspergillus flavus NRRL3357]. | | EED55555 | 5e-39 |
| 1.58 | MGG_02648* | UR | N | | dynamin family GTPase, putative [Aspergillus fumigatus A1163]. | | EDP55311 | 8e-151 |
| 1.58 | MGG_09760* | UR | N | | F-box domain protein [Aspergillus clavatus NRRL 1]. | | XP_001272872 | 5e-08 |
| 1.58 | MGG_00763 | UR | N | | NIMA interactive protein [Emericella nidulans]. | | AAP23304 | 2e-80 |
| 1.58 | MGG_09394* | UR | N | | LRP16 family protein [Aspergillus fumigatus Af293]. | | XP_754239 | 1e-43 |
| 1.58 | MGG_04865* | UR | N | | krueppel c2h2-type zinc finger protein, putative [Penicillium marneffei ATCC 18224]. | | XP_00215261 | 6e-80 |
| 1.58 | MGG_03759 | UR | Y | | GPI transamidase component Gpi16, putative [Talaromyces stipitatus ATCC 10500]. | | EED23409 | 0.0 |
| 1.58 | MGG_07199* | UR | N | | related to myocyte-specific enhancer factor 2d [Neurospora crassa]. | | CAB91269 | 2e-06 |
| 1.58 | MGG_10019 | UR | N | | integral membrane protein [Penicillium marneffei ATCC 18224]. | | XP_002149317 | 4e-41 |
| 1.58 | MGG_06762* | UR | N | | ankyrin repeat-containing protein, putative [Penicillium marneffei ATCC 18224]. | | XP_002149104 | 3e-08 |
| 1.58 | MGG_00517* | UR | N | | pyridoxal kinase, putative [Aspergillus fumigatus A1163]. | | EDP55631 | 9e-97 |
| 1.58 | MGG_02296* | UR | Y | | DNA-directed DNA polymerase. | | AAB60597 | 1e-07 |
| 1.58 | MGG_03275 | UR | Y | | related to dock180 protein [Neurospora crassa]. | | CAE75725 | 0.0 |
| 1.58 | MGG_00288 | UR | N | | G-patch domain protein (TFIP11), putative [Talaromyces stipitatus ATCC 10500]. | | EED24211 | 5e-116 |
| 1.58 | MGG_10747* | UR | N | | Pc22g17450 [Penicillium chrysogenum Wisconsin 54-1255]. | | CAP99033 | 3e-26 |
| 1.58 | MGG_12016 | UR | Y | | acetylxylan esterase precursor [Pyrenophora tritici-repentis Pt-1C-BFP]. | | XP_001935292 | 9e-24 |
| 1.58 | MGG_07081* | UR | N | | PAXNEB protein superfamily [Talaromyces stipitatus ATCC 10500]. | | EED22978 | 7e-79 |
| 1.58 | MGG_09029 | UR | N | | N2,N2-dimethylguanosine tRNA methyltransferase [Talaromyces stipitatus ATCC 10500] | | EED14199 | 1e-39 |
| 1.58 | MGG_01543* | UR | N | | JmjC domain protein, putative [Talaromyces stipitatus ATCC 10500]. | | EED22276 | 8e-64 |
| 1.58 | MGG_06323* | UR | N | | integral membrane protein [Aspergillus clavatus NRRL 1]. | | XP_001276041 | 6e-72 |
| 1.58 | MGG_04165* | UR | N | | DUF221 domain protein, putative [Talaromyces stipitatus ATCC 10500]. | | EED23543 | 0.0 |
| 1.58 | MGG_05188* | UR | N | | DUF1682 domain protein [Aspergillus clavatus NRRL 1]. | | XP_001271871 | 5e-111 |
| 1.58 | MGG_05567 | UR | N | | thioesterase family protein [Aspergillus flavus NRRL3357]. | | EED54400 | 3e-13 |
| 1.58 | MGG_01857* | UR | Y | | Pc18g04150 [Penicillium chrysogenum Wisconsin 54-1255]. | | CAP94639 | 1e-37 |
| 1.58 | MGG_13277 | UR | N | | YALI0A07623p [Yarrowia lipolytica]. | | XP_499845 | 6e-04 |
| 1.58 | MGG_10737 | UR | N | | Pc13g11120 [Penicillium chrysogenum Wisconsin 54-1255]. | | CAP92181 | 2e-04 |
| 1.58 | MGG_03522* | UR | Y | | rhomboid protein 2 [Neurospora crassa OR74A]. | | XP_959792 | 3e-90 |
| 1.58 | MGG_03276* | UR | Y | | DEHA2B16192p [Debaryomyces hansenii]. | | CAG85669 | 1e-74 |
| 1.58 | MGG_11170* | UR | N | | Pc20g00770 [Penicillium chrysogenum Wisconsin 54-1255]. | | CAP85406 | 1e-44 |
| 1.58 | MGG_06056 | UR | N | | YeeE/YedE family protein [Aspergillus clavatus NRRL 1]. | | XP_001273782 | 6e-51 |
| 1.58 | MGG_12094* | UR | N | | Pc14g01970 [Penicillium chrysogenum Wisconsin 54-1255]. | | CAP74338 | 1e-39 |
| 1.58 | MGG_08855* | UR | N | | serine-rich protein, putative [Neosartorya fischeri NRRL 181]. | | XP_001257695 | 1e-26 |
| 1.58 | MGG_03603 | UR | N | | bud site selection protein, putative [Candida dubliniensis CD36]. | | CAX41570 | 8e-06 |
| 1.58 | MGG_11750* | UR | Y | | allergen Asp F4 [Aspergillus fumigatus Af293]. | | XP_749515 | 4e-30 |
| 1.55 | MGG_13532* | UR | N | | RSC complex subunit (RSC1) [Aspergillus fumigatus Af293]. | | XP_755045 | 4e-167 |
| 1.53 | MGG_06583* | UR | N | | Predicted 3'-5' exonuclease (ISS) [Ostreococcus tauri]. | | CAL56246 | 3e-14 |
| 1.53 | MGG_10799* | UR | Y | | acid phosphatase PHO12 precursor [Pyrenophora tritici-repentis Pt-1C-BFP]. | | XP_001939949 | 1e-75 |
| 1.52 | MGG_04575* | UR | N | | Pc16g04410 [Penicillium chrysogenum Wisconsin 54-1255]. | | CAP93111 | 4e-107 |
| 1.52 | MGG_00393 | UR | N | | conserved leucine-rich repeat protein [Neosartorya fischeri NRRL 181]. | | XP_001262358 | 6e-120 |
| 1.52 | MGG_07465* | UR | N | | PWP2_NEUCR Periodic tryptophan protein 2 homolog [Gibberella zeae PH-1]. | | XP_387555 | 0.0 |
| 1.51 | MGG_11809* | UR | N | | Pc13g10940 [Penicillium chrysogenum Wisconsin 54-1255]. | | CAP92163 | 2e-145 |
| 1.50 | MGG_08345* | UR | N | | WD repeat containing protein pop1 [Pyrenophora tritici-repentis Pt-1C-BFP]. | | XP_001932984 | 0.0 |
| **Interaction With Host (GO:0051701 )** | | | | | | | | |
| 6.88 | MGG_03817* | DR | Y | | metalloprotease [Pleurotus ostreatus]. | | AAU94648 | 4e-26 |
| 1.58 | MGG_05807* | DR | N | | Pc21g02580 [Penicillium chrysogenum Wisconsin 54-1255]. | | CAP95155 | 2e-17 |
| 1.58 | MGG_06575* | DR | N | | zinc finger protein, putative [Talaromyces stipitatus ATCC 10500]. | | EED22939 | 2e-27 |
| 1.58 | MGG_05807 | DR | N | | Pc21g02580 [Penicillium chrysogenum Wisconsin 54-1255]. | | CAP95155 | 2e-17 |
| 1.58 | MGG_01767* | DR | N | | related to MAK32 protein [Neurospora crassa]. | | CAE85522 | 8e-112 |
| 1.79 | MGG_07314* | DR | N | | C2H2 finger domain protein, putative [Aspergillus clavatus NRRL1]. | | XP_001275629 | 7e-22 |
| 1.77 | MGG_08107* | DR | N | | Pc21g02640 [Penicillium chrysogenum Wisconsin 54-1255]. | | CAP95161 | 6e-58 |
| 1.74 | MGG_06169* | DR | Y | | tyrosinase, putative [Talaromyces stipitatus ATCC 10500]. | | XP_370095 | 8e-112 |
| 1.74 | MGG_09381* | DR | N | | Pc21g03780 [Penicillium chrysogenum Wisconsin 54-1255]. | | CAP95275 | 9e-08 |
| 1.74 | MGG_09522* | DR | N | | beta-N-acetylglucosaminidase [Aspergillus fumigatus Af293]. | | XP_747213 | 9e-19 |
| 1.68 | MGG_06151* | DR | N | | protein NBP35 [Neurospora crassa OR74A]. | | XP_962036 | 6e-165 |
| 1.58 | MGG_02331* | DR | N | | integral membrane protein [Aspergillus clavatus NRRL 1]. | | XP_001267900 | 3e-08 |
| 1.58 | MGG_02410* | DR | N | | stomatin-like protein [Gibberella fujikuroi]. | | BAB68403 | 1e-150 |
| 1.58 | MGG_07334 | DR | Y | | Pc18g01680 [Penicillium chrysogenum Wisconsin 54-1255]. | | CAP94392 | 2e-06 |
| 1.58 | MGG_06912* | DR | N | | Pc20g00610 [Penicillium chrysogenum Wisconsin 54-1255]. | | CAP85390 | 4e-68 |
| 1.58 | MGG_04182 | DR | N | | MATE efflux family protein subfamily, putative [Aspergillus clavatus NRRL 1]. | | XP_001268211 | 9e-171 |
| 1.58 | MGG_06917* | DR | N | | GTP cyclohydrolase II [Pyrenophora tritici-repentis Pt-1C-BFP]. | | XP_001931331 | 0.0 |
| 4.81 | MGG_09134* | DR | Y | | hydrophobin [Gibberella moniliformis]. | | AAN76355 | 8e-18 |
| 4.52 | MGG_03977 | DR | N | | CON1 [Magnaporthe grisea]. | | ABB89847 | 0.0 |
| 4.52 | MGG_02127 | DR | N | | alcohol dehydrogenase [Pyrenophora tritici-repentis Pt-1C-BFP]. | | XP_001941560 | 4e-130 |
| 4.45 | MGG_08453 | DR | N | | hypothetical protein CHGG_05909 [Chaetomium globosum CBS 148.51]. | | XP_001222004 | 4e-29 |
| 4.00 | MGG_02065* | DR | N | | kinesin light chain [Pyrenophora tritici-repentis Pt-1C-BFP]. | | XP_001942318 | 0.0 |
| 3.90 | MGG_07402* | DR | N | | Pc13g06880 [Penicillium chrysogenum Wisconsin 54-1255]. | | CAP91757 | 1e-18 |
| 3.81 | MGG_02531 | DR | Y | | subtilisin-like serine protease [Verticillium dahliae]. | | AAS45251 | 1e-151 |
| 3.81 | MGG_08046* | DR | Y | | bilirubin oxidase precursor [Neurospora crassa OR74A]. | | XP_956350 | 0.0 |
| 3.75 | MGG_03392* | DR | Y | | benzoate 4-monooxygenase cytochrome P450 [Neosartorya fischeri NRR 181]. | | XP_001265538 | 1e-103 |
| 3.69 | MGG_10574* | DR | N | | GNAT family N-acetyltransferase [Aspergillus fumigatus Af293]. | | XP_001481585 | 4e-27 |
| 3.58 | MGG_14097* | DR | N | | Pc22g19420 [Penicillium chrysogenum Wisconsin 54-1255]. | | CAP99230 | 7e-32 |
| 3.58 | MGG_03332* | DR | N | | copper methylamine oxidase precursor [Pyrenophora tritici-repentis Pt-1C-BFP]. | | XP_001932394 | 0.0 |
| 3.17 | MGG_04170* | DR | N | | Pc12g06150 [Penicillium chrysogenum Wisconsin 54-1255]. | | CAP80242 | 1e-27 |
| 3.00 | MGG_04327 | DR | N | | zinc finger (MYND type) family protein [Arabidopsis thaliana]. | | NP_199856 | 5e-05 |
| 3.00 | MGG_07246* | DR | Y | | Pc20g02770 [Penicillium chrysogenum Wisconsin 54-1255]. | | CAP85606 | 9e-05 |
| 2.94 | MGG_01293* | DR | N | | Pc20g02710 [Penicillium chrysogenum Wisconsin 54-1255]. | | CAP85600 | 8e-62 |
| 2.81 | MGG_04522 | DR | N | | Pc12g02580 [Penicillium chrysogenum Wisconsin 54-1255]. | | CAP79885 | 1e-10 |
| 2.77 | MGG_01515* | DR | Y | | Pc16g04430 [Penicillium chrysogenum Wisconsin 54-1255]. | | CAP93113 | 7e-12 |
| 2.66 | MGG_09188 | DR | Y | | malate dehydrogenase [Aspergillus fumigatus Af293]. | | XP_755713 | 3e-34 |
| 2.58 | MGG_06059* | DR | N | | arrestin (or S-antigen), N-terminal domain protein [Aspergillus fumigatus Af293]. | | XP_751214 | 2e-47 |
| 2.35 | MGG_08373* | DR | Y | | hypothetical protein FG03600.1 [Gibberella zeae PH-1]. | | XP_383776 | 2e-53 |
| 2.35 | MGG_01679* | DR | N | | ThiJ/PfpI family protein [Penicillium marneffei ATCC 18224]. | | XP_002153446 | 6e-68 |
| 2.32 | MGG_07673 | DR | N | | hypothetical protein [Magnaporthe oryzae 70-15] | | XP_367762 | 0 |
| 2.32 | MGG_13573* | DR | N | | FAD binding oxidoreductase, putative [Aspergillus fumigatus A1163]. | | EDP50847 | 4e-63 |
| 2.22 | MGG_01017 | DR | N | | C2H2 finger domain protein, putative [Talaromyces stipitatus ATCC 10500]. | | EED17685 | 1e-16 |
| 2.00 | MGG_04481* | DR | N | | tetratricopeptide repeat domain 24 [Mus musculus]. | | NP_766114 | 0.49 |
| 2.00 | MGG_05529* | DR | Y | | feruloyl esterase B precursor [Pyrenophora tritici-repentis Pt-1C-BFP]. | | XP_001936734 | 6e-143 |
| 2.00 | MGG_15241 | DR | Y | | alpha/beta hydrolase fold protein [Aspergillus clavatus NRRL 1]. | | XP_001268813 | 4e-37 |
| 2.00 | MGG_07787 | DR | Y | | regulatory P domain-containing protein [Hahella chejuensis KCTC 2396]. | | YP_433708 | 1e-60 |
| 1.87 | MGG_13620* | DR | N | | F-box domain protein [Aspergillus flavus NRRL3357]. | | EED55088 | 3e-16 |
| 1.85 | MGG_06392 | DR | N | | ornithine aminotransferase [Neurospora crassa OR74A]. | | XP_956417 | 0.0 |
| 1.81 | MGG_13218* | DR | N | | pyruvate formate lyase activating enzyme, putative [Neosartorya fischeri NRRL 181]. | | XP_001261025 | 1e-121 |
| 3.91 | MGG_10408* | UR | Y | | Pc20g09930 [Penicillium chrysogenum Wisconsin 54-1255]. | | CAP86322 | 1e-95 |
| 4.17 | MGG_13971* | UR | N | | DUF563 domain protein [Aspergillus fumigatus Af293]. | | XP_751886 | 3e-45 |
| 3.46 | MGG_08750* | UR | Y | | Pc22g14360 [Penicillium chrysogenum Wisconsin 54-1255]. | | CAP98724 | 8e-84 |
| 2.32 | MGG_10305* | UR | N | | Pc21g17770 [Penicillium chrysogenum Wisconsin 54-1255]. | | CAP96674 | 1e-78 |
| 2.00 | MGG_01371* | UR | Y | | predicted protein [Botryotinia fuckeliana B05.10]. | | XP_001560550 | 5e-14 |
| 2.00 | MGG_06839* | UR | N | | kinesin [Gibberella moniliformis]. | | AAO59303 | 0.0 |
| 2.00 | MGG_13065 | UR | N | | related to protein GRR1 [Neurospora crassa]. | | CAD21405 | 0.0 |
| 2.00 | MGG_01352* | UR | Y | | unnamed protein product [Podospora anserina]. | | XP_001910809 | 2e-21 |
| 4.39 | MGG_10275* | UR | Y | | acid sphingomyelinase, putative [Penicillium marneffei ATCC 18224]. | | XP_002145882 | 0.0 |
| 2.00 | MGG_03995* | UR | Y | | carboxypeptidase S1, putative [Aspergillus clavatus NRRL 1]. | | XP_001274058 | 5e-121 |
| 1.81 | MGG_14951* | UR | N | | DNA repair protein Rad26 [Aspergillus fumigatus Af293]. | | XP_750448 | 6e-06 |
| 1.81 | MGG_06447 | UR | N | | SNF2 family helicase/ATPase, putative [Aspergillus fumigatus A1163]. | | EDP51409 | 9e-151 |
| 1.81 | MGG_15423* | UR | Y | | extracelular serine carboxypeptidase, putative [Talaromyces stipitatus ATCC 10500] | | EED18491 | 3e-148 |
| 1.62 | MGG_00733* | UR | N | | Pc21g20850 [Penicillium chrysogenum Wisconsin 54-1255]. | | CAP96982 | 4e-39 |
| 1.58 | MGG_07890* | UR | N | | aldehyde dehydrogenase 3F1 [Pyrenophora tritici-repentis Pt-1C-BFP]. | | XP_001936067 | 2e-145 |
| 1.58 | MGG_03738* | UR | N | | F-box domain protein [Aspergillus clavatus NRRL 1]. | | XP_001268367 | 2e-66 |
| 1.58 | MGG_07011* | UR | N | | C2H2 finger domain protein, putative [Penicillium marneffei ATCC 18224]. | | XP_002150081 | 5e-08 |
| 1.58 | MGG_04406* | UR | N | | ankyrin repeat protein [Trichomonas vaginalis G3]. | | XP_001317124 | 6e-22 |
|  |  |  |  | |  | |  |  |
| **GO:0009847 : spore germination** | | | | | | | | |
| 1.81 | MGG_08158* | DR | Y | | putative protein [Neurospora crassa]. | | CAD11419 | 1e-41 |
| 1.75 | MGG_14061* | DR | Y | | oxalate decarboxylase, putative [Aspergillus flavus NRRL3357]. | | EED56627 | 3e-144 |
| [**GO:0009987 : Cellular process**](http://amigo.geneontology.org/cgi-bin/amigo/term-details.cgi?term=GO:0009987&session_id=7679amigo1236868689) | | | | | | | | |
| 1.58 | MGG_01350* | DR | Y | | transmembrane glycoprotein, putative [Aspergillus flavus NRRL3357]. | | EED58154 | 3e-58 |
| 1.52 | MGG_06993* | DR | N | | HIRA-interacting protein 5 [Neurospora crassa OR74A]. | | XP_957202 | 4e-116 |
| 3.32 | MGG_03994* | UR | N | | chromosome segregation protein Cse1, putative [Aspergillus clavatus NRRL 1]. | | XP_001270127 | 0.0 |
| 2.00 | MGG_04616* | UR | N | | Epsin-like protein ent1/2 [Neurospora crassa OR74A]. | | XP_960232 | 2e-163 |
| 1.65 | MGG_10322* | UR | N | | actin cortical patch protein Sur7, putative [Penicillium marneffei ATCC 18224]. | | XP_002151694 | 9e-26 |
| 1.58 | MGG_01080* | UR | N | | Chromo domain protein Chp1p, putative [Penicillium marneffei ATCC 18224]. | | XP_002146321 | 2e-05 |
| 1.58 | MGG_04912 | UR | N | | chromosome segregation protein BIR1, putative [Talaromyces stipitatus ATCC 10500]. | | EED22299 | 1e-27 |
| 1.58 | MGG_03693* | UR | N | | chromosome segregation protein Spc105 [Aspergillus fumigatus Af293]. | | XP_749546 | 2e-180 |
| 1.58 | MGG_06628* | UR | N | | sorting nexin Mvp1 [Aspergillus flavus NRRL3357]. | | EED47591 | 0.0 |
| 1.58 | MGG_01702* | UR | N | | MAP kinase activator [Ophiostoma piceae]. | | ABP01776 | 0.0 |
| 1.58 | MGG_05991 | UR | N | | 26S proteasome regulatory subunit Rpn2, putative [Aspergillus clavatus NRRL 1]. | | XP_001271741 | 0.0 |

a Genes ID with asterisk at top right corner indicating the putative MoAP1 binding site that identified in the promoter region of these genes.

b Gene expression was marked as upregulated (UR) and down regulated(DR).

c Signal peptide cleavage sites were predicted by SignalP 3.0 (www.cbs.dtu.dk/services/SignalP/)

d E-values were taken from BLASTX search against NCBI non redundant protein database.
