# Supplementary material for: The bZIP Transcription Factor MoAP1 Mediates the Oxidative Stress Response and Is Critical for Pathogenicity of the Rice Blast Fungus Magnaporthe oryzae
Source: PLoS Pathog. 2011 Feb 24;7(2):e1001302. doi: 10.1371/journal.ppat.1001302 (PMC3044703; doi:10.1371/journal.ppat.1001302)
Supplement: Table S2 — Primer pairs used in this paper. (0.14 MB DOC) [file ppat.1001302.s014.doc]

Table S2 Primer pairs used in this paper

| Primer name | | Primer Sequences (5’-3’) | Remark |
| --- | --- | --- | --- |
| FL2700 | | GAATTCATGGCTTCGTCACAGAGCGGTT | Amplification of *Moap1* cDNA |
| FL2701 | | GCATGCCTAGCTCGACGTCGCCGCACCT |
| FL1992 | | GAGTGAACCCATGAGTAGTAGT | *Moap1* deletion vector construction |
| FL 1993 | | TAATCGTCCTCAGTGCTTGCGAGTTTAAACAGTGCAGCGAAGAGCAGACTC |
| FL1994 | | GAGTCTGCTCTTCGCTGCACTGTTTAAACTCGCAAGCACTGAGGACGATTA |
| FL1995 | | TGTTGCCATCTACCCACAGTAT |
| FL2382 | | TGACCATGTCGCCGAATTCGTT | RT-PCR validation of *Moap1* |
| FL 2383 | | GAAGTCATTCTGGTCCACCACA |
| FL2384 | | CAGCAACAGAGTCTGCTCTT | Amplification of *Moap1* probe for Southern blot |
| FL 2385 | | TCCGTATTCTTGCCCAGGTA |
| FL2611 | | **GGATCC**GCCGTATGCCGCTCATATAGTA | Complemented vector construction of *Moap1* |
| FL2612 | | **AAGCTT**ATCACACCCGAGCCAAAATGAC |
| FL4801 | | ACCAACAACCTCACCAGGAG | MGG_09139  QRT |
| FL4802 | | GCTGGACCGTTGATGACGAT |
| FL4795 | | GGAGTACTGGCTCTCCATCG | MGG_11608  QRT |
| FL4796 | | TTGGTCTGCATGTTGTTGGT |
| FL4789 | | CTTCCAGTACGAGGGTGCT | MGG_13464  QRT |
| FL4790 | | TGTTGACGGTCCAGTAGACG |
| FL4797 | | CGAATCAACGGCGATATCTT | MGG_08865  QRT |
| FL4798 | | GGGAGTAGGTATCCCGAAGC |
| FL4803 | | TCCCTGGACTGCTCAAGTCT | MGG_01924  QRT |
| FL4804 | | GCTGTCAAGAGGACGGTAGC |
| FL4799 | | CCACGAGCTCAACTTTGGAT | MGG_04404  QRT |
| FL4800 | | GGACGGTGACAAGCATCTCT |
| FL4819 | | TTACCGTGGCCGACTTTTAC | MGG_05565  QRT |
| FL4820 | | TCTGGGAATTGGAGCTTGAG |
| FL4815 | | CATGATGCCCTCAGGAAACT | MGG_12228  QRT |
| FL4816 | | GCAACATGGTCACCACAGTC |
| FL4821 | | GTGGGAGTTTGTTCGCTCAT | MGG_01230  QRT |
| FL4822 | | GACCGTCTTGGTGTTGGAGT |
| FL4825 | | TCTCCCTCAAGACCGTTGTC | MGG_10315  QRT |
| FL4826 | | GATCGGGATGTTCTTGCACT |
| FL6130 | | CCCTCAGCCCACATACAACT | MGG_03977  QRT |
| FL6131 | | GCCAACTTGAGGCATACCAT |
| FL5884 | | GAGGAGCAGCATGGACTCTC | MGG_12814 (*Moap1*)  QRT |
| FL5885 | | CCACCGAGTCCTTGATCATT |
| **MGG_01230** | | | |
| FL4226 | AAATGTTGGTTCAGGCTGCCAC | | *Mossadh* deletion vector construction |
| FL4227 | TATATCCACATCACCCCTGCAGGTTTAAACTGCATTCCTGTGCATGCGAATG | |
| FL4228 | CATTCGCATGCACAGGAATGCAGTTTAAACCTGCAGGGGTGATGTGGATATA | |
| FL4229 | TTTCTTGTACGCGTCCTGGAAG | |
| FL4230 | TCGCGACACTTATTACGTGGGA | | validation of *Mossadh* deletion |
| FL4231 | CGACATCTCCGGTGTCATATCA | |
| FL6747 | **gtcgac**ATGTCTGCCATTCGCATGCACA | | Complemented vector construction of *Mossadh* |
| FL6748 | **ggtacc**TATATCCACATCACCCCTGCAG | |
| FL6745 | CTGCCATGGGCACACGTAAGAT | | RT-PCR validation of *Mossadh* |
| FL6746 | TAGTTTCAGGGTCGAACCCACT | |
| **MGG_15157** | | | |
| FL4280 | GTCAACGTCGTCATTAGCATCC | | *Moact* deletion vector construction |
| FL4281 | AGCTCACACTGTGCCTCCATCAGTTTAAACATTGGGCTGTAGGGCTATCTGT | |
| FL4282 | ACAGATAGCCCTACAGCCCAATGTTTAAACTGATGGAGGCACAGTGTGAGCT | |
| FL4283 | CTACTACTACTACTCCTCCCTC | |
| FL4284 | TCGCGACACTTATTACGTGGGA | | validation of *Moact* deletion |
| FL4285 | CGACATCTCCGGTGTCATATCA | |
| FL6749 | ACGCAGACCGTCTTTCCTGAAT | | RT-PCR validation of *Moact* |
| FL6750 | TAAAGTCCTGGCTCACAACCCT | |
| FL6751 | ggatccGTCAACGTCGTCATTAGCATCC | | Complemented vector construction of *Moact* |
| FL6752 | aagcttTGCACTTGTATTACGAGGACAG | |
| FL1111 | GGAGGTCAACACATCAATG | | PCR amplification for *HPH* gene |
| FL1112 | CTCTATTCCTTTGCCCTCG | |
| FL474 | TCGACGTCCGAAAGGATCTGT | | Actin gene amplification during RT-PCR validation |
| FL475 | ACTCCTGCTTCGAGATCCACATC | |
| FL4362 | CCATGTACCCTGGTCCCTTTCG | | Actin gene amplification during qRT-PCR validation |
| FL4363 | TTCGAGATCCACATCTGCTG | |
| FL 3935 | ACGTCGAGGAGGACCAAAGAAT | | MGG_13654 deletion vector construction |
| FL 3936 | GTGGACTAGACTAACAGTCTCCGTTTAAACAAATCTGGTTGGCTGGAAGTGG | |
| FL 3937 | CCACTTCCAGCCAACCAGATTTGTTTAAACGGAGACTGTTAGTCTAGTCCAC | |
| FL 3938 | AGCCTCTCAGGTTATGGGTTTG | |
| FL 3939 | GTCGCAGGCAATGTATGCAGAT | | validation of MGG_13654 deletion |
| FL 3940 | GGTGCAAGTGACACATGAGATG | |
| FL3941 | CAGCCAGTGCAGACAATTCAGA | | MGG_14966 deletion vector construction |
| FL3942 | GACGTTCCACTAAGAGTCGAGTGTTTAAACACCACAGAGTTAGGATATCGGA | |
| FL3943 | TCCGATATCCTAACTCTGTGGTGTTTAAACACTCGACTCTTAGTGGAACGTC | |
| FL3944 | AAGAGATATCTGGAGGACCTCG | |
| FL3945 | AATCATCGAGAGGTGCACTCAG | | validation of MGG_14966 deletion |
| FL3946 | CAACTTACCTGCAGCAGTTTCC | |
| FL4057 | CCTTTGAGCGTCAGGATCATCA | | MGG_13464 deletion vector construction |
| FL4058 | GTTTTCCTTTTCAGACTGCGCGGTTTAAACGCTGGTGACCTTCATGATGAGA | |
| FL4059 | TCTCATCATGAAGGTCACCAGCGTTTAAACCGCGCAGTCTGAAAAGGAAAAC | |
| FL4060 | CCAGCATTTCCACCGTCGTTTA | |
| FL4061 | CAACACCGACTATGAGGACAAG | | validation of MGG_13464 deletion |
| FL4062 | GGGATCAGTCTGTGATGGAAGT | |
| FL4232 | CTATTTTTTGGCGTGACAGGGG | | MGG_02378 deletion vector construction |
| FL4233 | CATTAGCACACCGCATGTGTCTGTTTAAACGGAGATGGACTTTCTTGACACC | |
| FL4234 | GGTGTCAAGAAAGTCCATCTCCGTTTAAACAGACACATGCGGTGTGCTAATG | |
| FL4235 | CACCCTCTTCAGCCTCGAATAC | |
| FL4236 | AAGAAGCAGAGAAGCTCATGGC | | validation of MGG_02378 deletion |
| FL4237 | CAGACGGATGAGCTGGTAGTAC | |
| FL4238 | ATCCAATTCACGAAGCGGAGTG | | MGG_02531 deletion vector construction |
| FL4239 | ATACTTGCCACGGACCTCAAACGTTTAAACTGGAGAGGATTGTGCTGCGCAT | |
| FL4240 | ATGCGCAGCACAATCCTCTCCAGTTTAAACGTTTGAGGTCCGTGGCAAGTAT | |
| FL4241 | ATCTTTGGTCGCAGCACTCAGA | |
| FL4242 | TGACGATCCGATTGACAGCAAC | | validation of MGG_02531 deletion |
| FL4243 | CAGACGGATGAGCTGGTAGTAC | |
| FL4244 | AAACGGGTTATGCAGGGGTCTT | | MGG_03817 deletion vector construction |
| FL4245 | TTAAATTCAGCGGTCCAGCCCAGTTTAAACCGAGTATGGTTGACCTGAGATG | |
| FL4246 | CATCTCAGGTCAACCATACTCGGTTTAAACTGGGCTGGACCGCTGAATTTAA | |
| FL4247 | GTCGATTTCAAGTCTGCCTACG | |
| FL4248 | AAGACGTCTGCTGGACTACGTC | | validation of MGG_03817 deletion |
| FL4249 | ACTAAGAGACACGTCAACCCTG | |
| FL4268 | CGCTACTTCATACCAAGGTAGG | | MGG_10422 deletion vector construction |
| FL4269 | TGCGTTTGTATGATGTGTGGCGGTTTAAACCCGTCAACTACTGAAGGTAGAG | |
| FL4270 | CTCTACCTTCAGTAGTTGACGGGTTTAAACCGCCACACATCATACAAACGCA | |
| FL4271 | TGACCAACATCACCCTCTACCA | |
| FL4272 | TTCTGGCTTGACAGGGGCTTAT | | validation of MGG_10422 deletion |
| FL4273 | ATGTTGACTTGGGAGGAGTGTG | |
| FL6318 | TATATGCGTGCCTCTTGTGGCT | | MGG_01662 deletion vector construction |
| FL6319 | TCGAGCAGGATGTTGGCATGTTGTTTAAACATGTGTGTGTTGTAGCTGGGAG | |
| FL6320 | CTCCCAGCTACAACACACACATGTTTAAACAACATGCCAACATCCTGCTCGA | |
| FL6321 | AGGATGAGCTCAGGCTAGAGAA | |
| FL6322 | CAGTCTGAACATGCTCACAG | | validation of MGG_01662 deletion |
| FL6323 | GACATGATTGAGAGCTCCTG | |
