# Supplementary figures and images for: Correction: The bZIP Transcription Factor MoAP1 Mediates the Oxidative Stress Response and is Critical for Pathogenicity of the Rice Blast Fungus Magnaporthe oryzae
Source: PLoS Pathog. 2019 Nov 20;15(11):e1008196. doi: 10.1371/journal.ppat.1008196 (PMC6867591; doi:10.1371/journal.ppat.1008196)

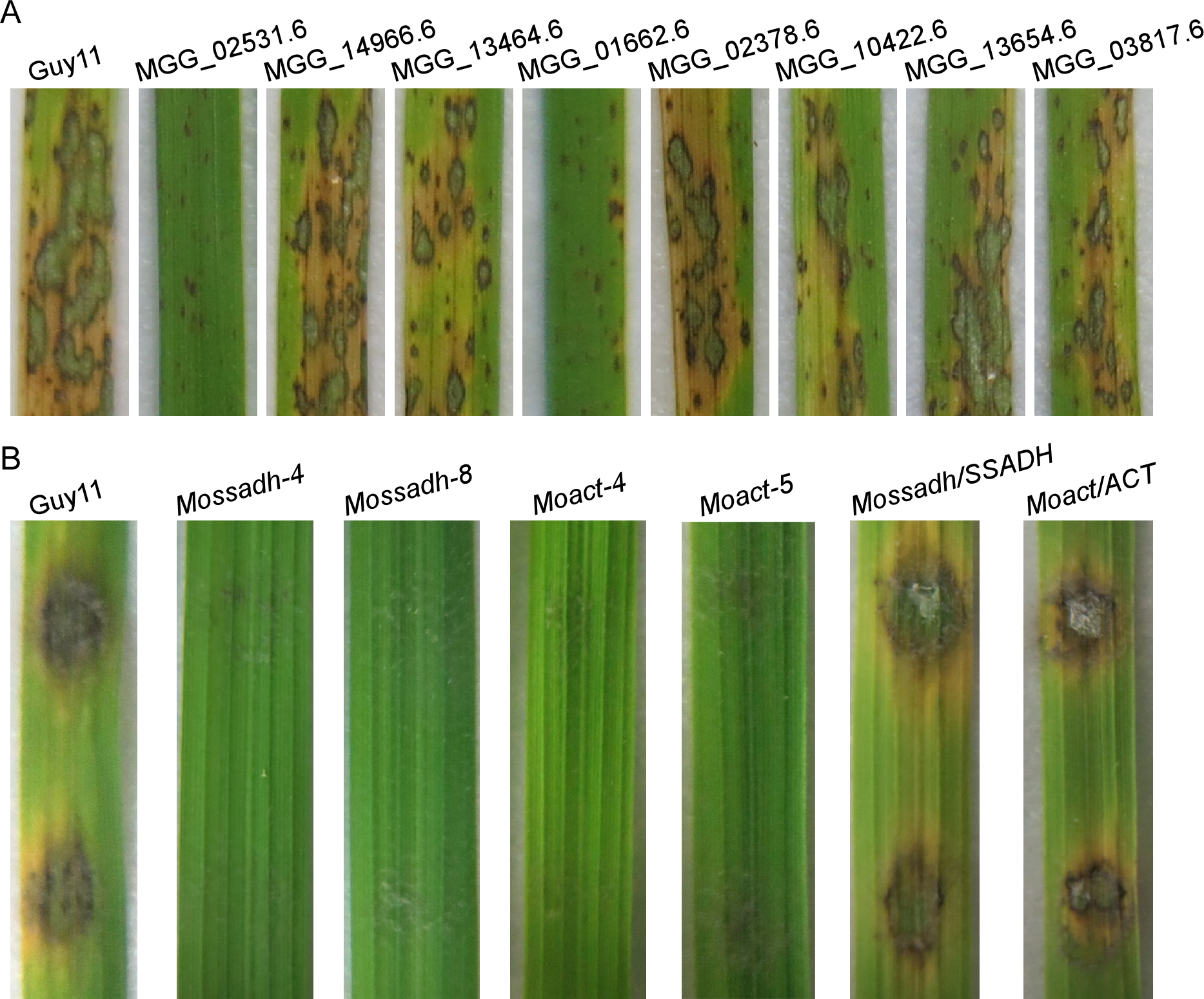

Supplement: S12 Fig — (A) Pathogenicity test of gene deletion mutants on the rice cultivar CO-39. The SAGE down-regulated gene deletion mutants were inoculated by spraying conidia suspensions on the four-week old rice cultivar CO-39 for 7 days and then photographed. (B) Pathogenicity test of Mossadh and Moact mutants on the rice cultivar CO-39 at 7 dpi with mycelial plugs. (TIF) [file ppat.1008196.s001.tif]
